# Supplementary material for: Regioselective Synthesis of NO-Donor (4-Nitro-1,2,3-triazolyl)furoxans via Eliminative Azide–Olefin Cycloaddition
Source: Molecules. 2023 Oct 7;28(19):6969. doi: 10.3390/molecules28196969 (PMC10574565; doi:10.3390/molecules28196969)
Supplement: Supplementary file 1 [file molecules-28-06969-s001.zip › molecules-2617205-supplementary.pdf]

Supporting information for

Regioselective Synthesis of NO-donor (4-Nitro-1,2,3-triazolyl)furoxans via Eliminative Azide-Olefin Cycloaddition

Irina A. Stebletsova [1,2], Alexander A. Larin [1] , Ivan V. Ananyev[3] and Leonid L. Fershtat [1,*]

[1] N.D. Zelinsky Institute of Organic Chemistry, Russian Academy of Sciences 47 Leninsky Prosp., 119991 Moscow, Russian Federation

[2] D.I. Mendeleev University of Chemical Technology of Russia, 9 Miusskaya Square, Moscow 125047, Russian Federation

[3] N.S. Kurnakov Institute of General and Inorganic Chemistry, Russian Academy of Sciences, GSP-1, Leninsky prospect, 31, 119991 Moscow, Russian Federation

* Correspondence: fershtat@bk.ru

Table of Contents

S1. Crystallographic data **2**

S2. Copies of NMR spectra **4**

S1. Crystallographic data

Table S1. The main crystallography data and refinement details for structures of 2**a**, **3a** and **3c**.

	**2a**	**3a**	**3c**
Formula unit	$C_9H_7N_5O_2$	$C_{11}H_8N_6O_4$	$C_{10}H_5BrN_6O_4$
Molecular mass	217.20	288.23	353.11
T, K	100	100	100
Crystal system	Monoclinic	Monoclinic	Orthorhombic
Space group	$P2_1/c$	$P2_1/c$	Pbca
Z (Z')	4 (1)	4 (1)	8 (1)
a, Å	9.1878(3)	14.5422(5)	10.5450(3)
b, Å	12.9430(3)	5.8853(2)	9.3289(3)
c, Å	8.0527(3)	14.5495(4)	26.0719(9)
α, °	90	90	90
β, °	95.337(3)	93.7410(10)	90
γ, °	90	90	90
V, $Å^3$	953.46(5)	1242.57(7)	2564.78(14)
d_{calc}, $g \cdot cm^{-3}$	1.513	1.541	1.829
Radiation	Cu K_α	Mo K_α	Mo K_α
μ, cm^{-1}	9.59	1.22	32.33
F(000)	448	592	1392
$2\theta_{max}$, °	135	60	60
Reflections measured	5834	16866	34822
Independent reflections	1833	3638	3821
Reflections with I>2σ(I)	1618	3041	3530
Number of parameters	146	222	200
R_1	0.0415	0.0397	0.0365
wR_2	0.1145	0.1085	0.0895
GOF	1.060	1.057	1.266
Residual electron density, $e \cdot Å^{-3}$ (d_{min}/d_{max})	0.180/-0.278	0.380/-0.218	0.506/-0.596

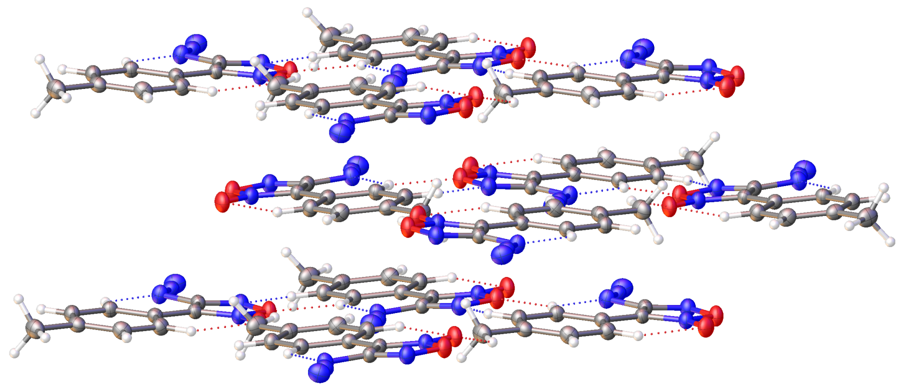

Figure S1. A fragment of layers in the crystal packing of **2a**.

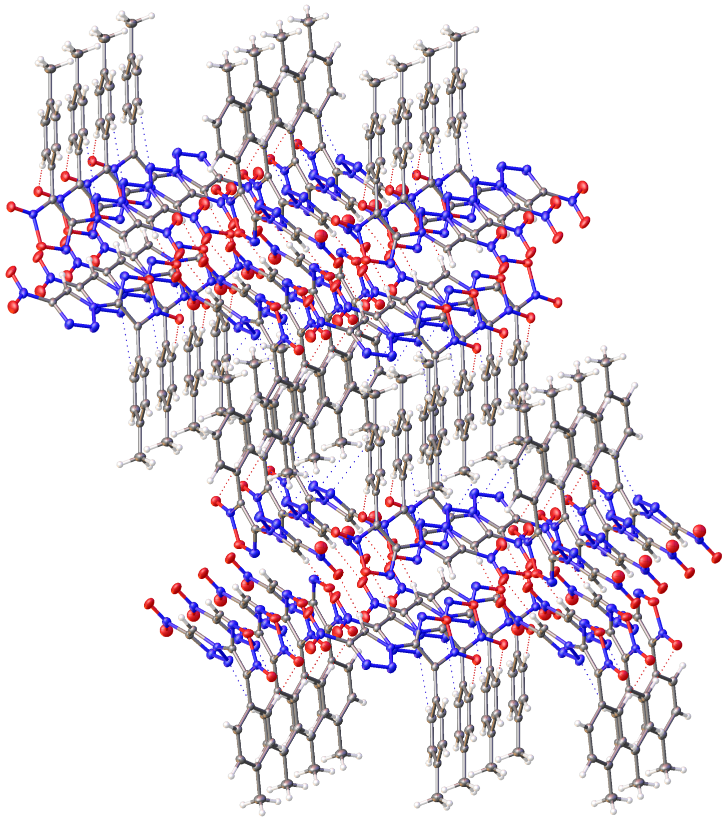

Figure S2. A fragment of layers in the crystal packing of **3a**.

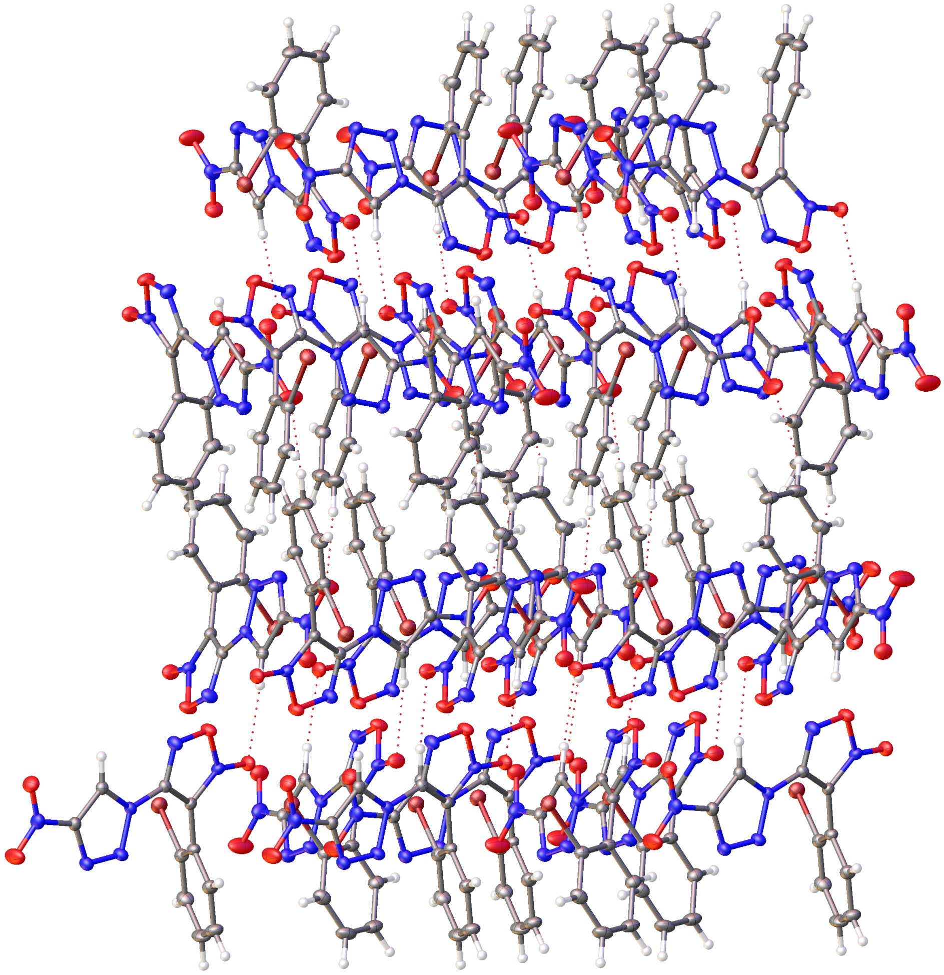

Figure S3. A fragment of layers in the crystal packing of **3c**.

S2. Copies of NMR spectra

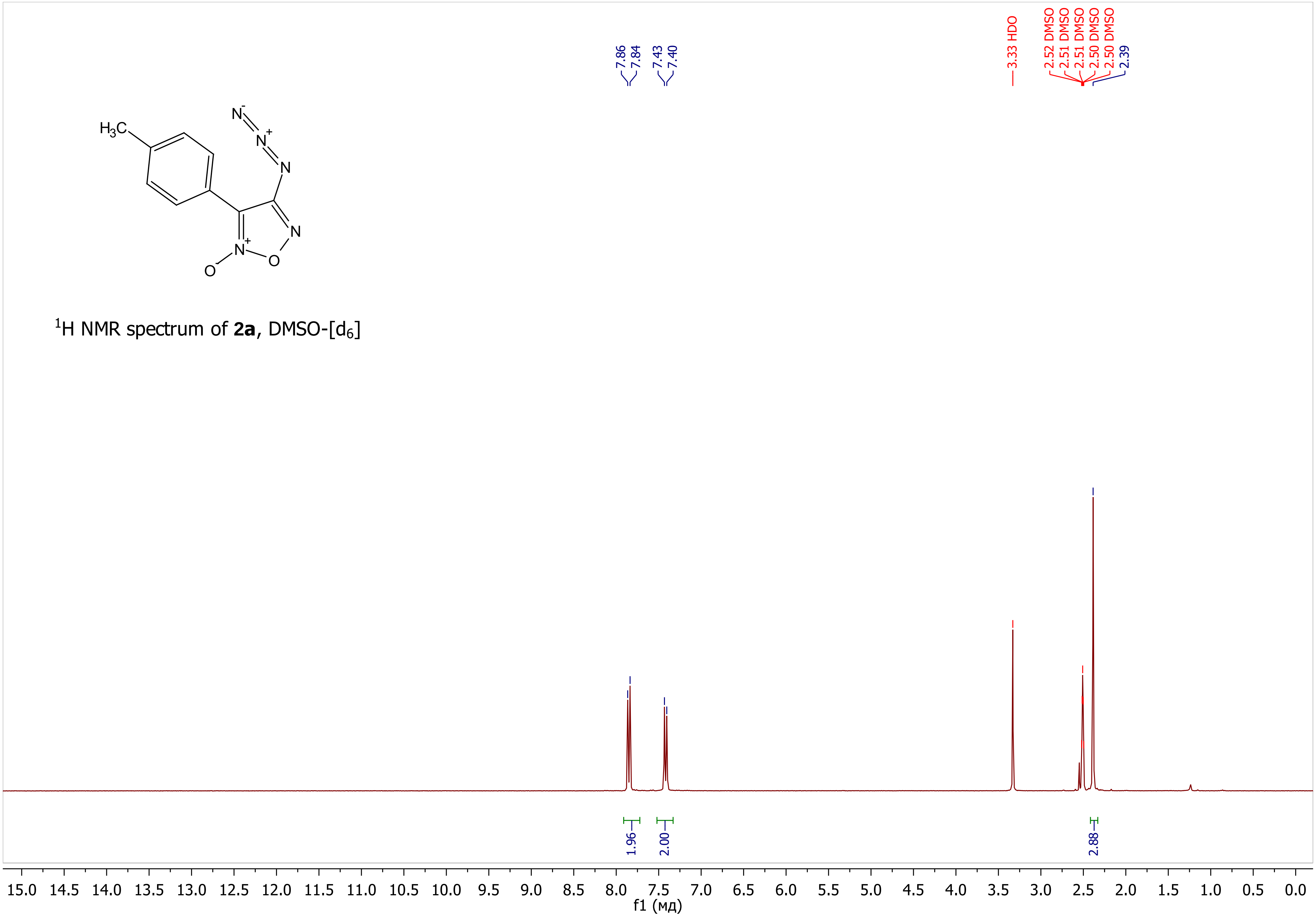

^{1}H NMR spectrum of **2a**, DMSO-[d_6]

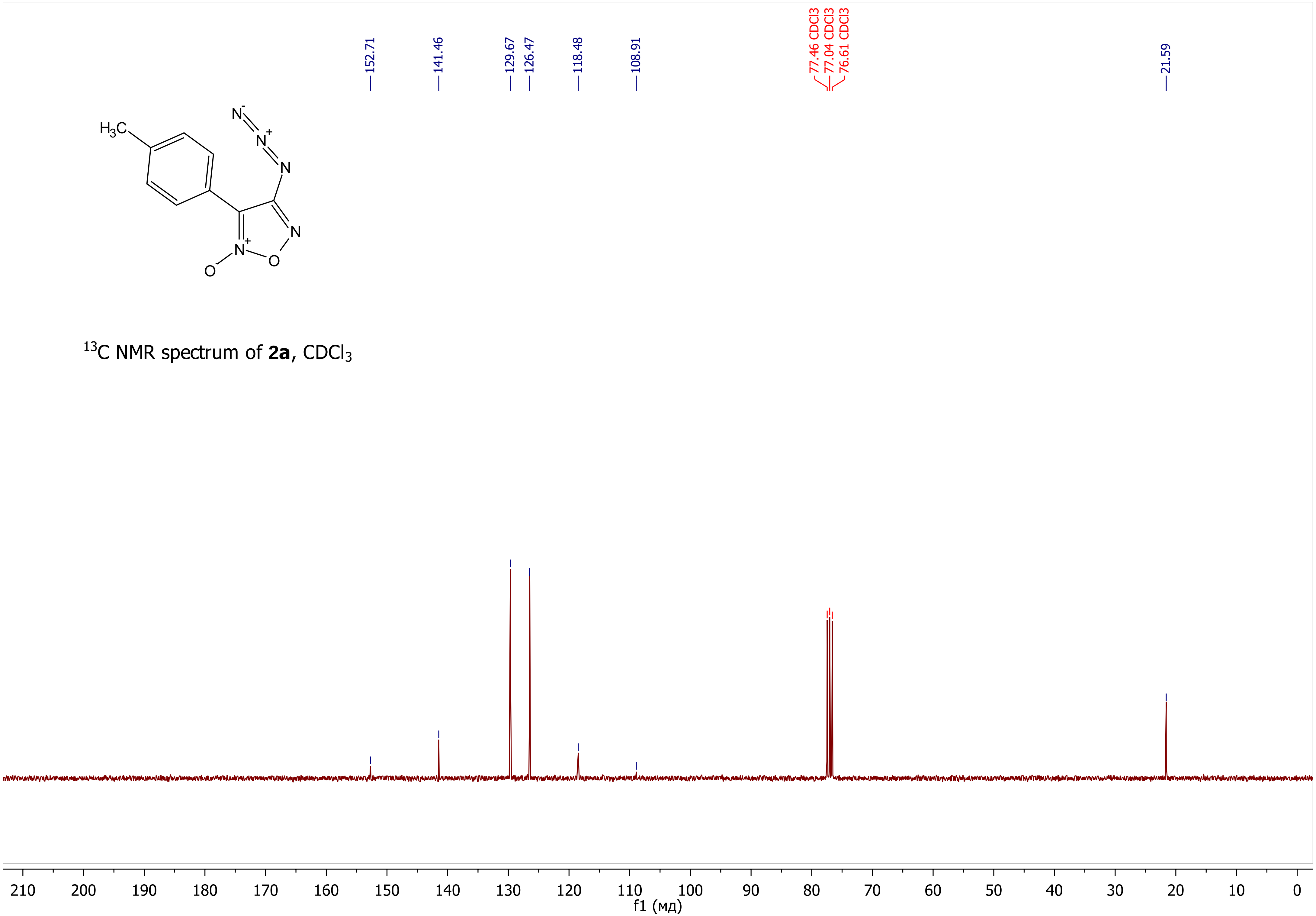

152.71
141.46
129.67
126.47
118.48
108.91
77.46 CDCl3
77.04 CDCl3
76.61 CDCl3
21.59
H3C
N
N
N
N
N
O
O
13C NMR spectrum of 2a, CDCl3
210
200
190
180
170
160
150
140
130
120
110
100
90
80
70
60
50
40
30
20
10
0
f1 (мд)

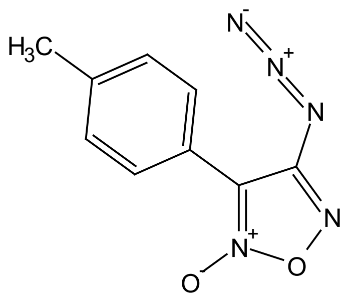

^{14}N NMR spectrum of **2a**, DMSO-[d$_6$]

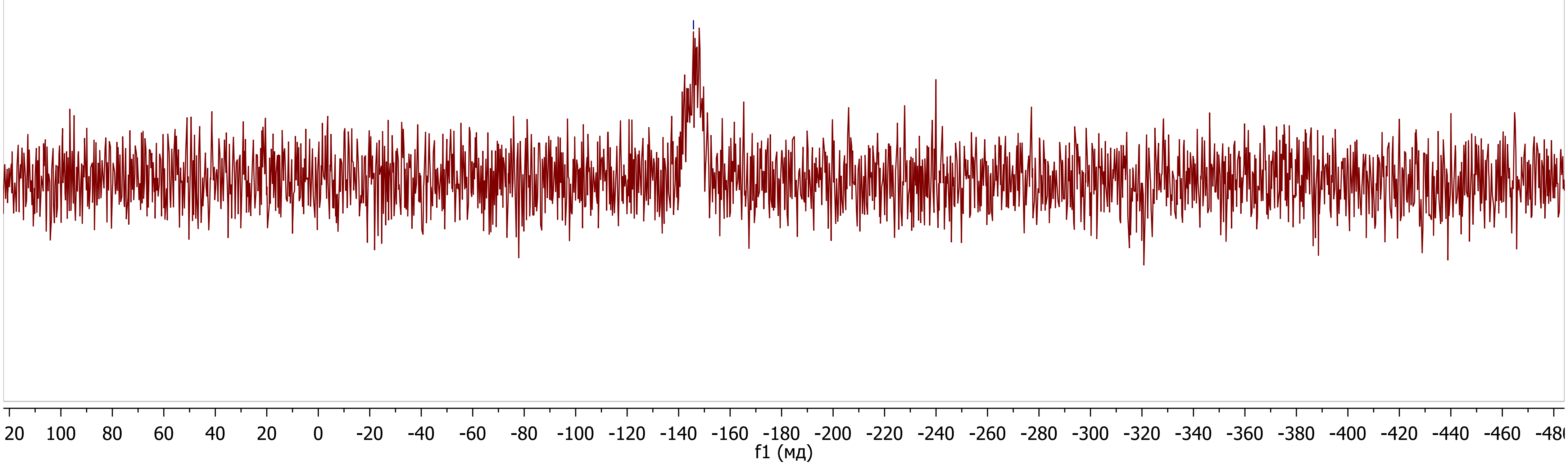

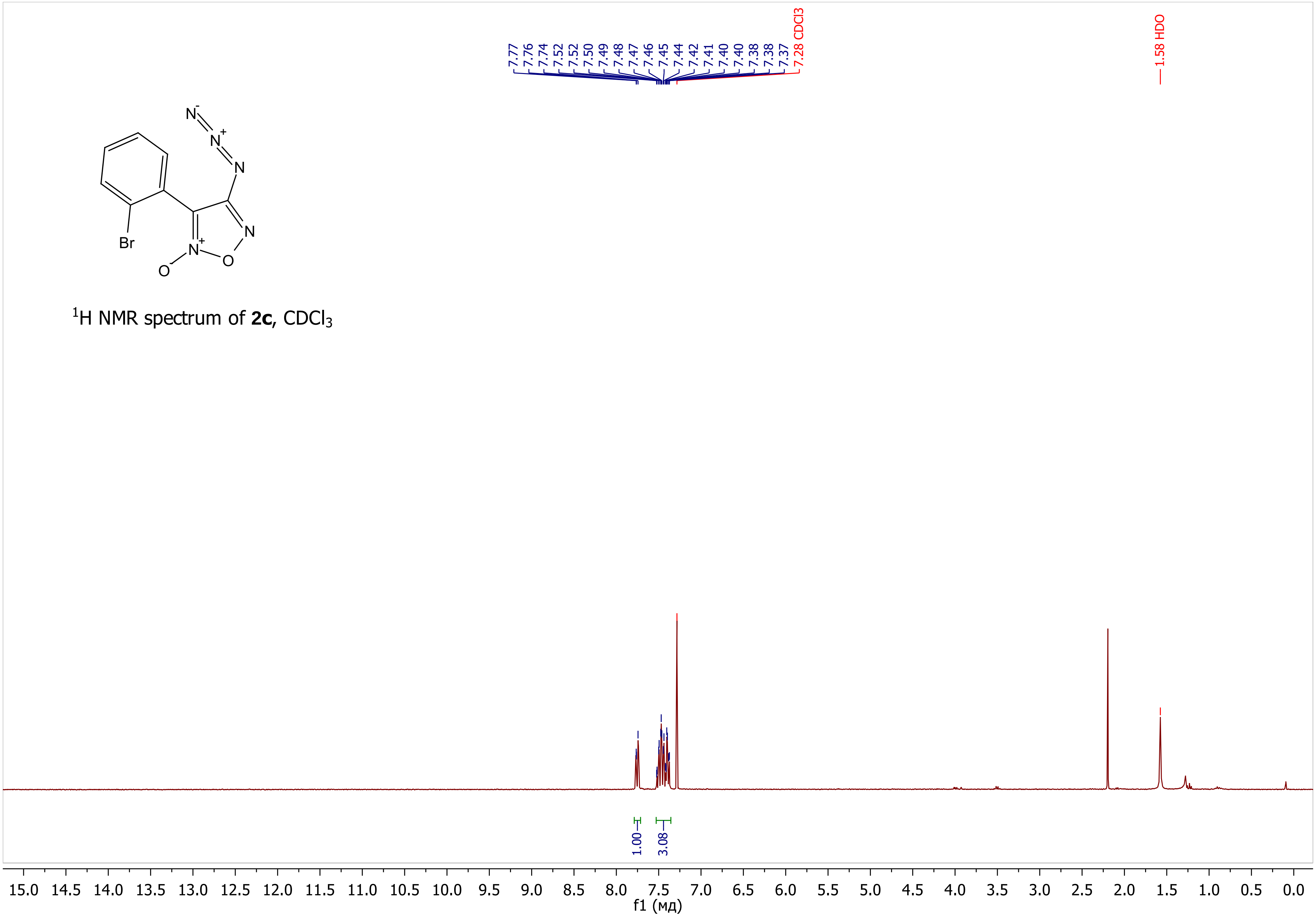

^{1}H NMR spectrum of **2c**, $CDCl_3$

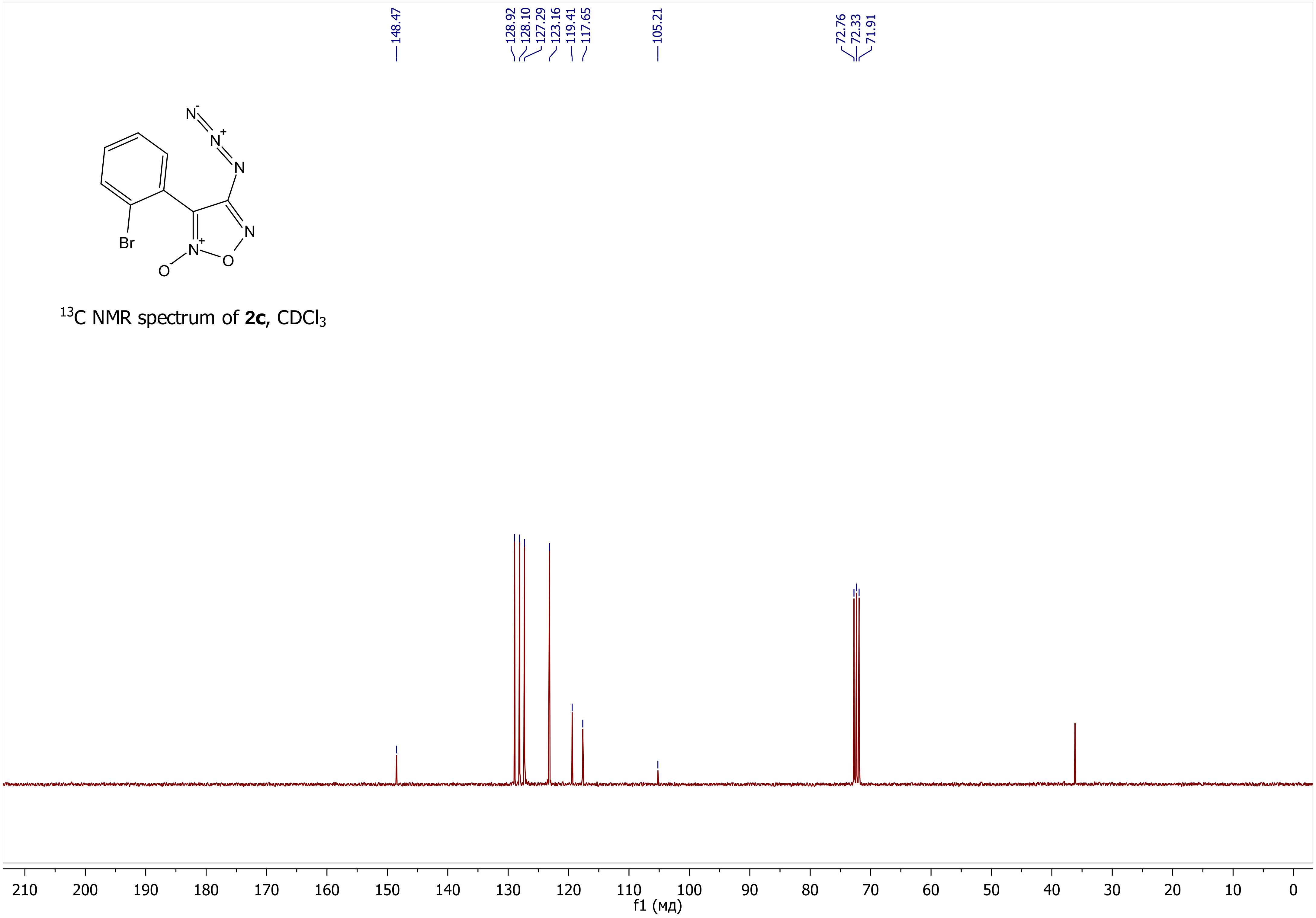

^{13}C NMR spectrum of **2c**, $CDCl_3$

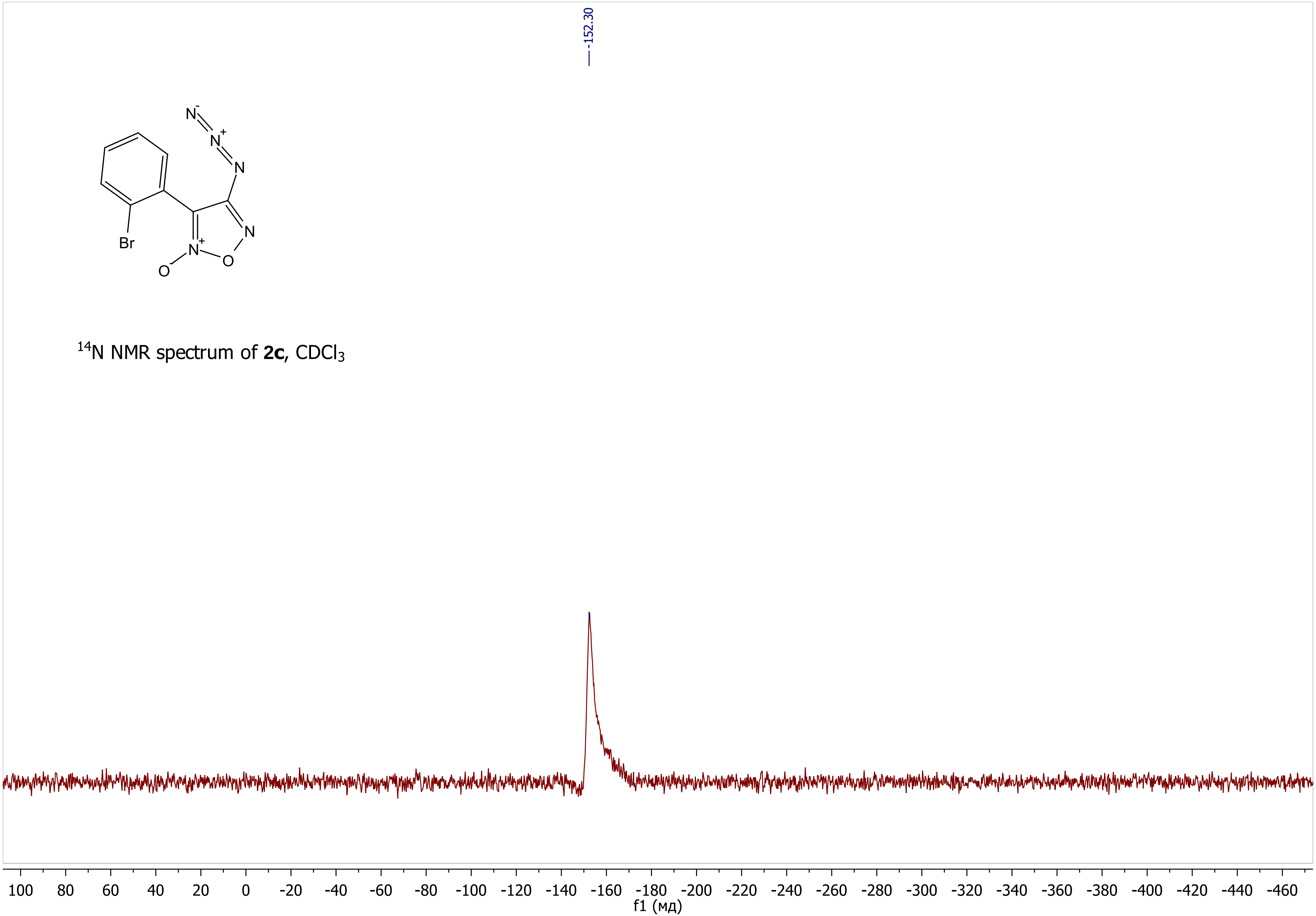

^{14}N NMR spectrum of **2c**, $CDCl_3$

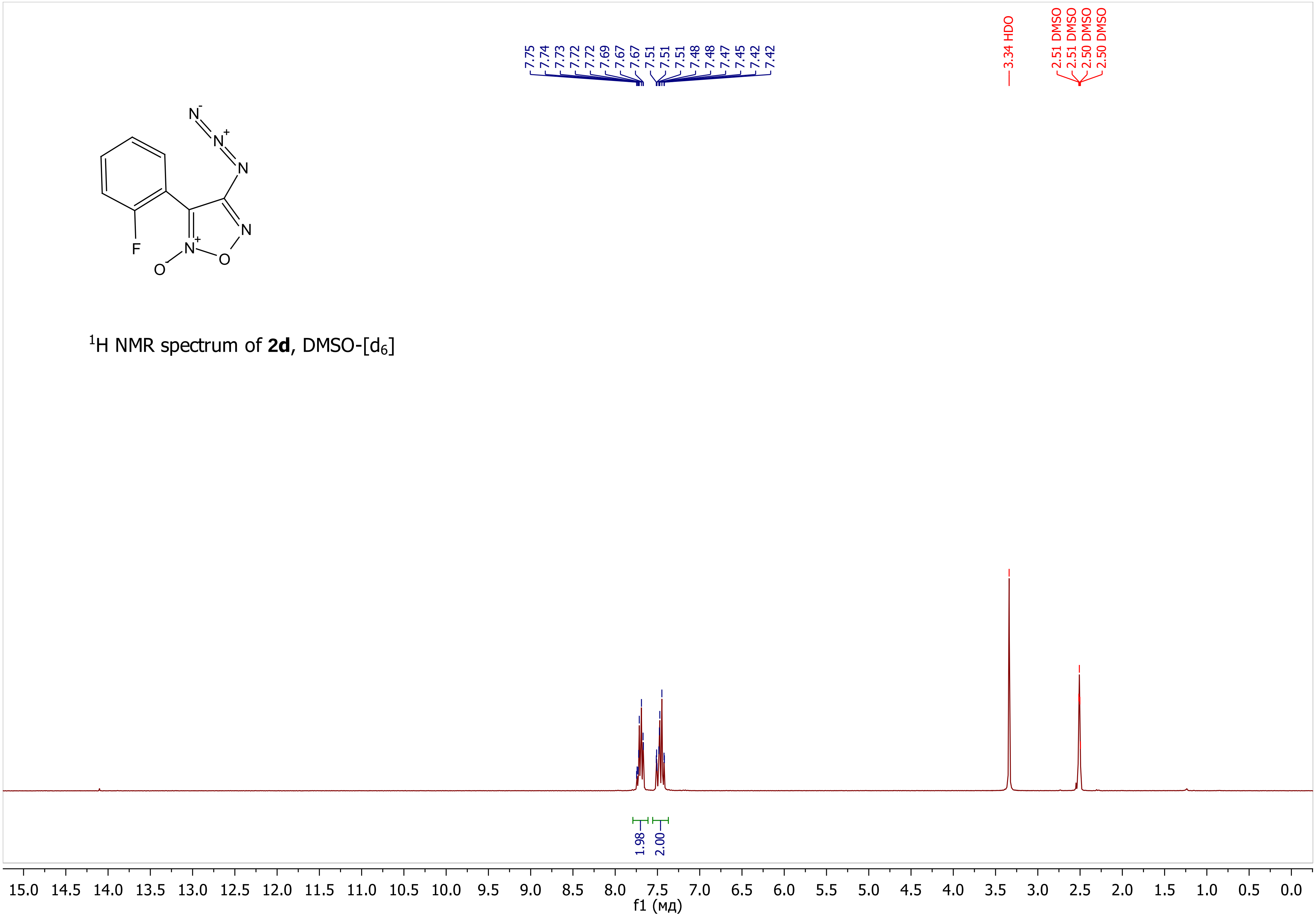

^{1}H NMR spectrum of **2d**, DMSO-[d$_6$]

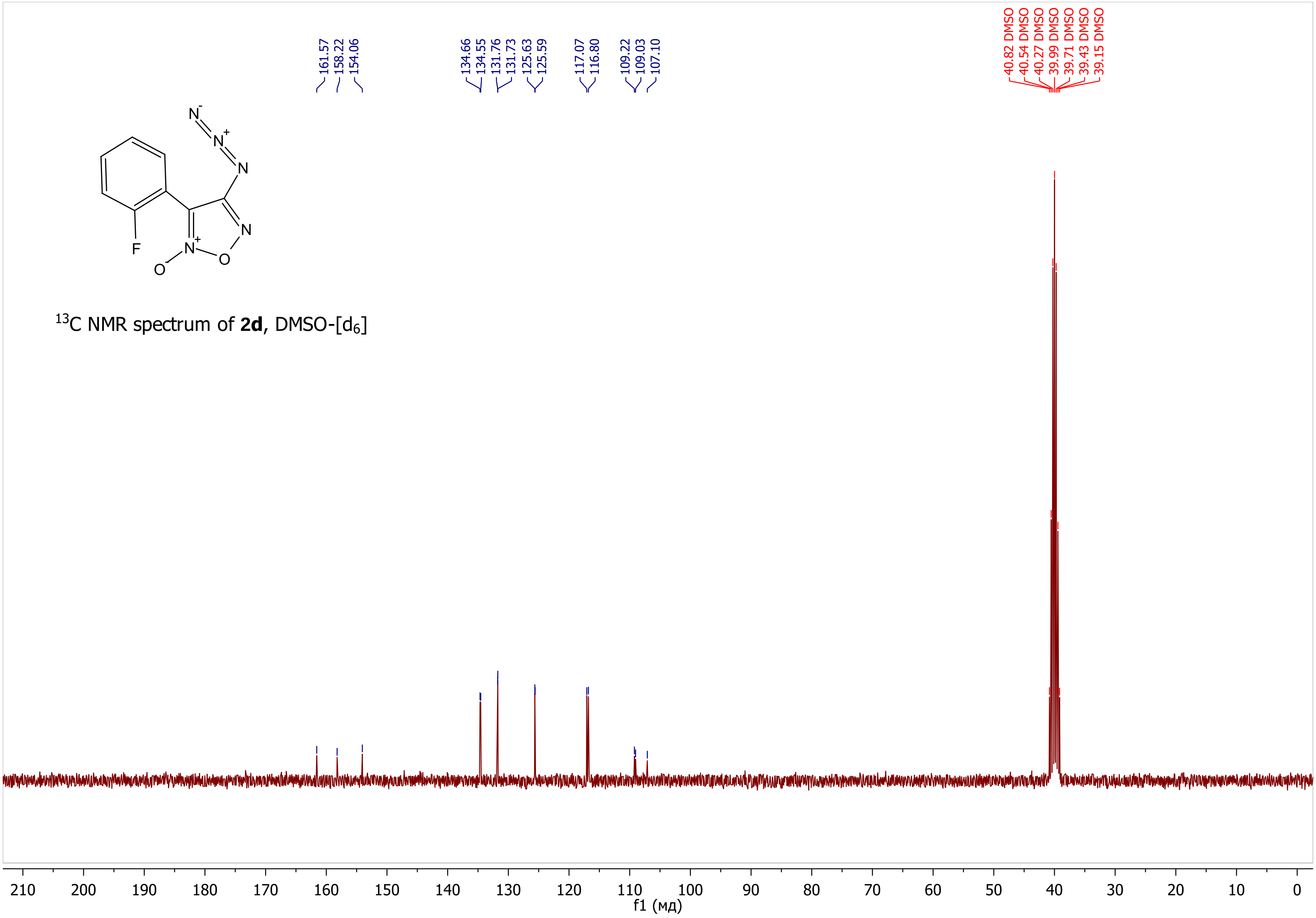

^{13}C NMR spectrum of **2d**, DMSO-[d_6]

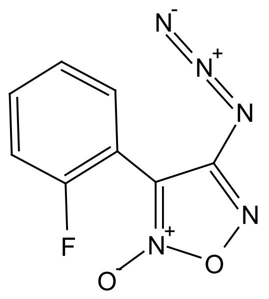

^{14}N NMR spectrum of **2d**, DMSO-[d$_6$]

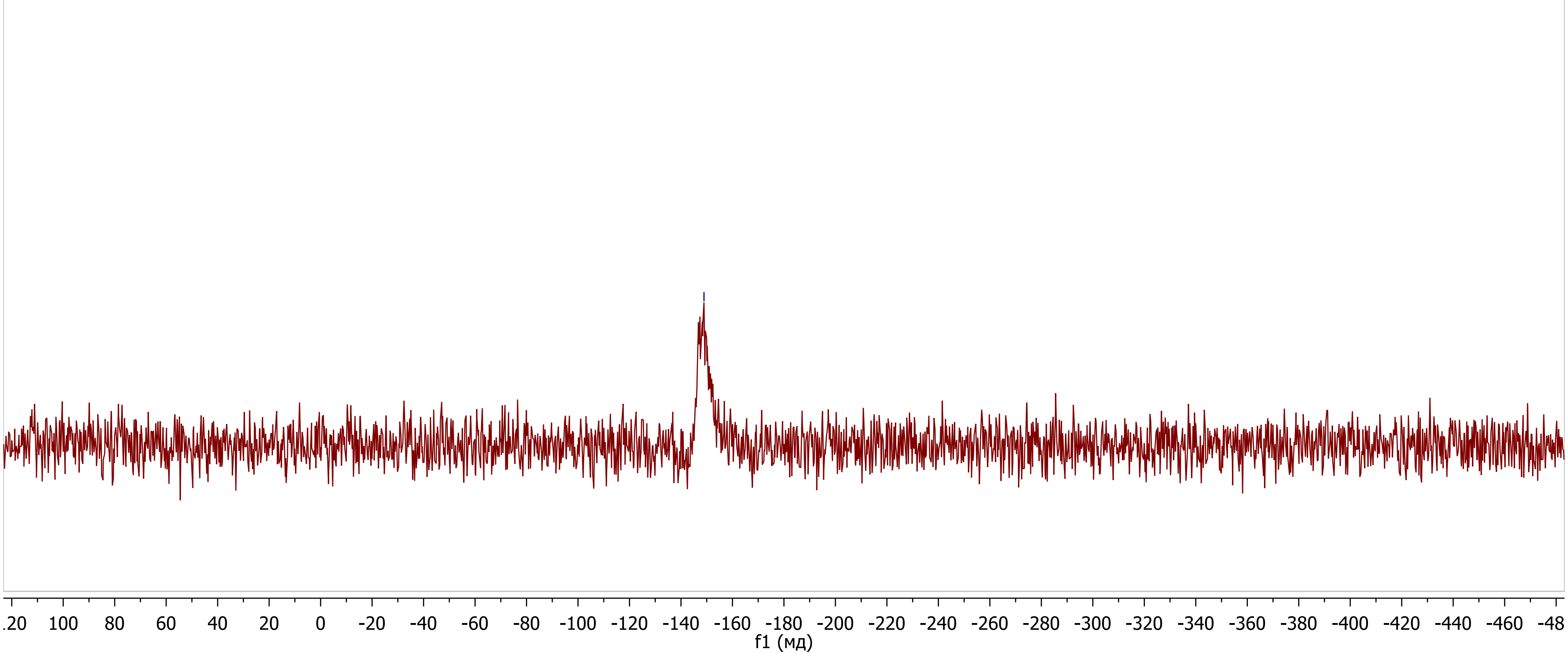

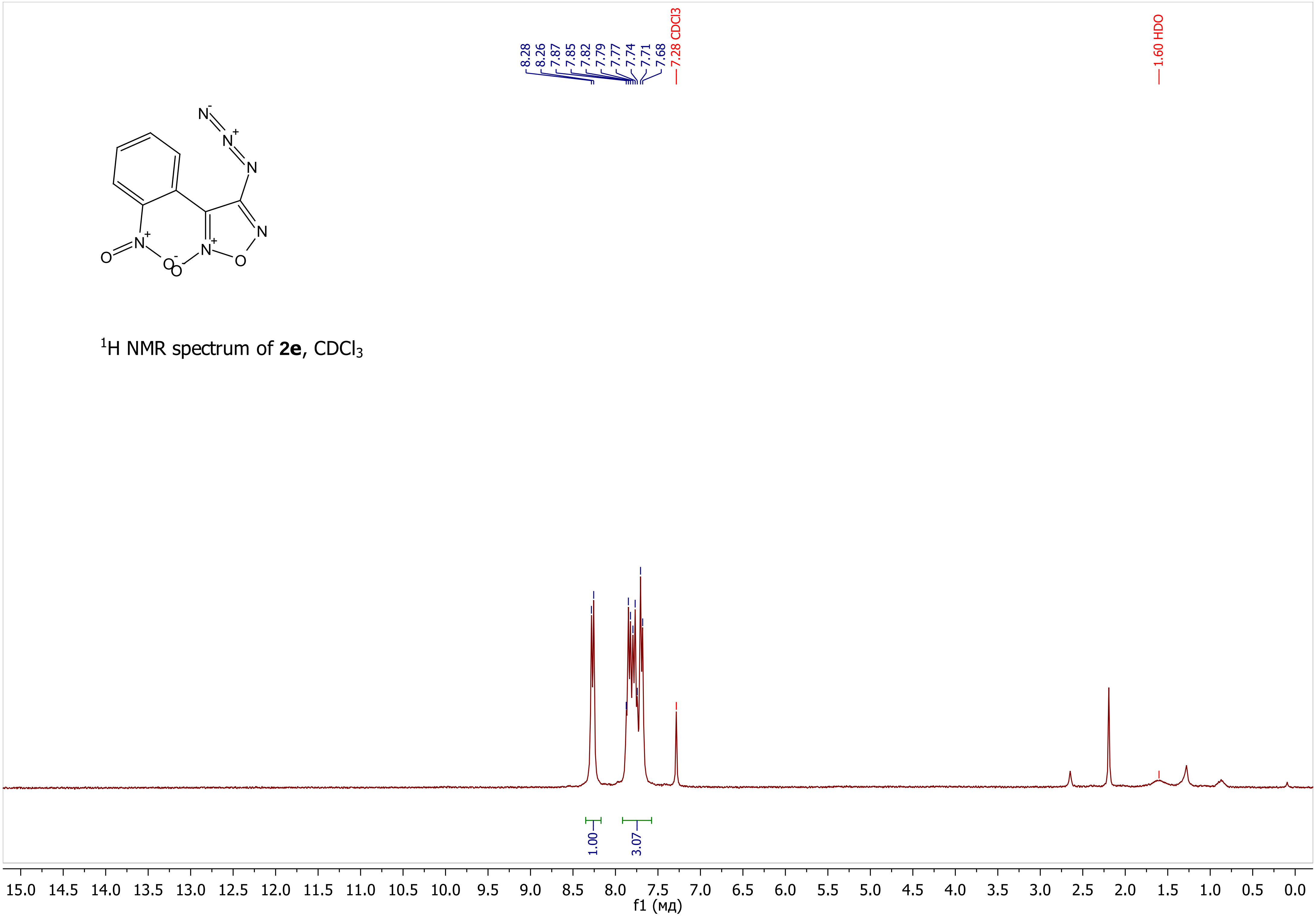

8.28
8.26
7.87
7.85
7.82
7.79
7.77
7.74
7.71
7.68
7.28 CDCl3
1.60 HDO
^{1}H NMR spectrum of **2e**, $CDCl_3$
1.00
3.07
15.0 14.5 14.0 13.5 13.0 12.5 12.0 11.5 11.0 10.5 10.0 9.5 9.0 8.5 8.0 7.5 7.0 6.5 6.0 5.5 5.0 4.5 4.0 3.5 3.0 2.5 2.0 1.5 1.0 0.5 0.0
f1 (мд)

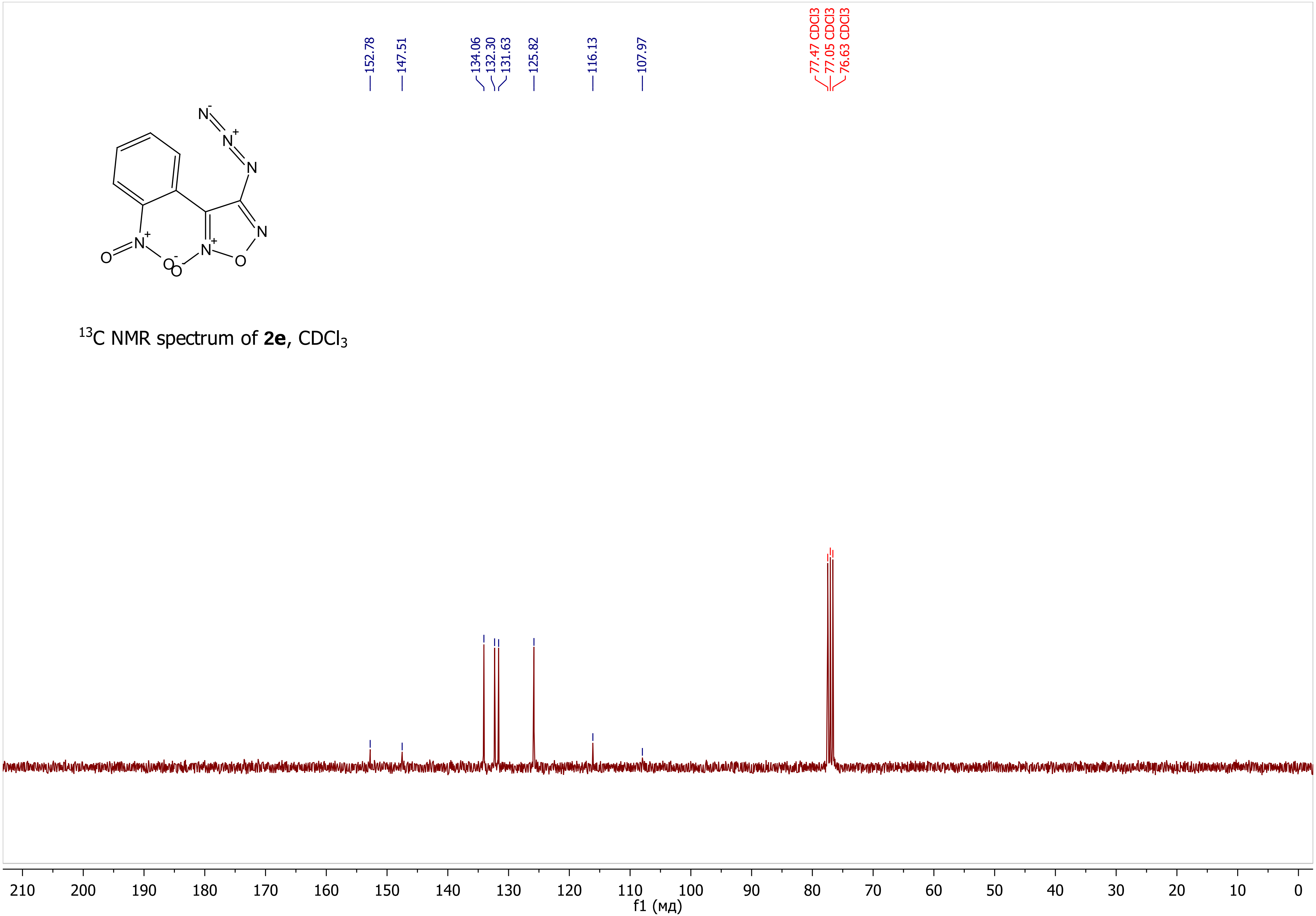

^{13}C NMR spectrum of **2e**, $CDCl_3$

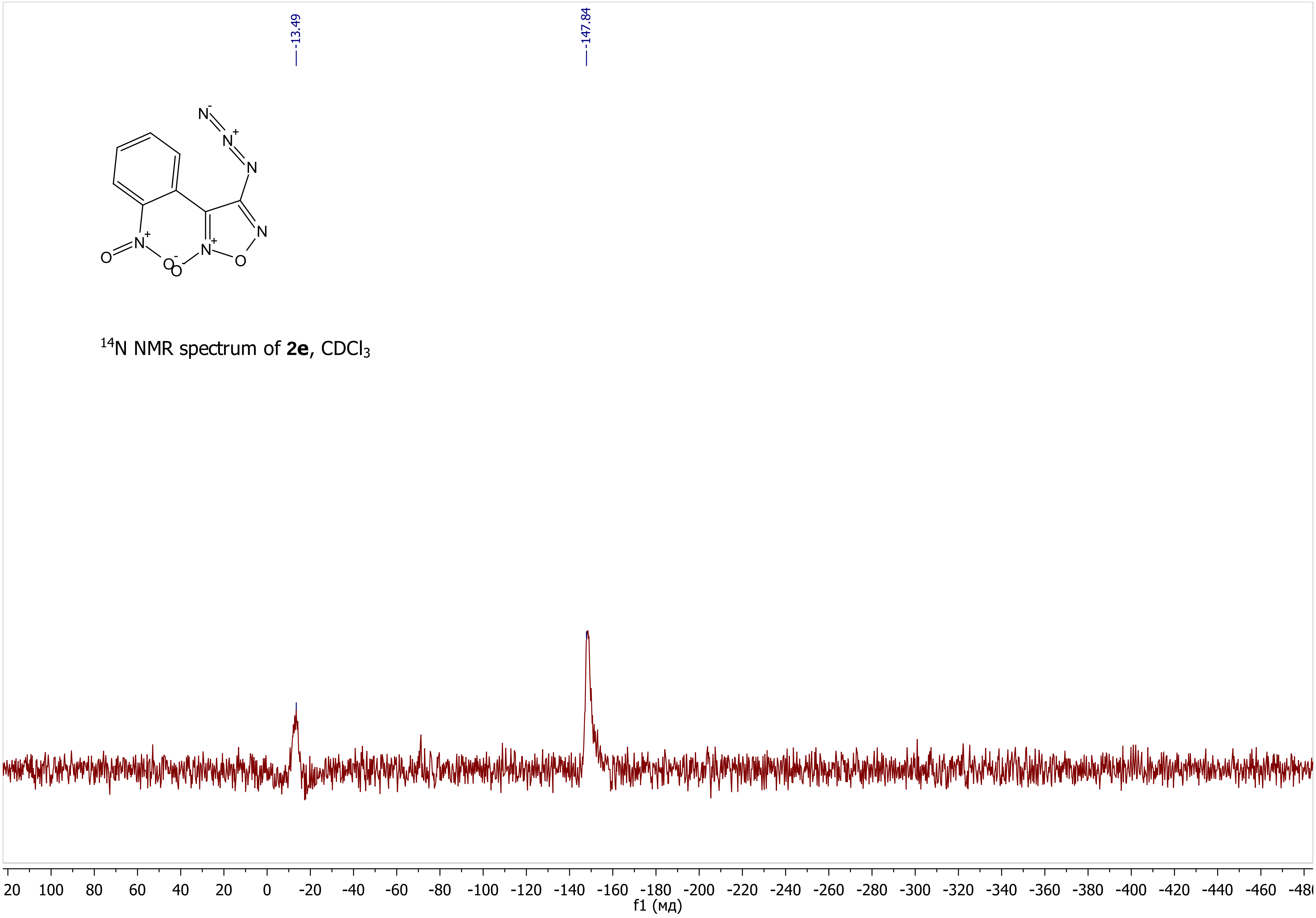

^{14}N NMR spectrum of **2e**, $CDCl_3$

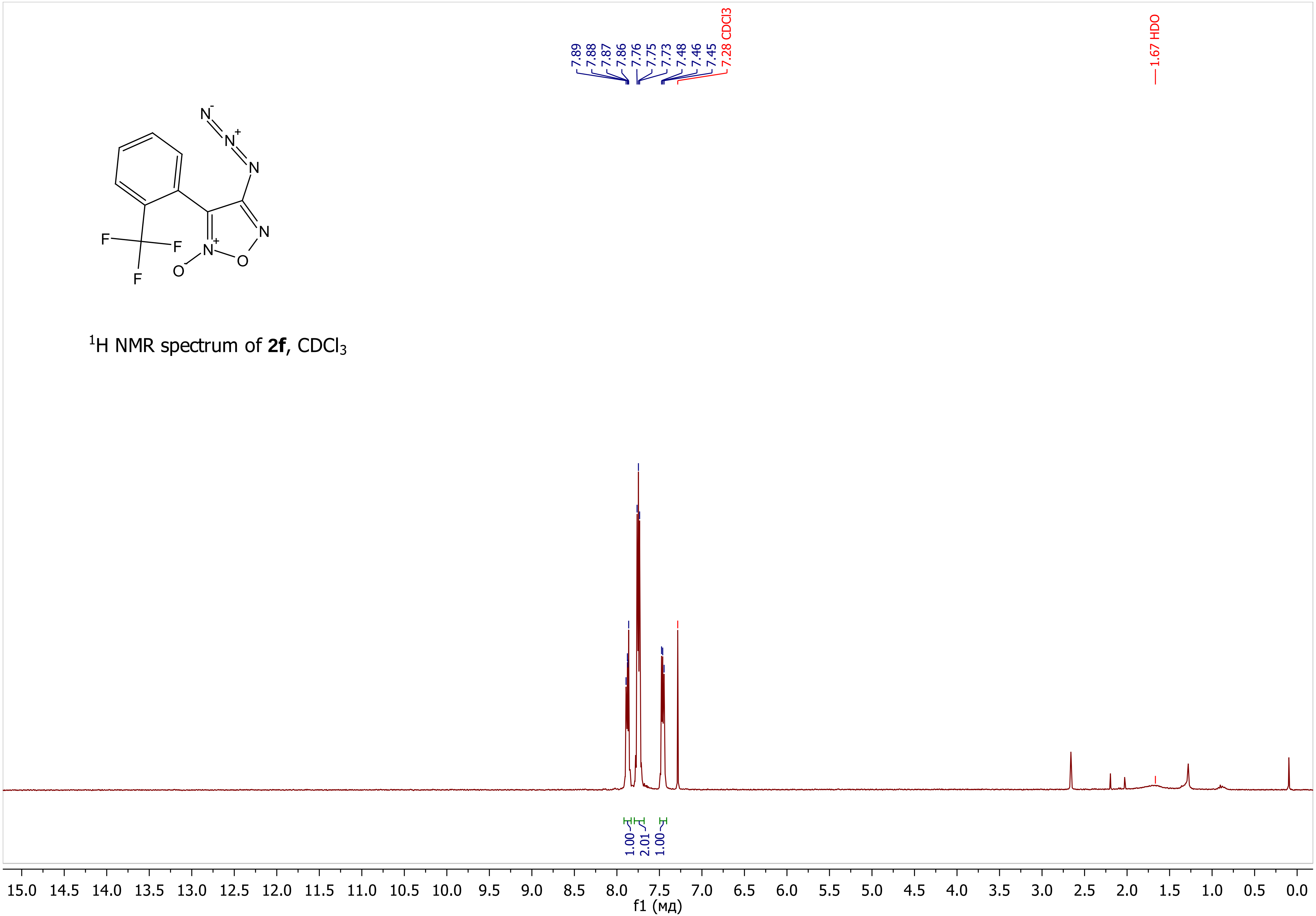

^{1}H NMR spectrum of **2f**, $CDCl_3$

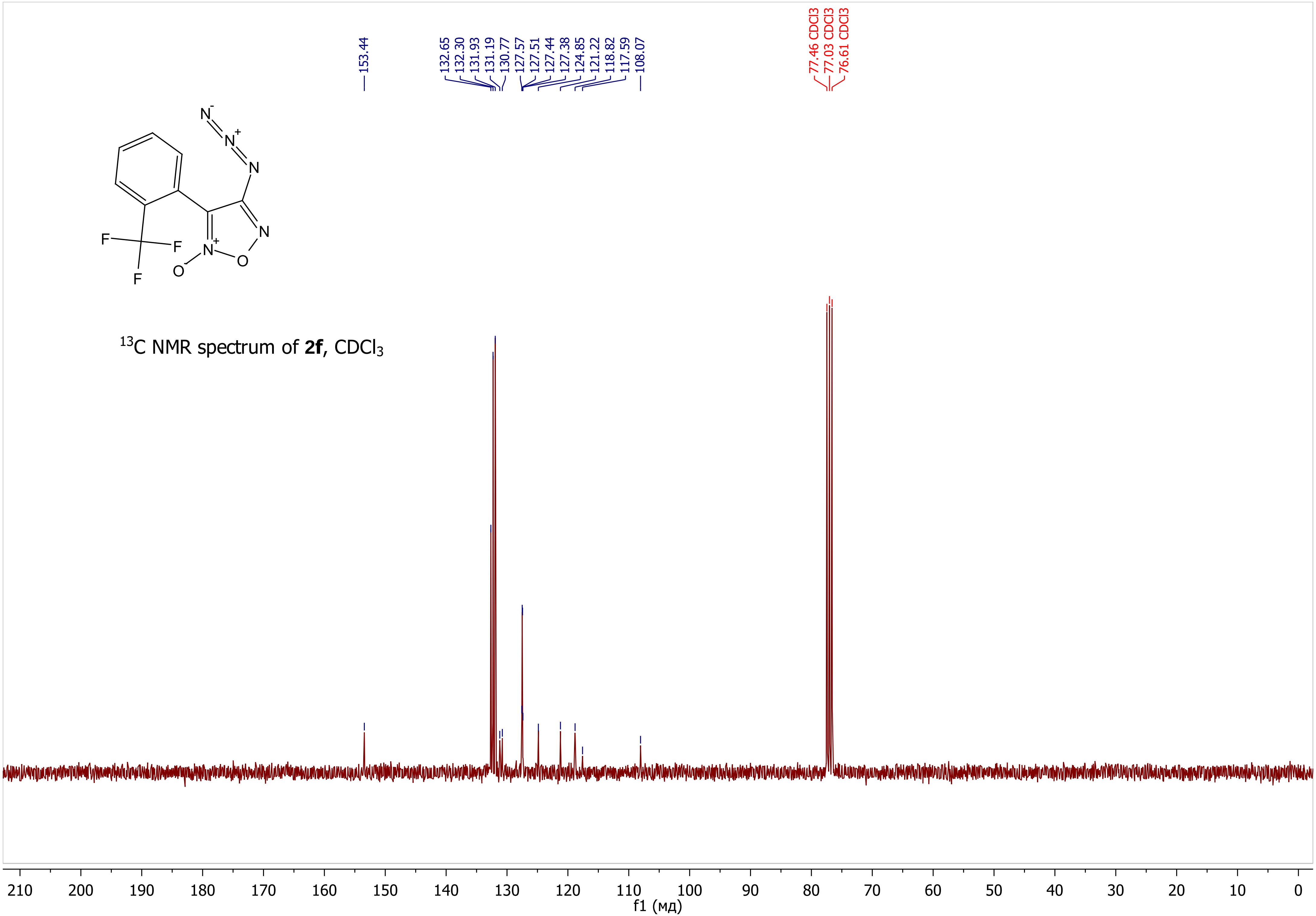

^{13}C NMR spectrum of **2f**, $CDCl_3$

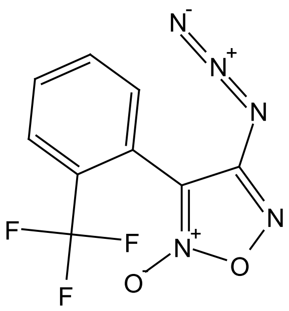

^{14}N NMR spectrum of **2f**, $CDCl_3$

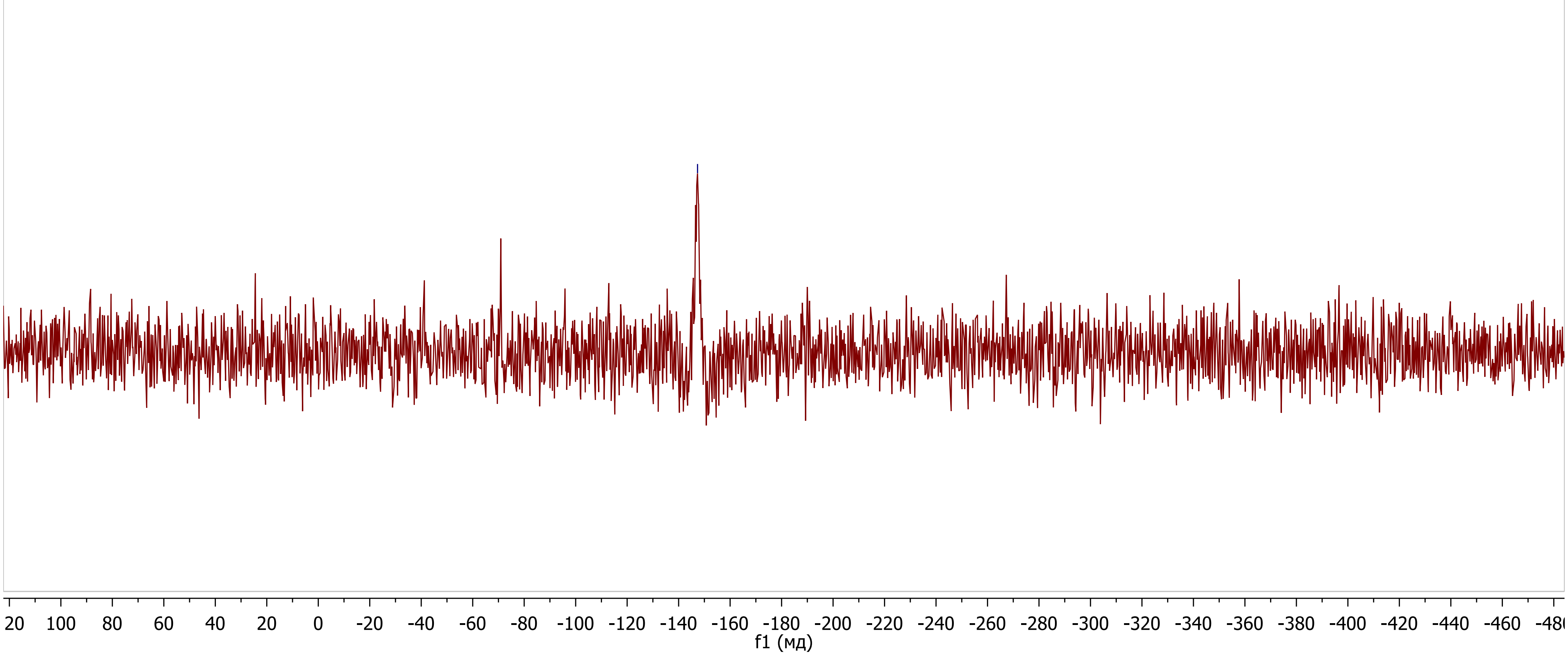

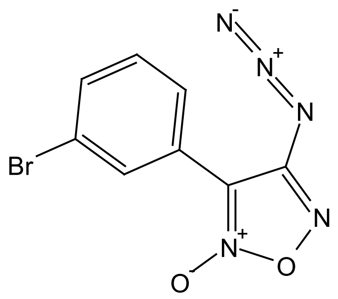

^{1}H NMR spectrum of **2g**, $CDCl_3$

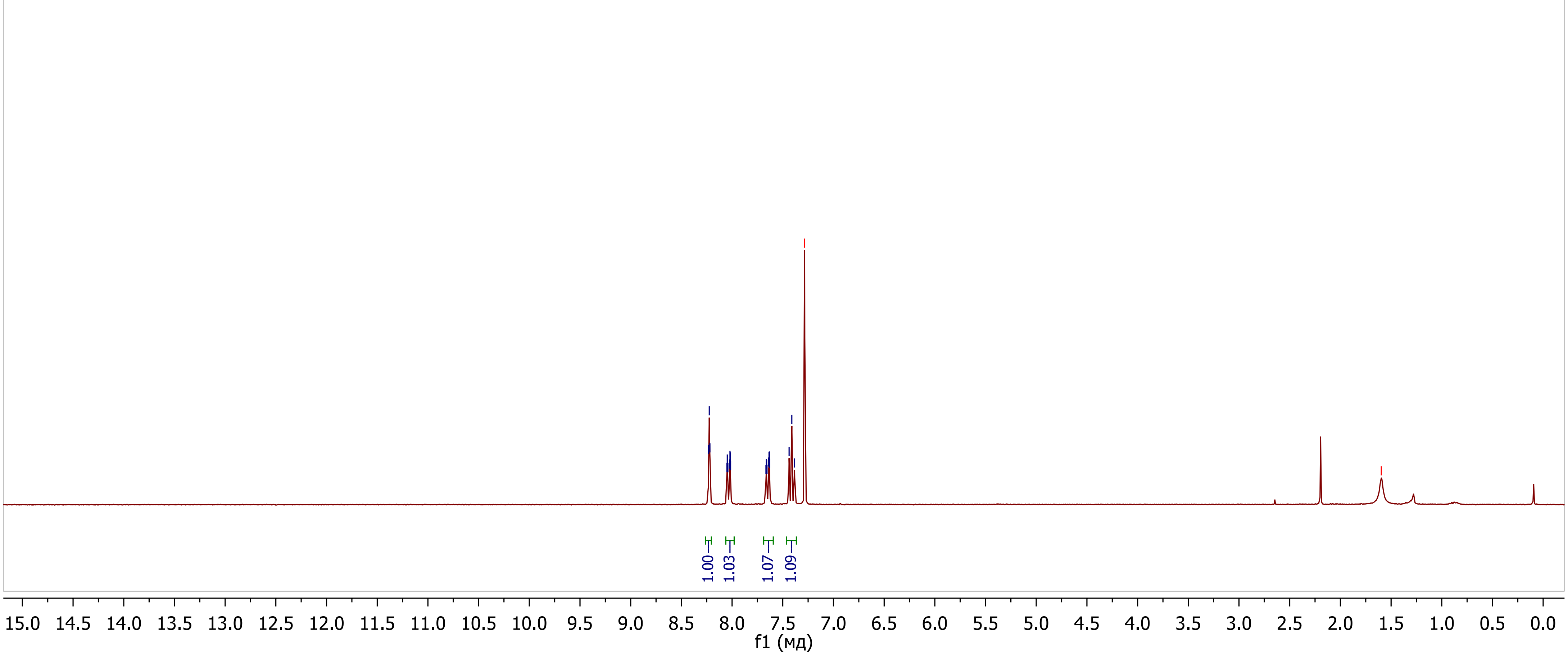

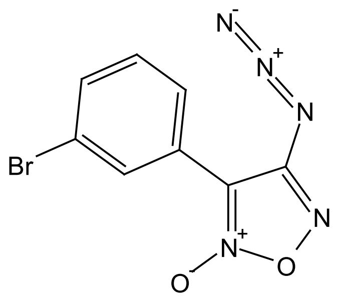

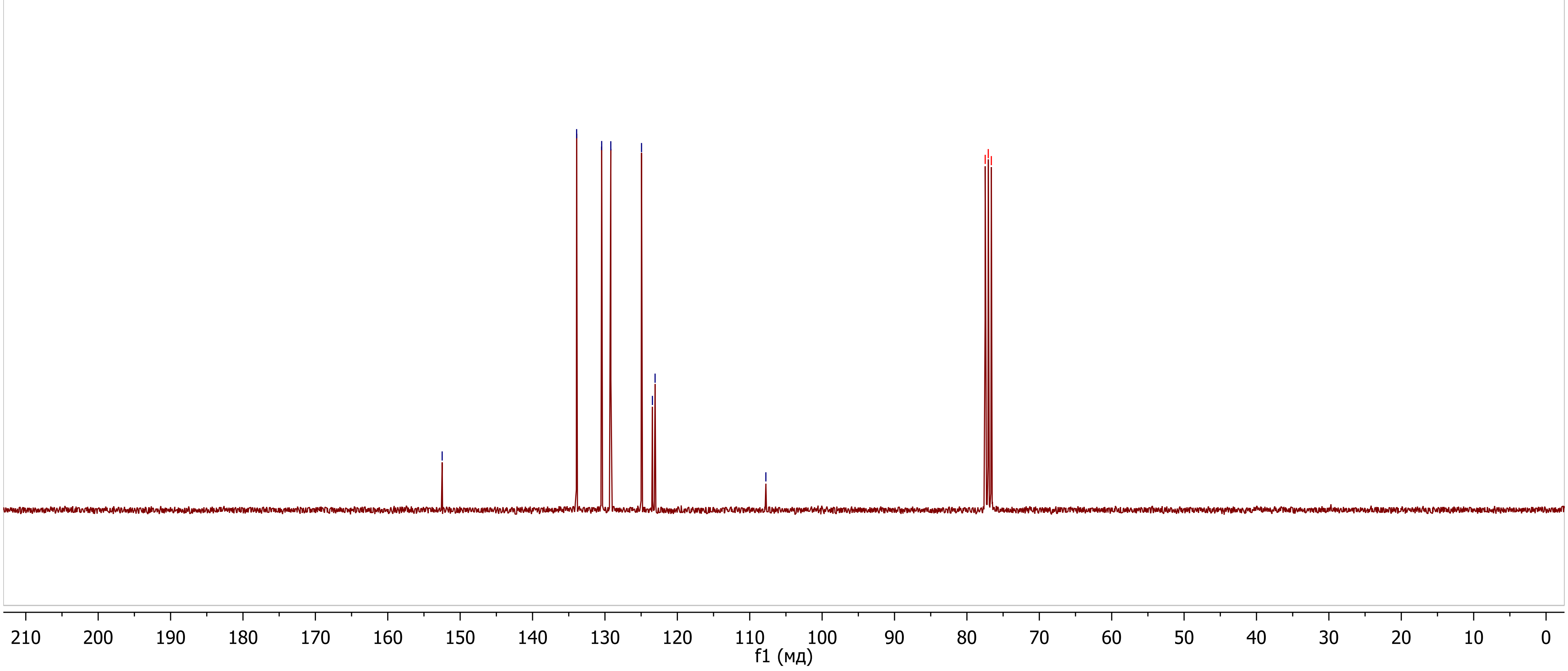

^{13}C NMR spectrum of **2g**, $CDCl_3$

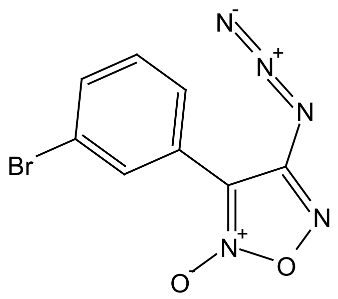

^{14}N NMR spectrum of **2g**, $CDCl_3$

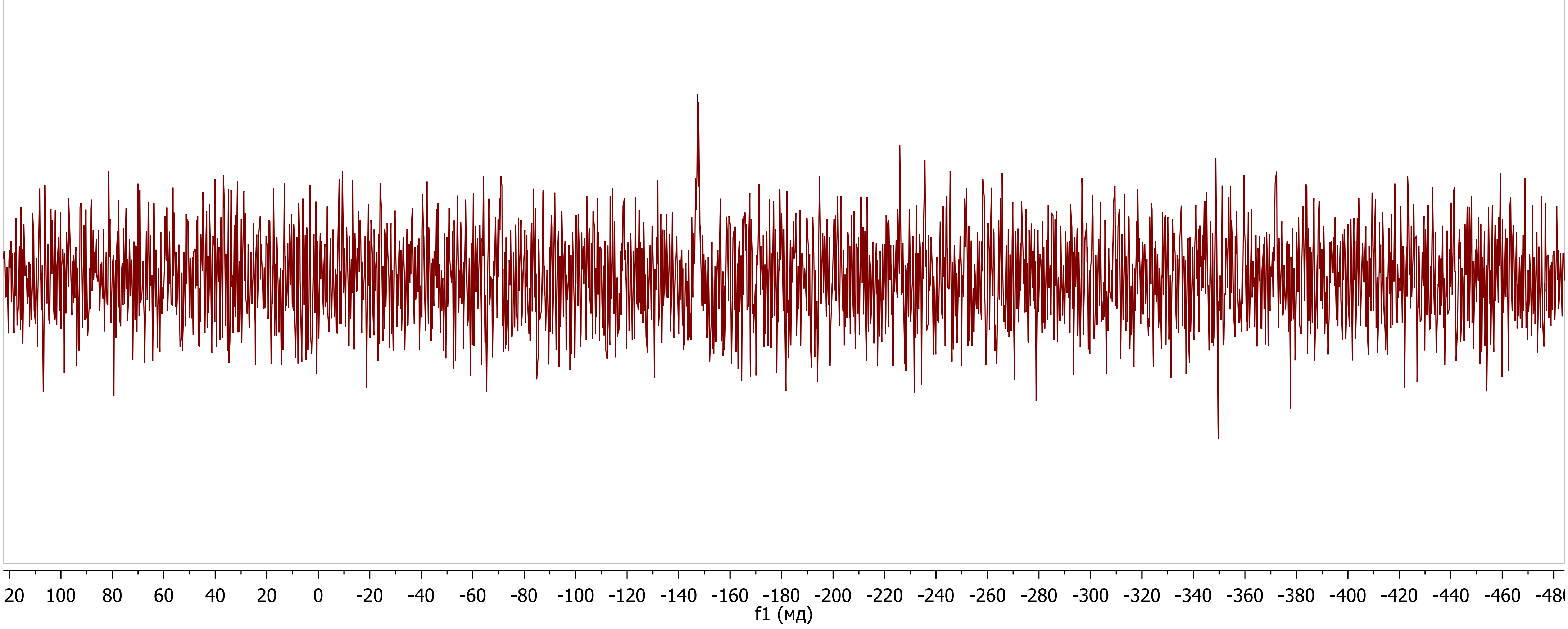

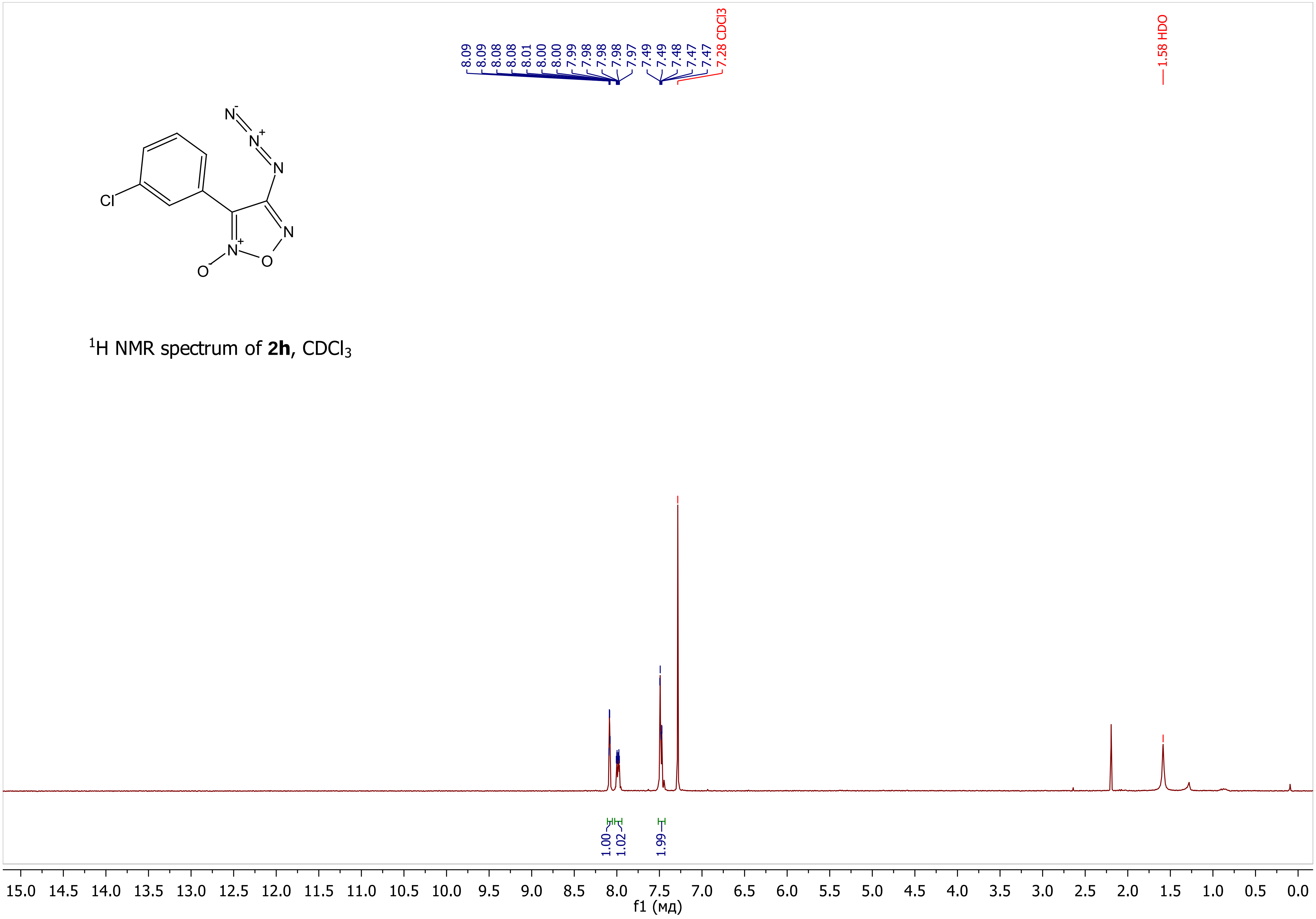

^{1}H NMR spectrum of **2h**, $CDCl_3$

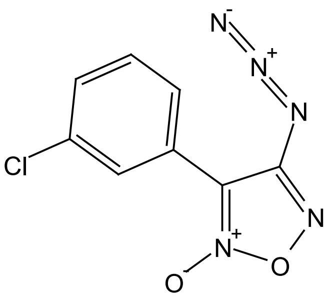

^{13}C NMR spectrum of **2h**, $CDCl_3$

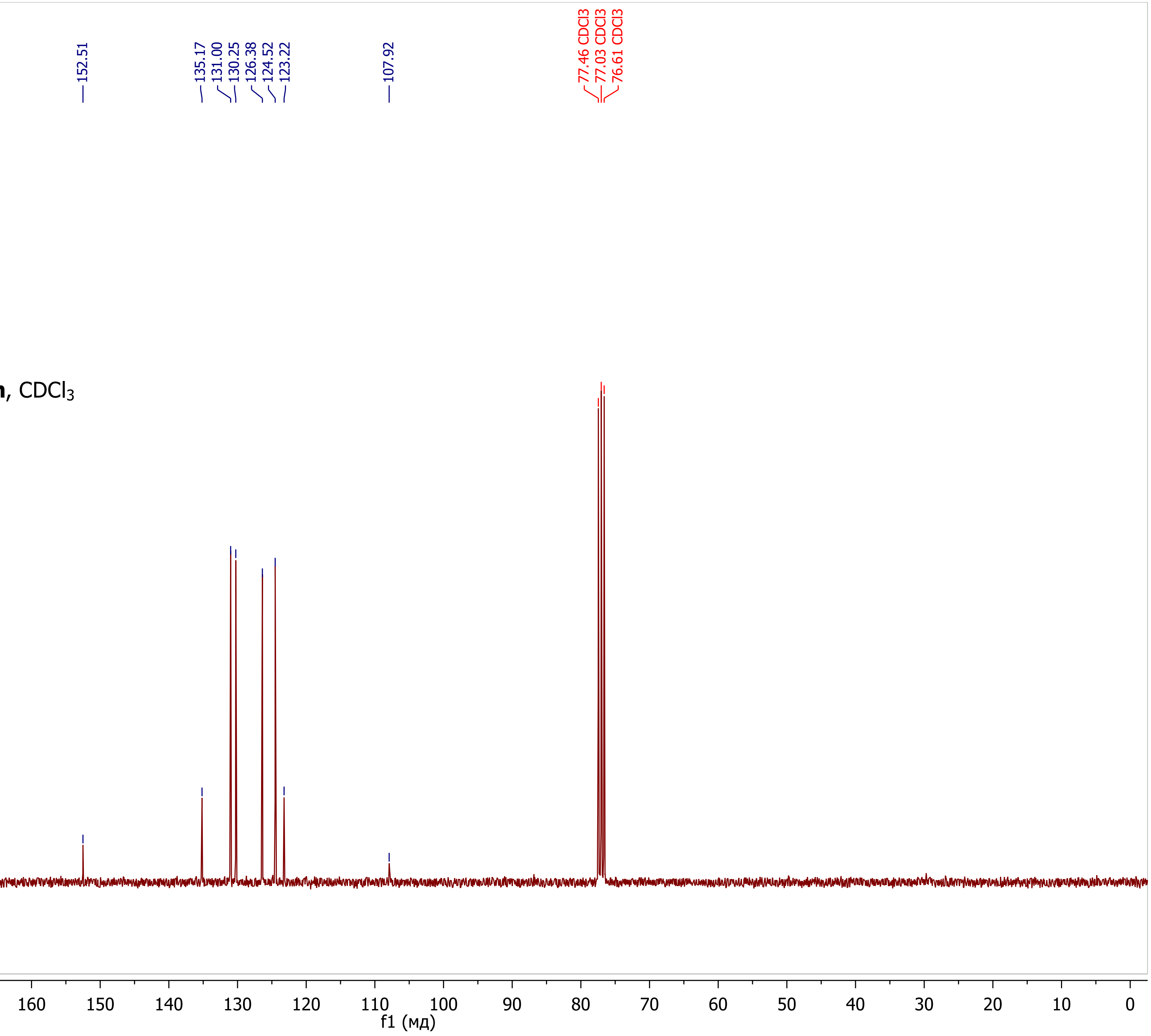

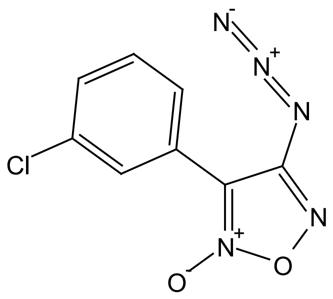

^{14}N NMR spectrum of **2h**, $CDCl_3$

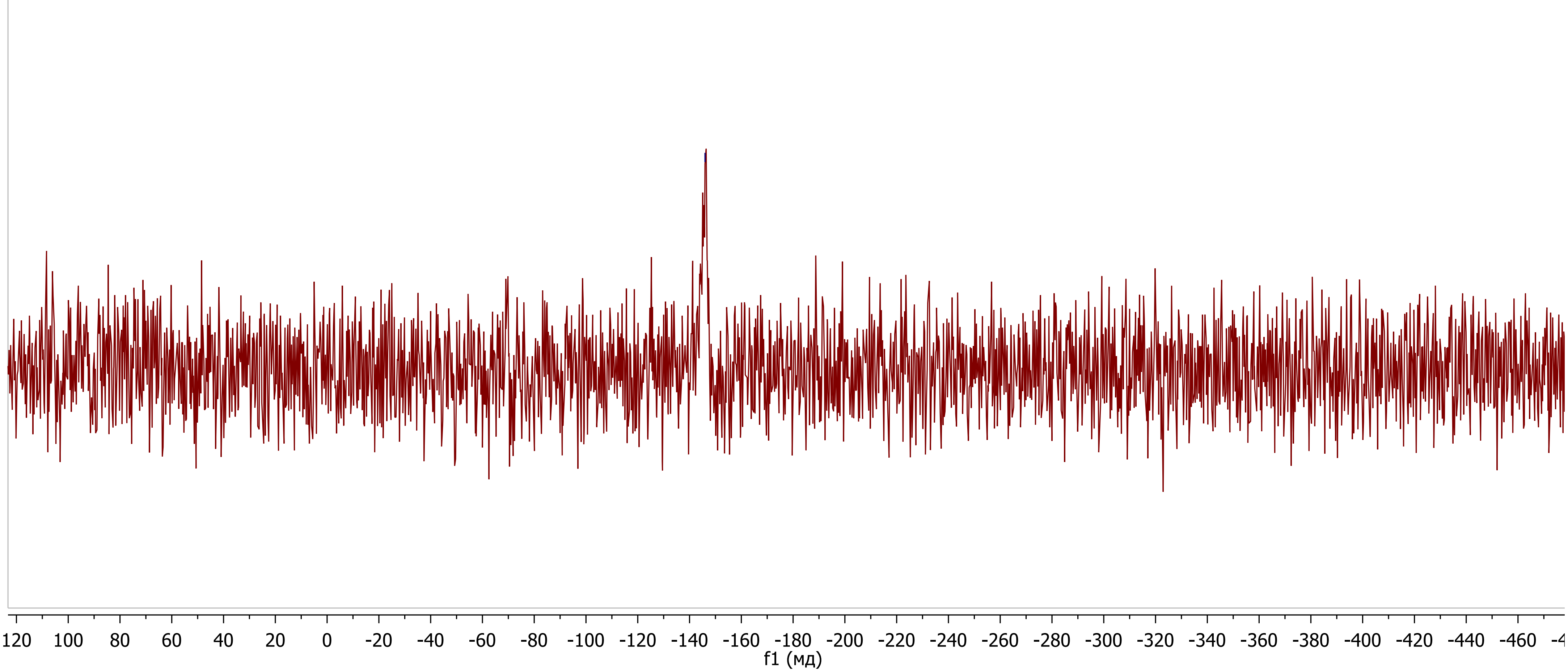

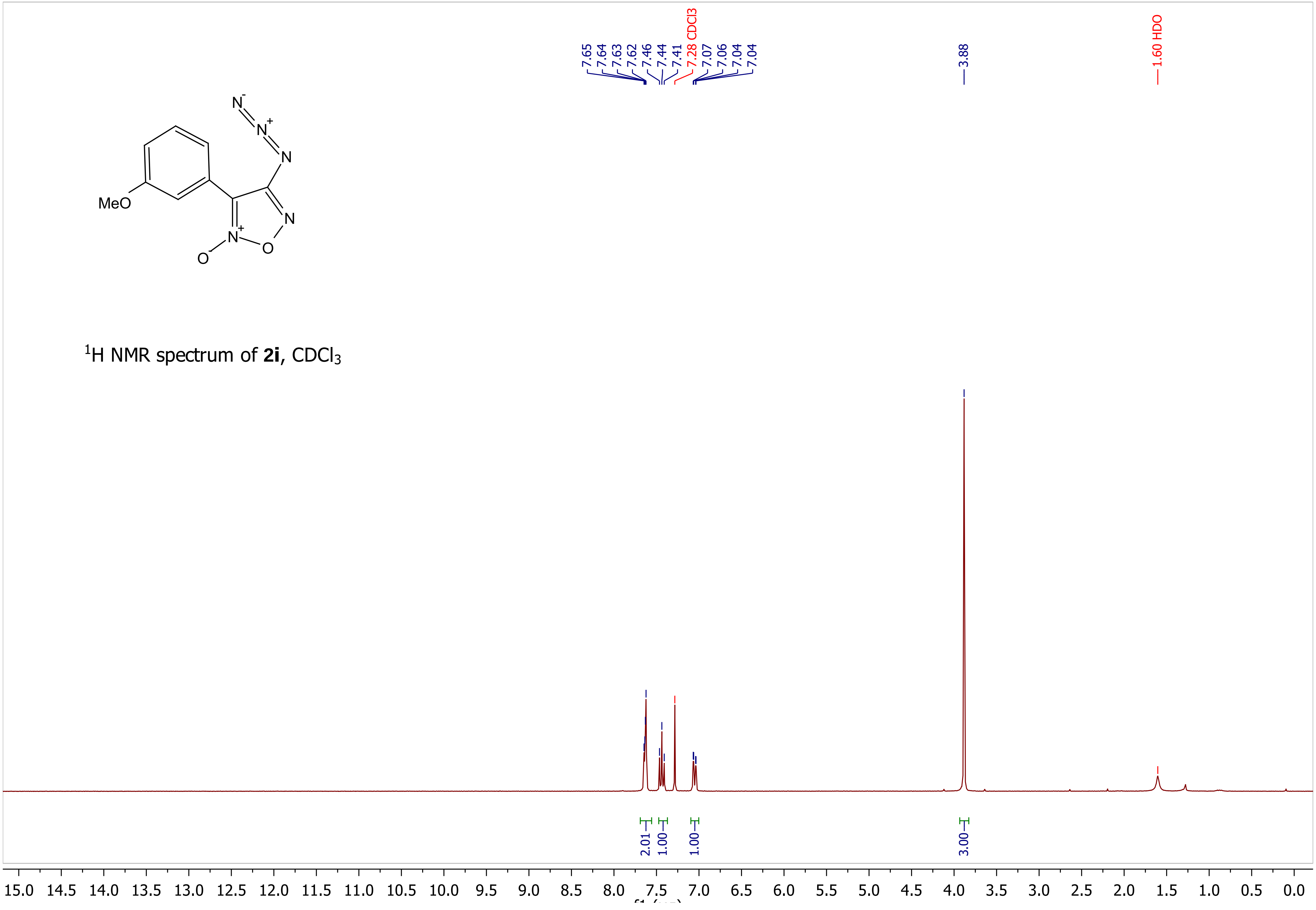

^{1}H NMR spectrum of **2i**, $CDCl_3$

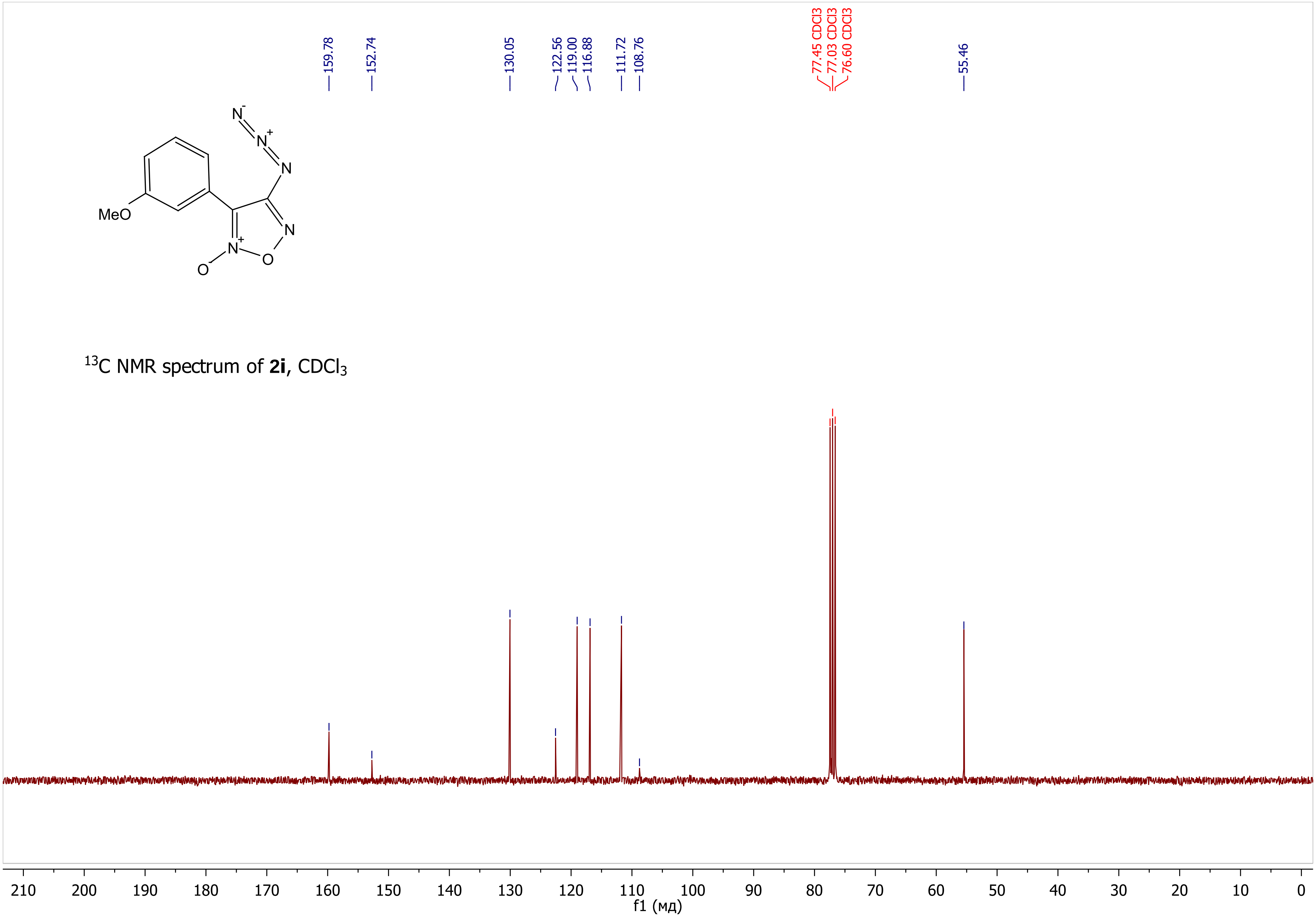

^{13}C NMR spectrum of **2i**, $CDCl_3$

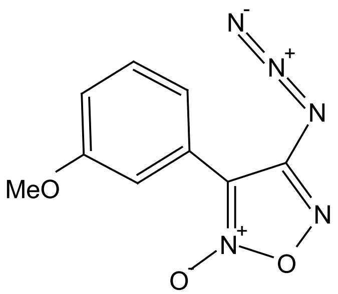

^{14}N NMR spectrum of **2i**, $CDCl_3$

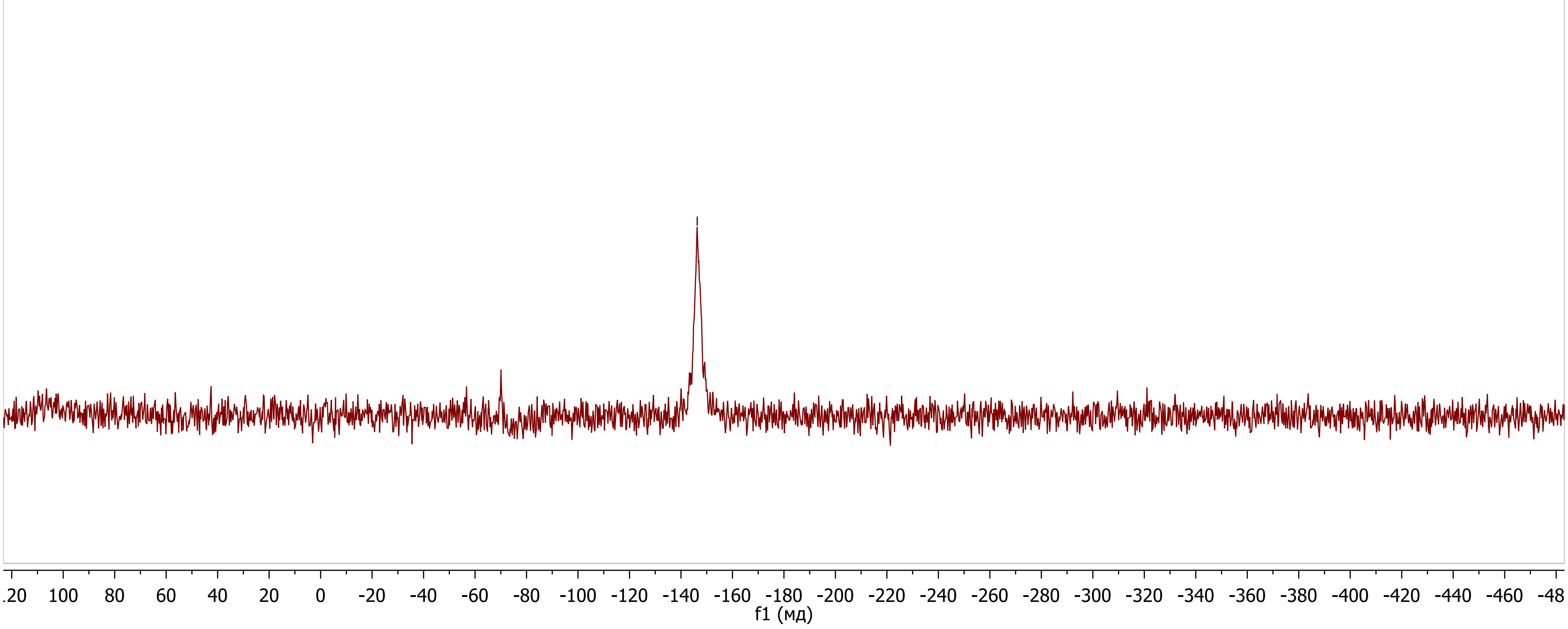

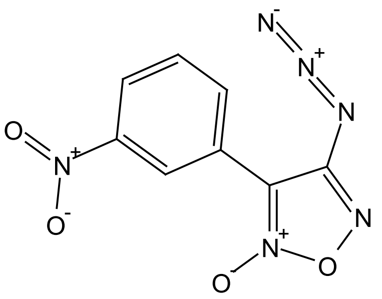

¹H NMR spectrum of **2j**, $CDCl_3$

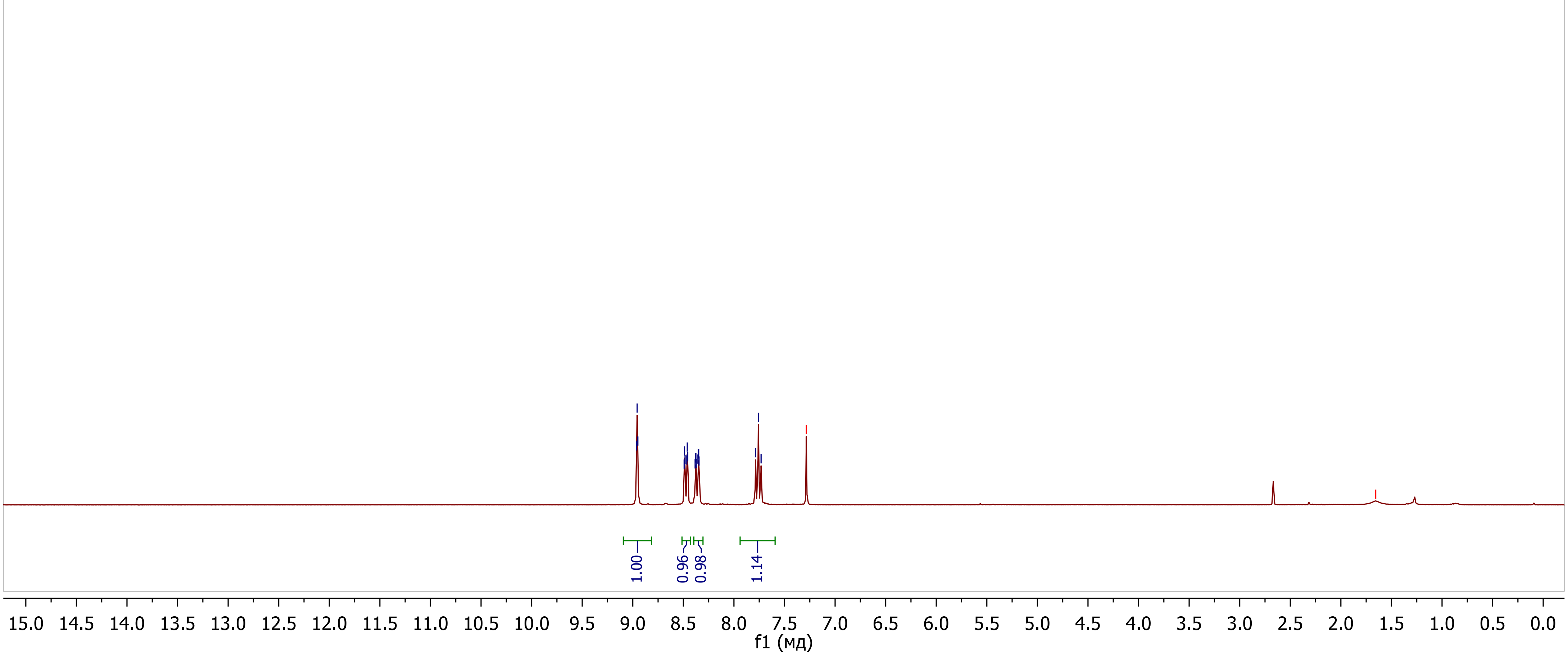

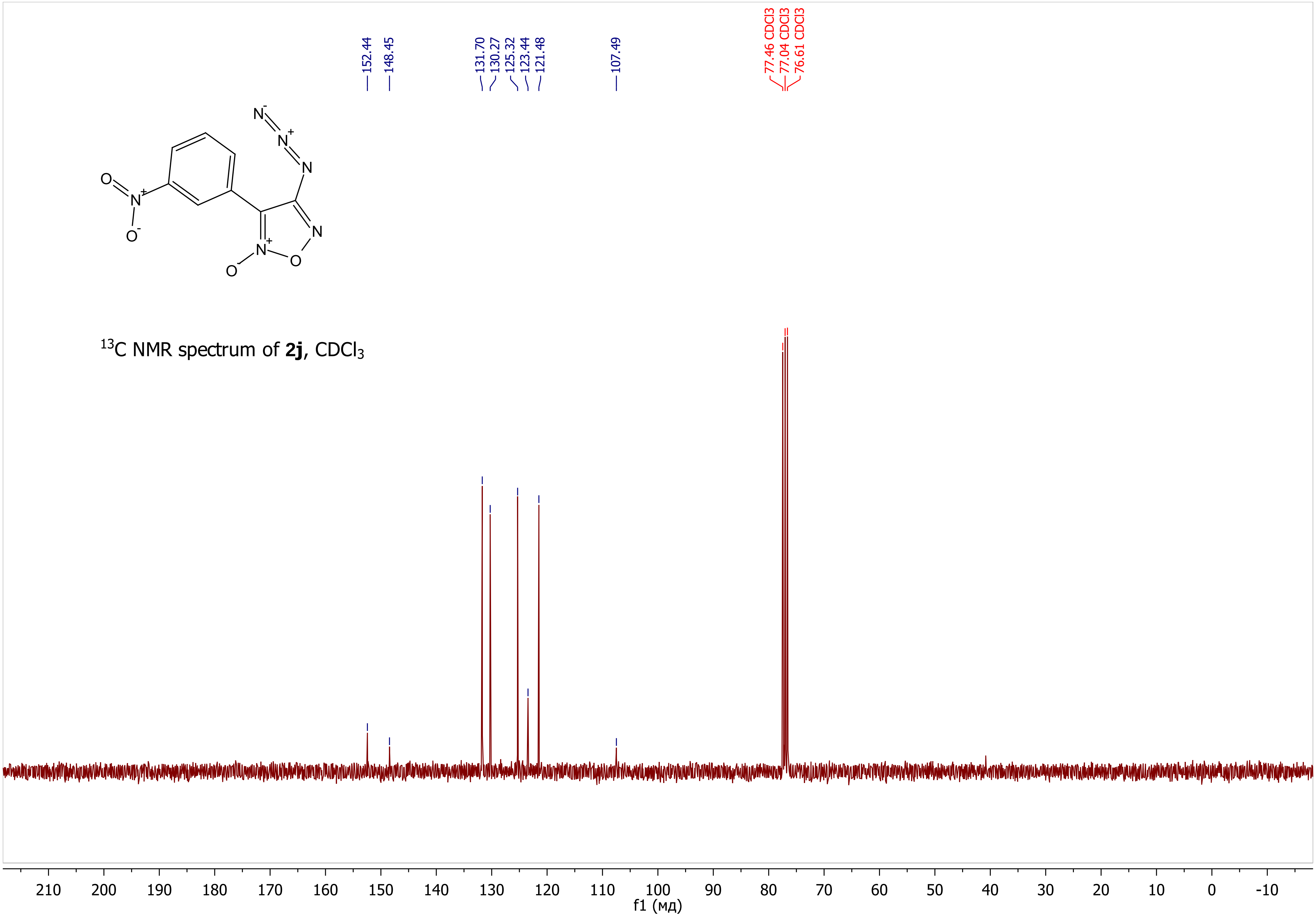

^{13}C NMR spectrum of **2j**, $CDCl_3$

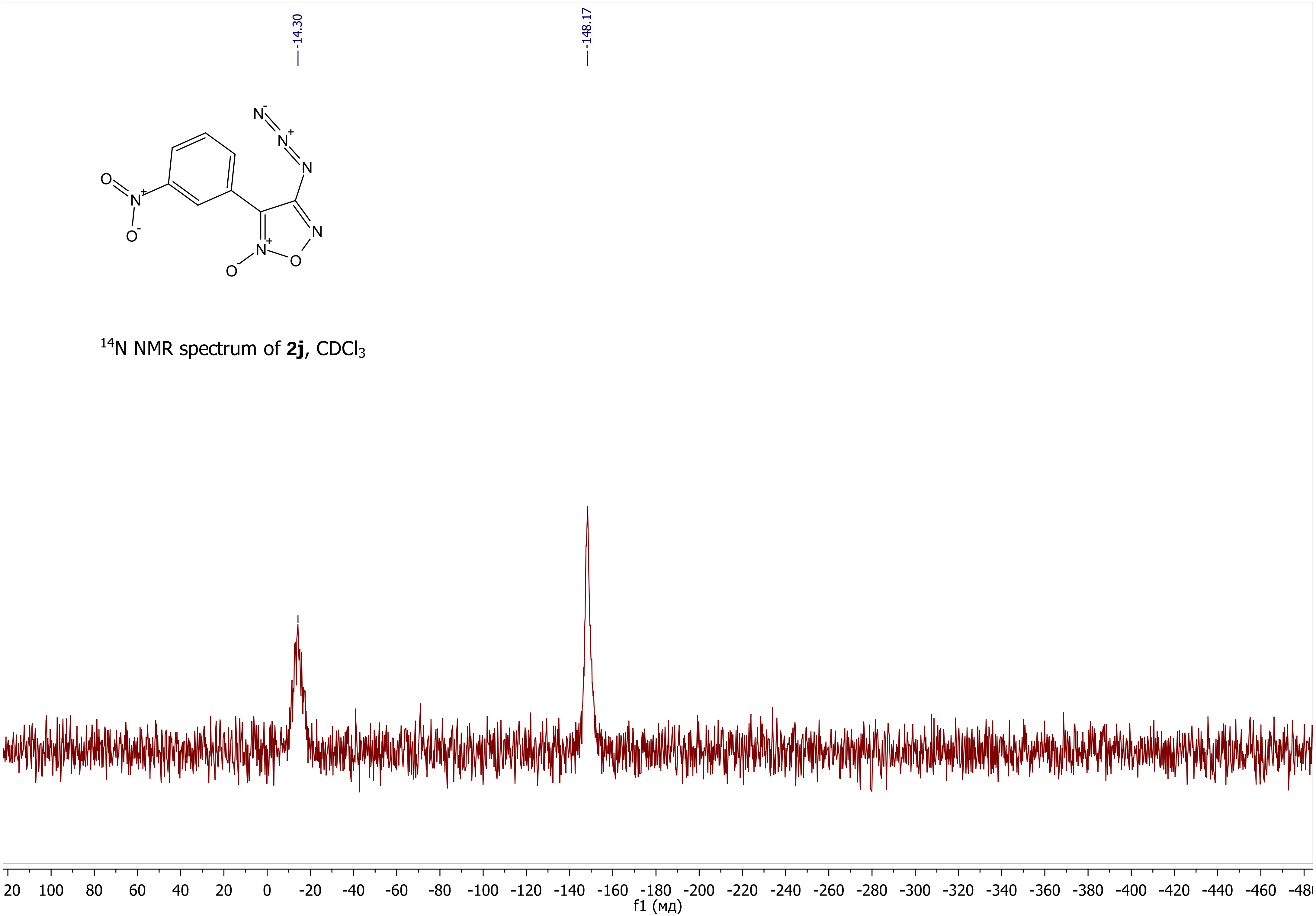

^{14}N NMR spectrum of **2j**, $CDCl_3$

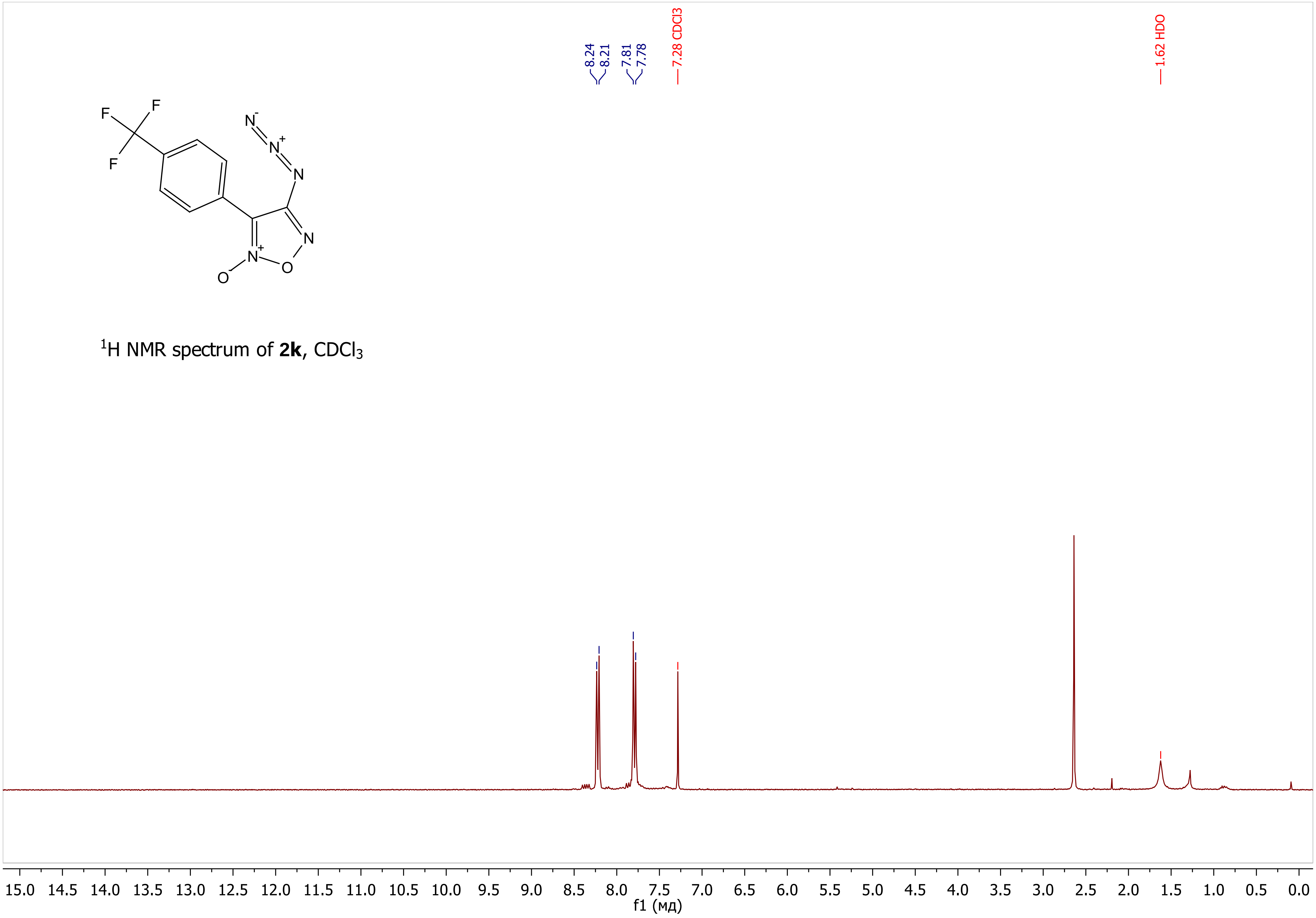

^{1}H NMR spectrum of **2k**, $CDCl_3$

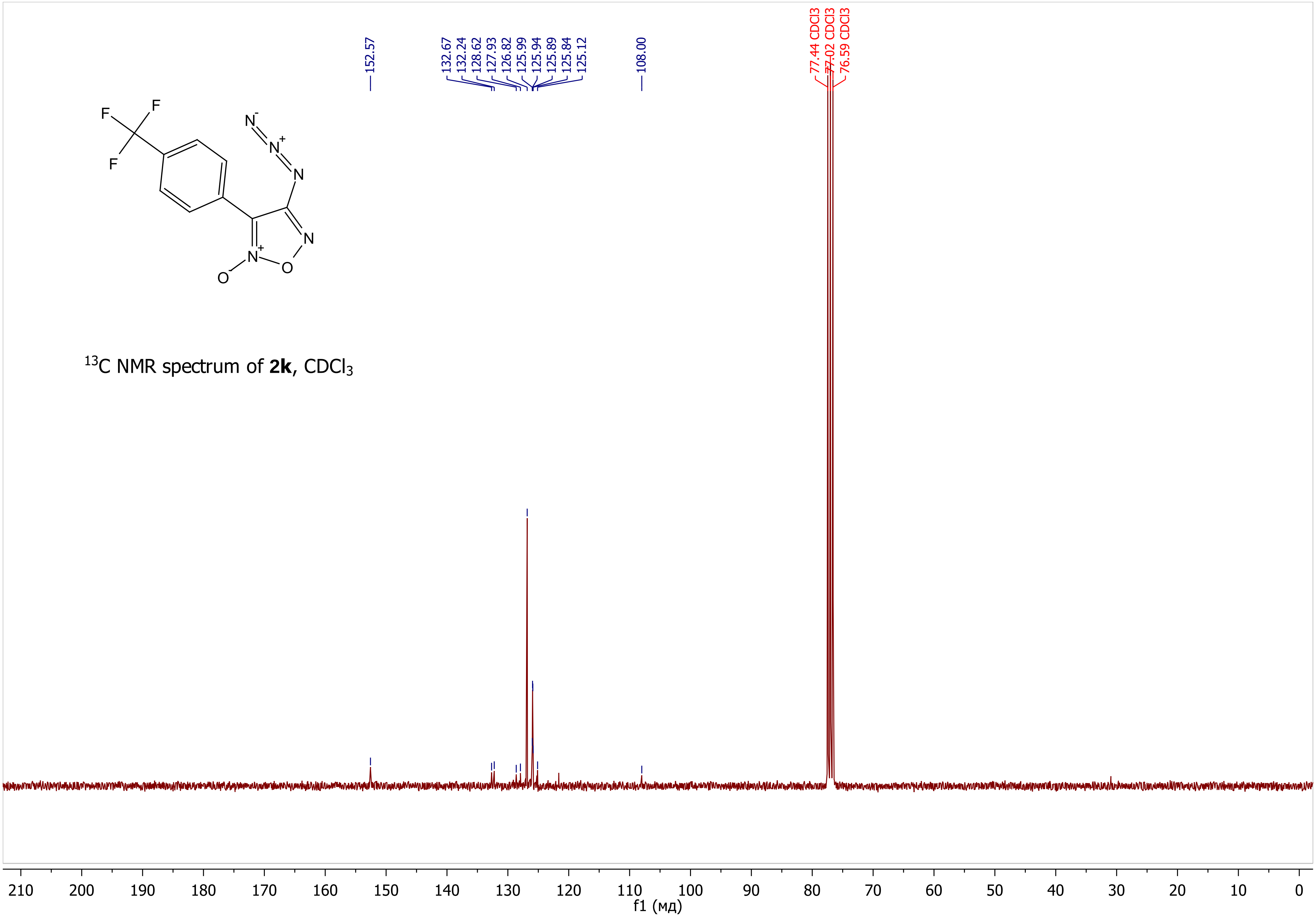

^{13}C NMR spectrum of **2k**, $CDCl_3$

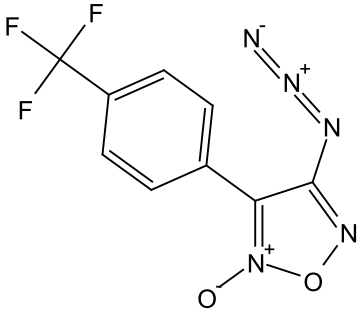

^{14}N NMR spectrum of **2k**, $CDCl_3$

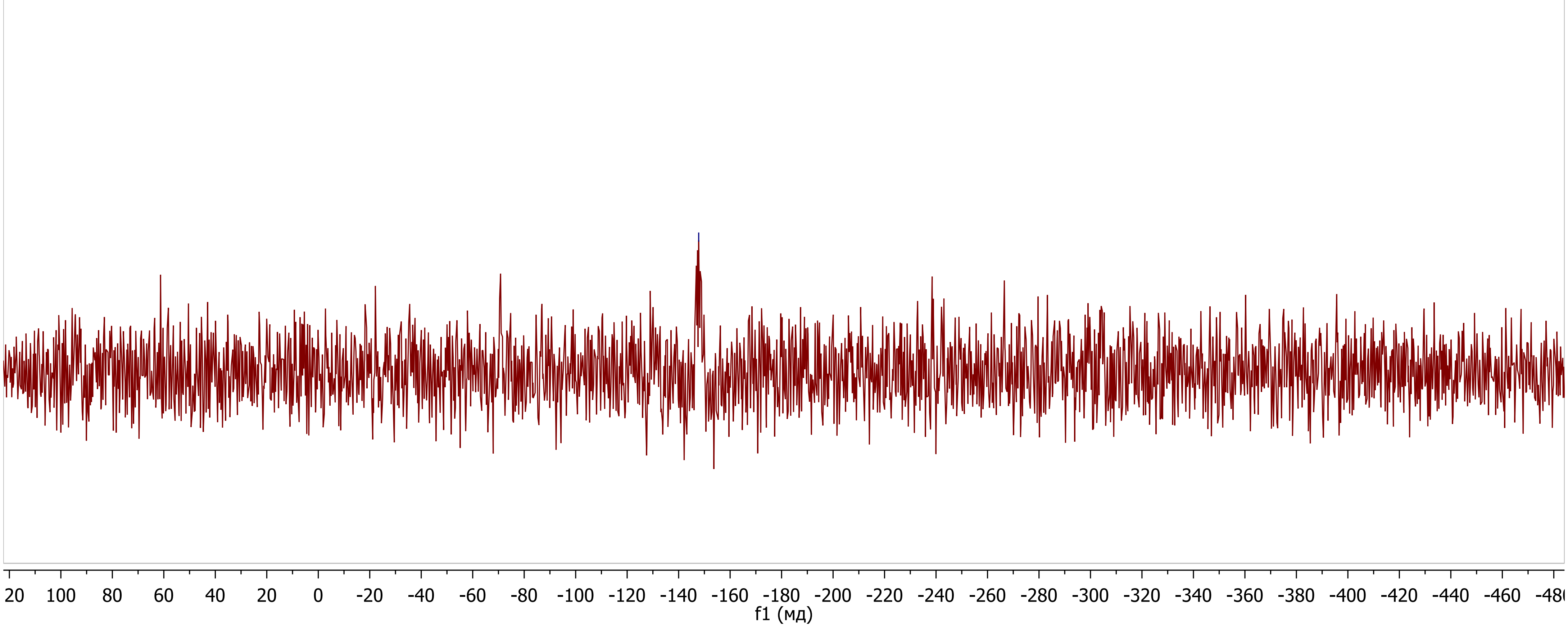

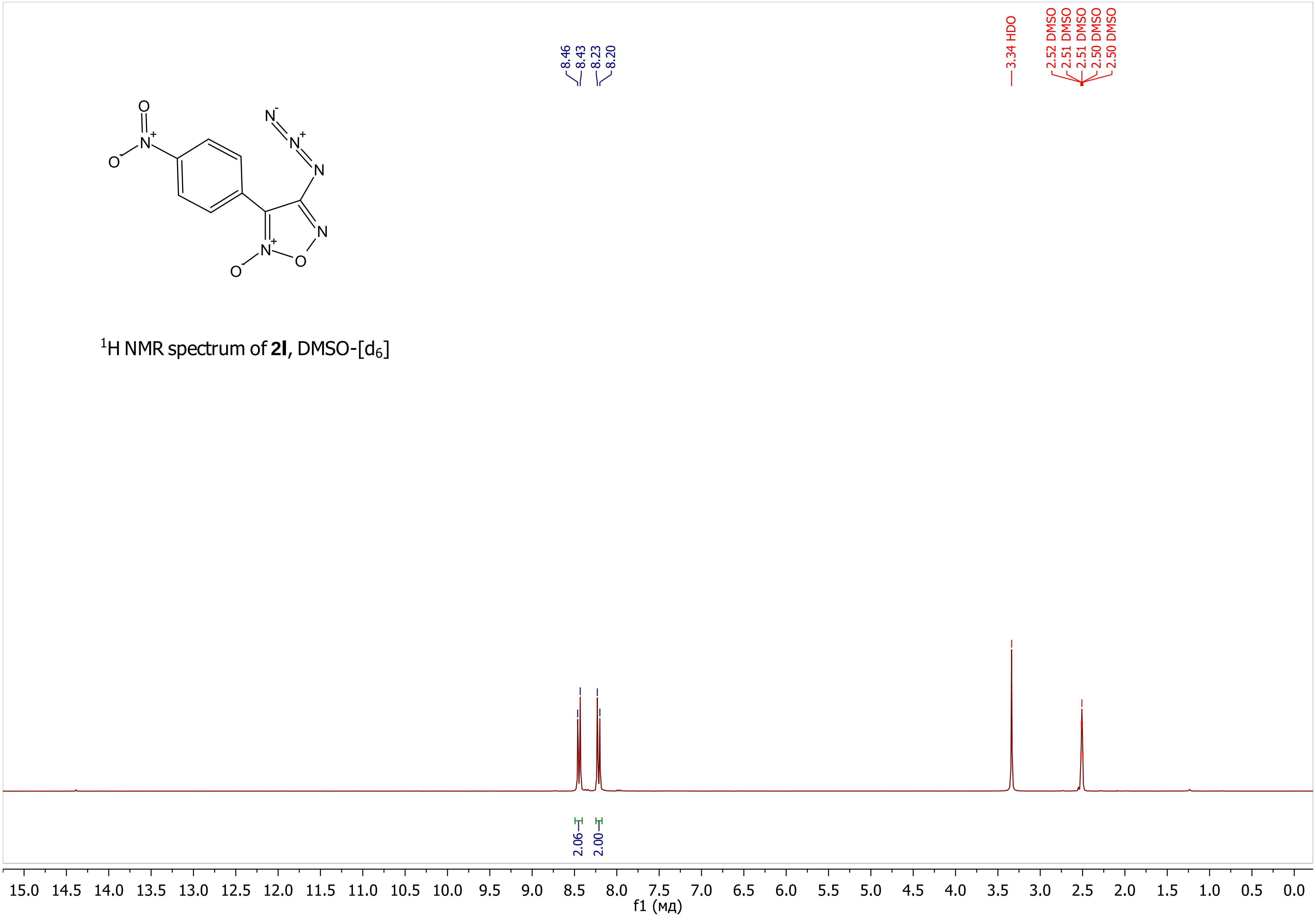

^{1}H NMR spectrum of **2l**, DMSO-[d_6]

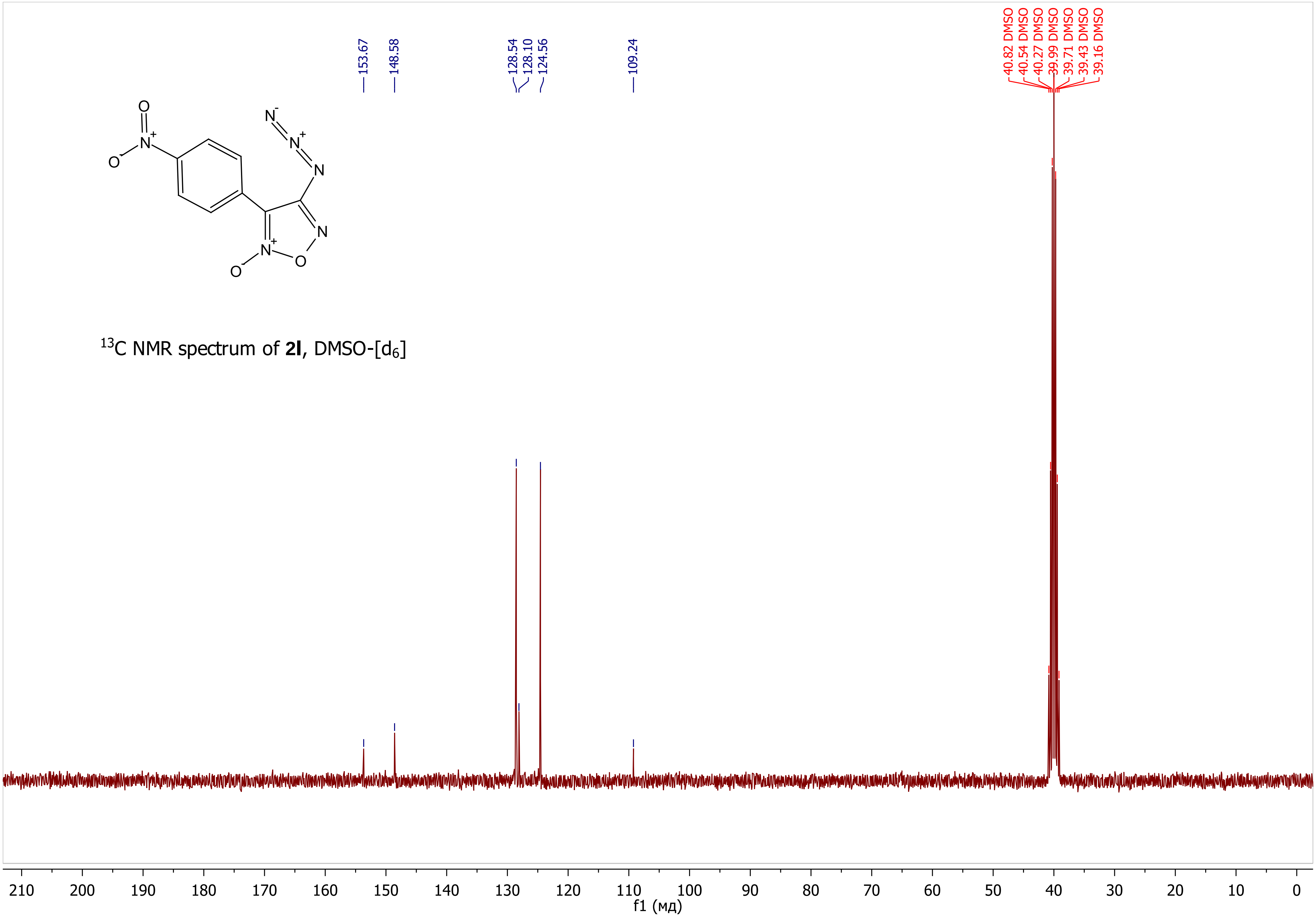

^{13}C NMR spectrum of **2l**, DMSO-[d_6]

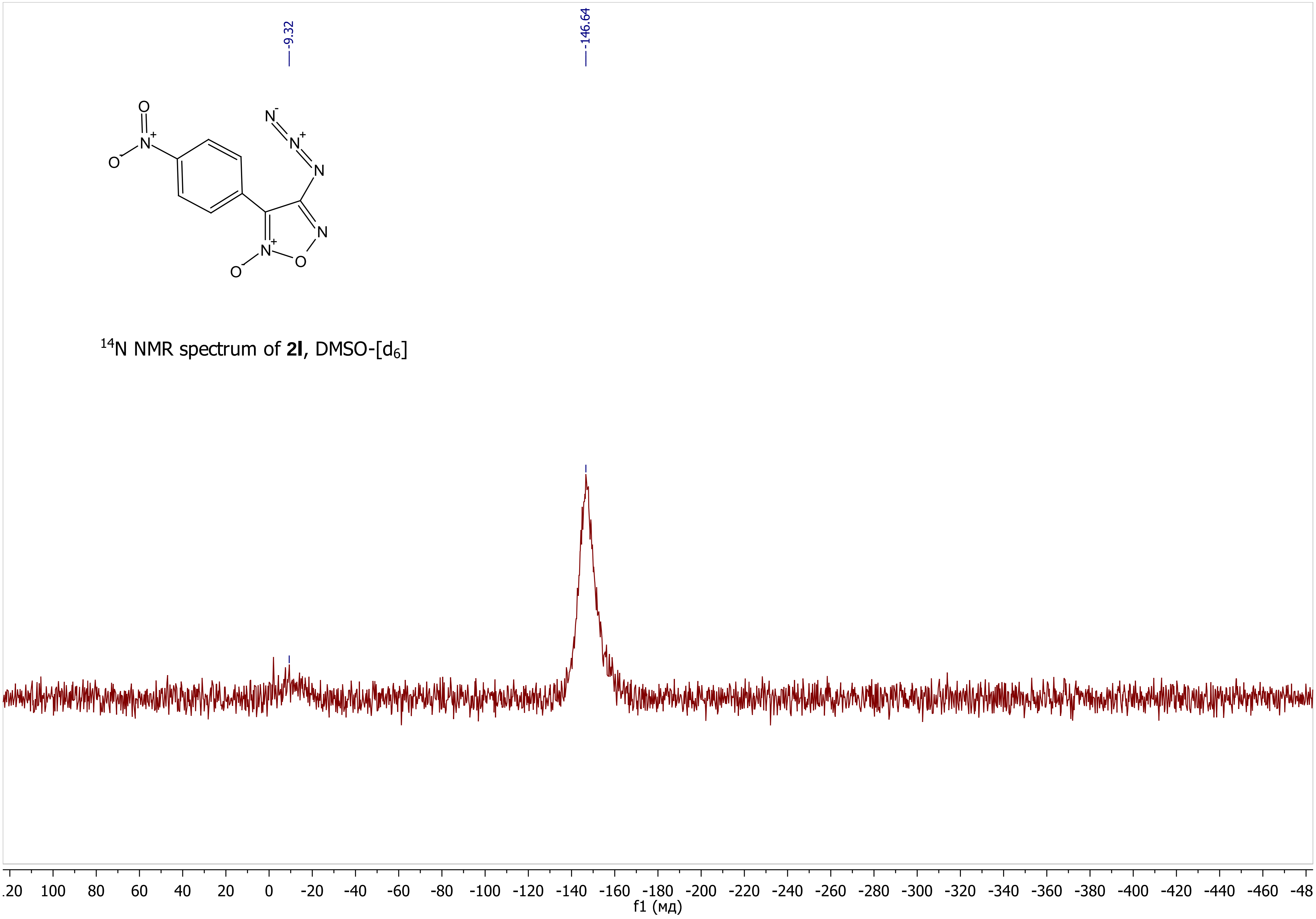

^{14}N NMR spectrum of **2l**, DMSO-[d_6]

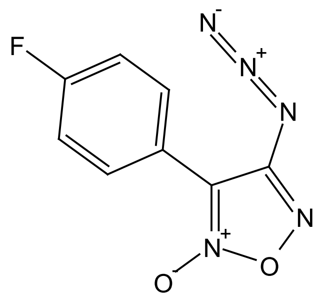

^{1}H NMR spectrum of **2m**, $CDCl_3$

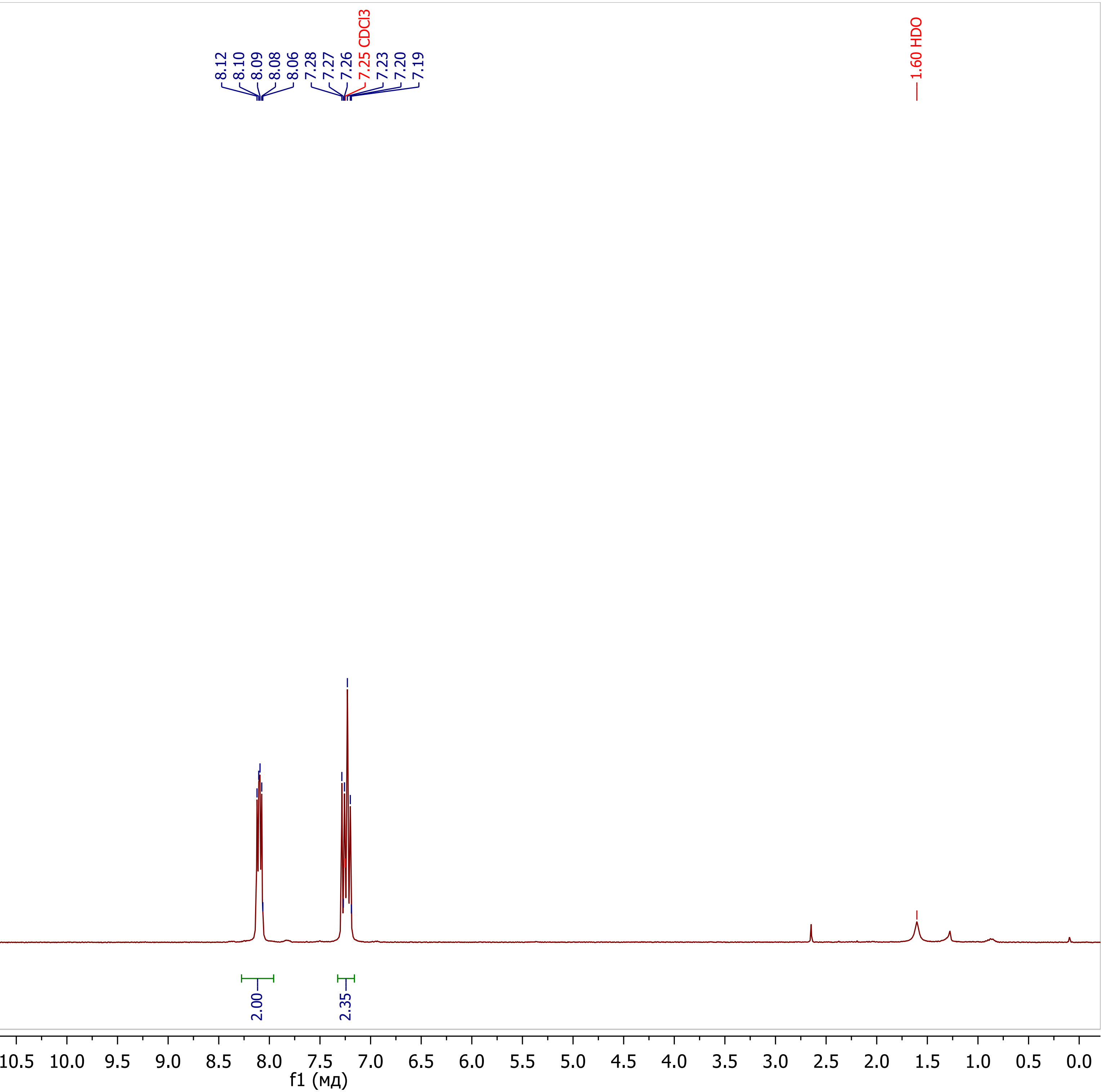

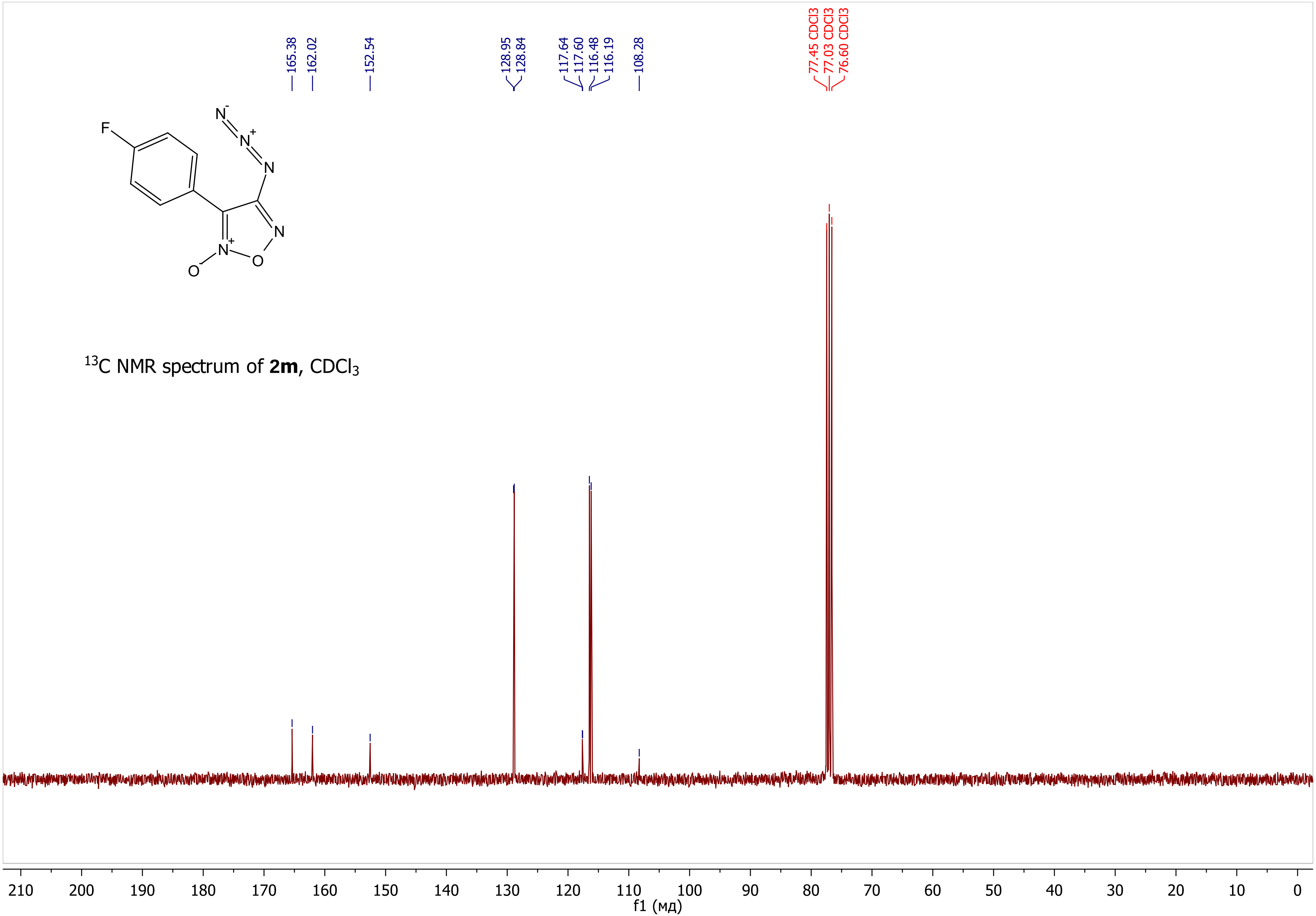

^{13}C NMR spectrum of **2m**, $CDCl_3$

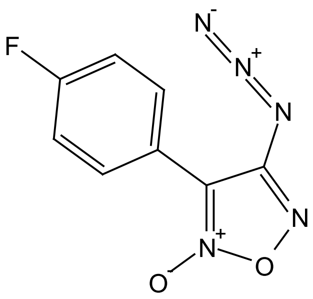

^{14}N NMR spectrum of **2m**, $CDCl_3$

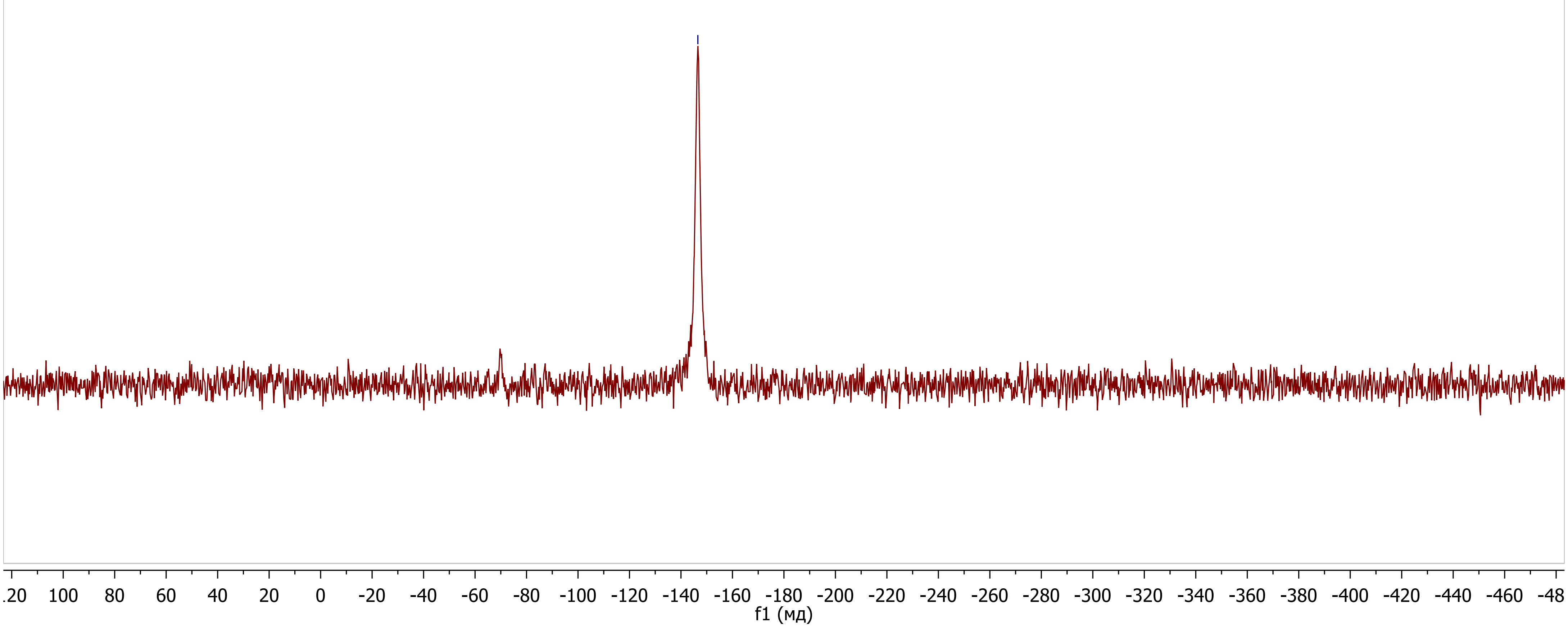

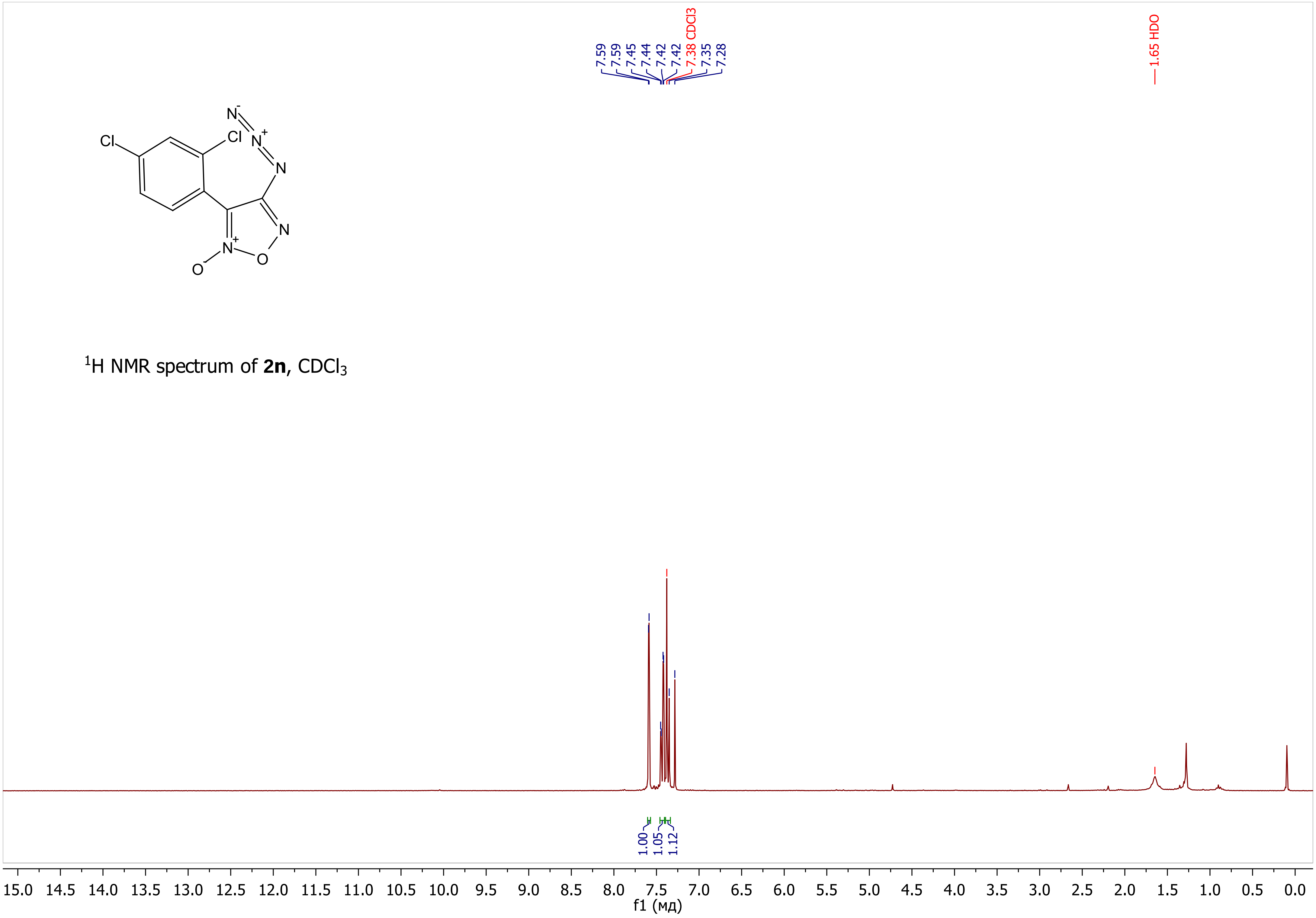

^{1}H NMR spectrum of **2n**, $CDCl_3$

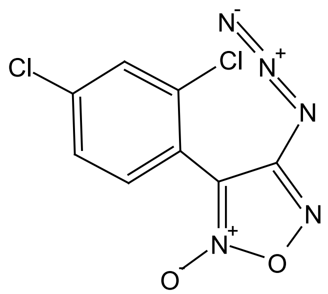

^{13}C NMR spectrum of **2n**, $CDCl_3$

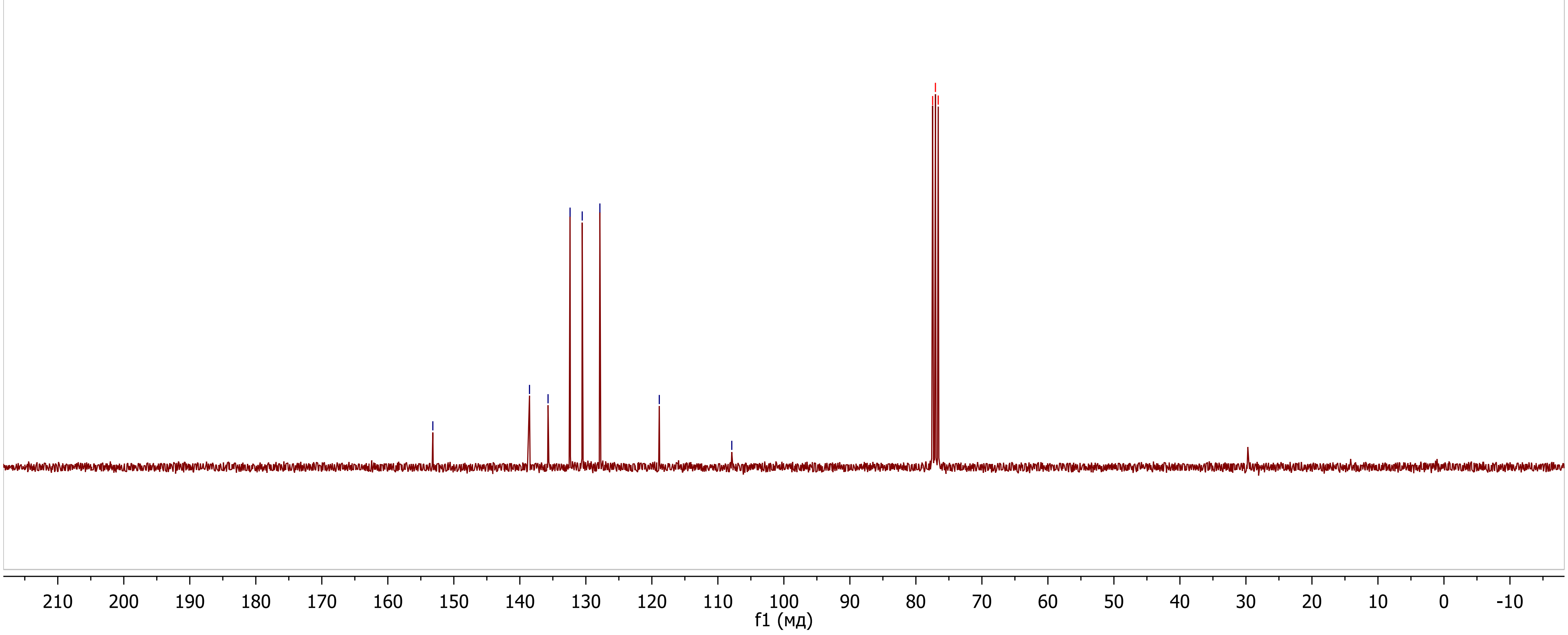

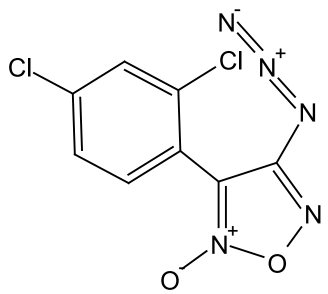

[14]N NMR spectrum of **2n**, $CDCl_3$

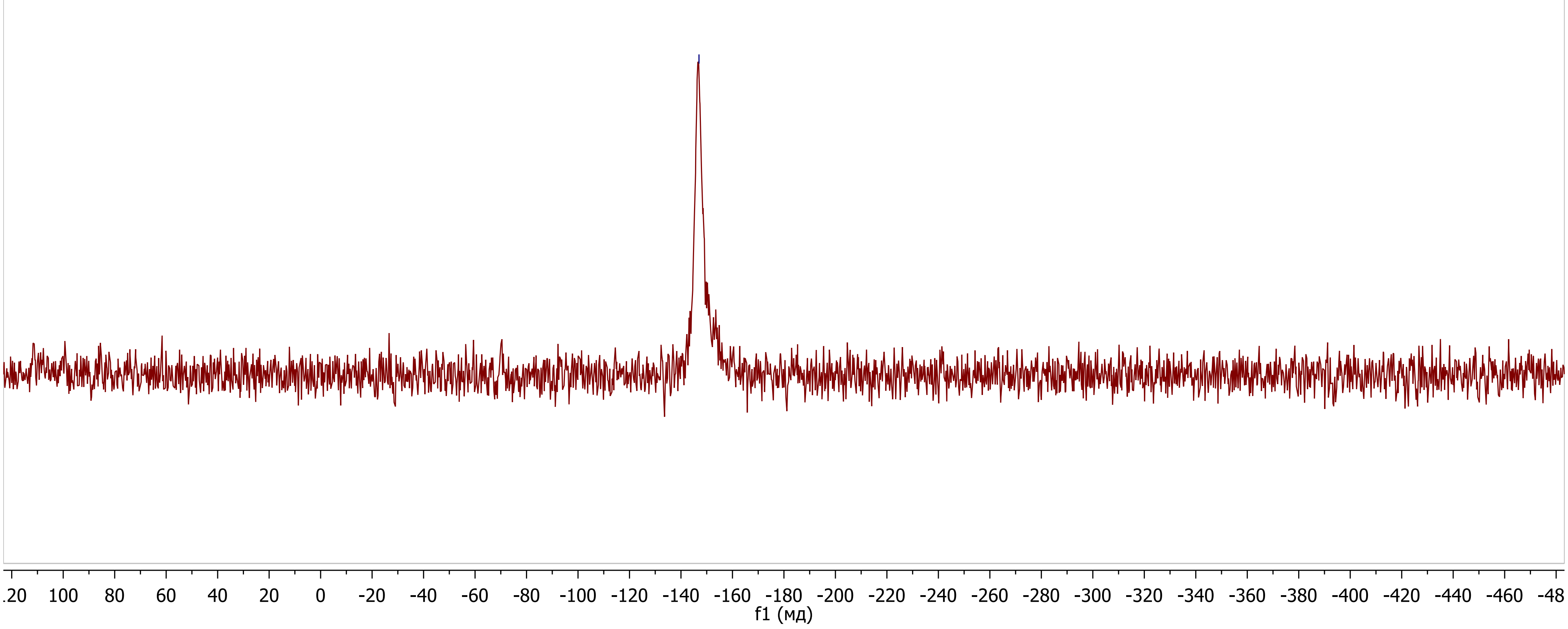

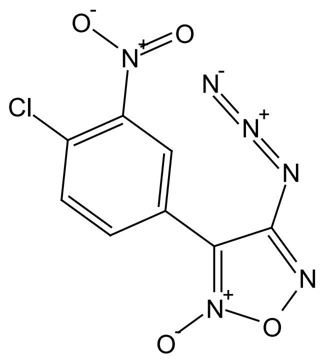

^{1}H NMR spectrum of **2o**, Acetone-[d_6]

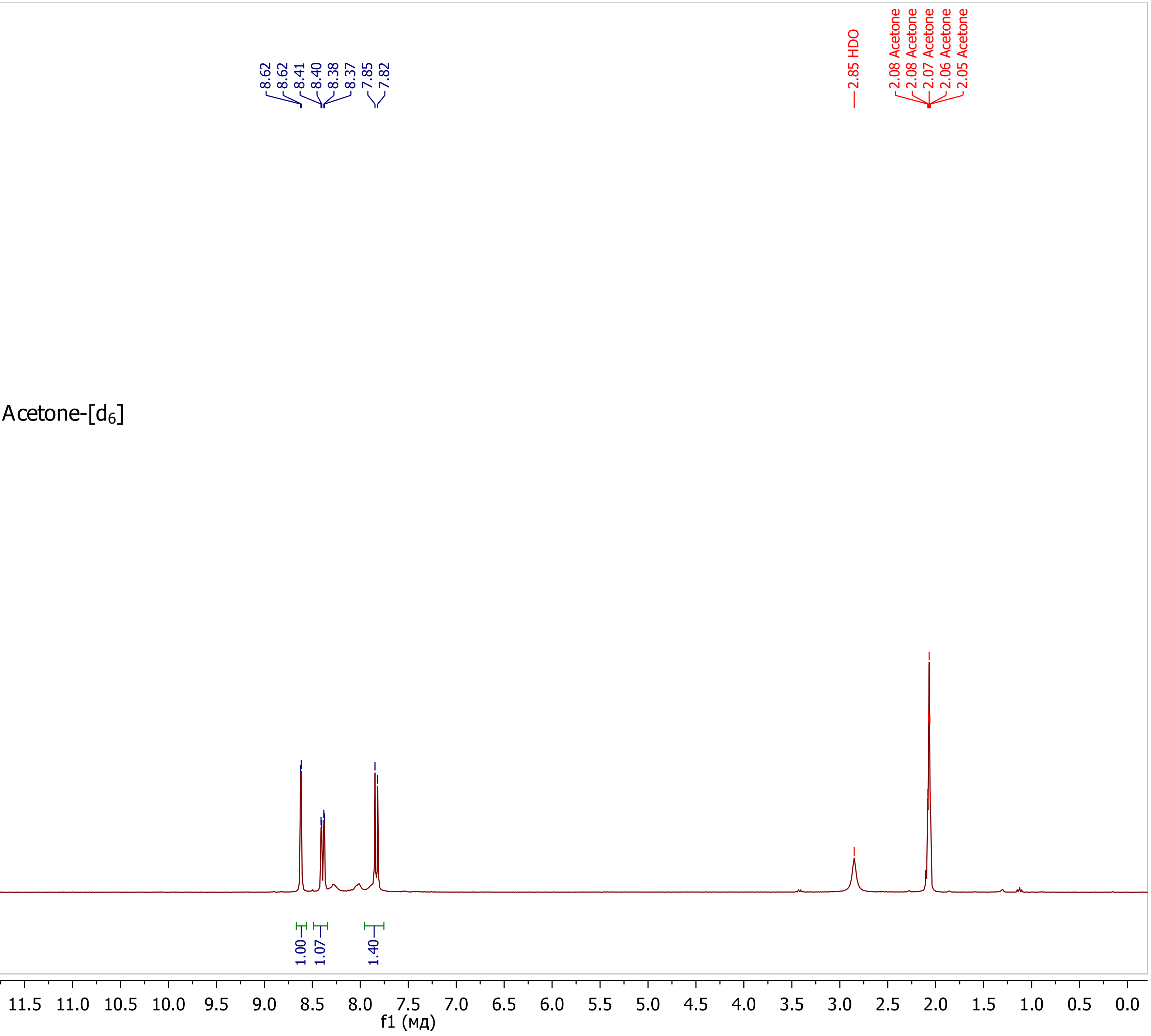

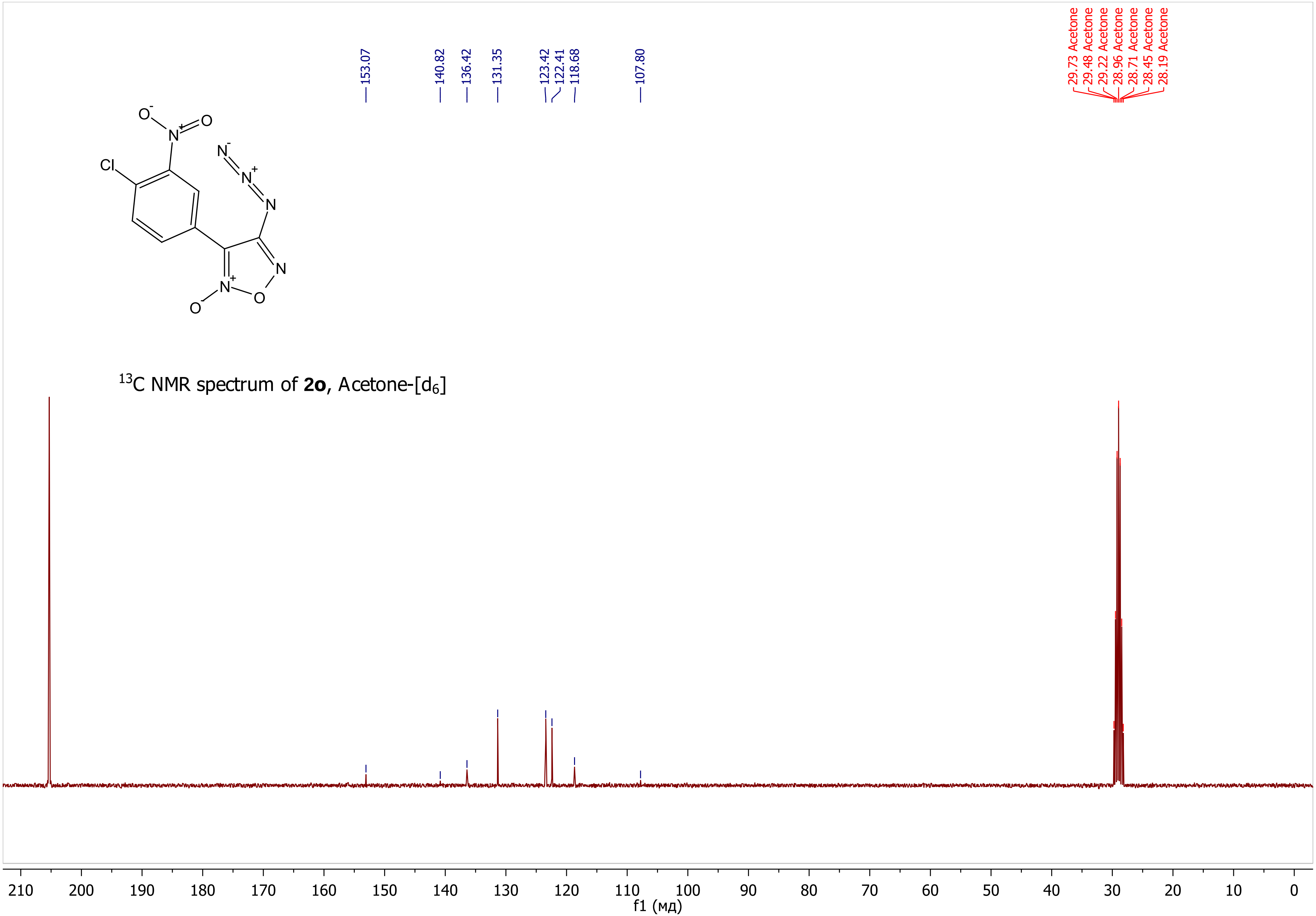

^{13}C NMR spectrum of **2o**, Acetone-[d$_6$]

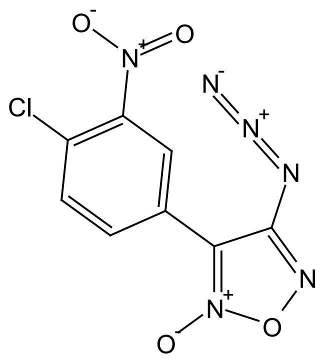

^{14}N NMR spectrum of **2o**, Acetone-[d$_6$]

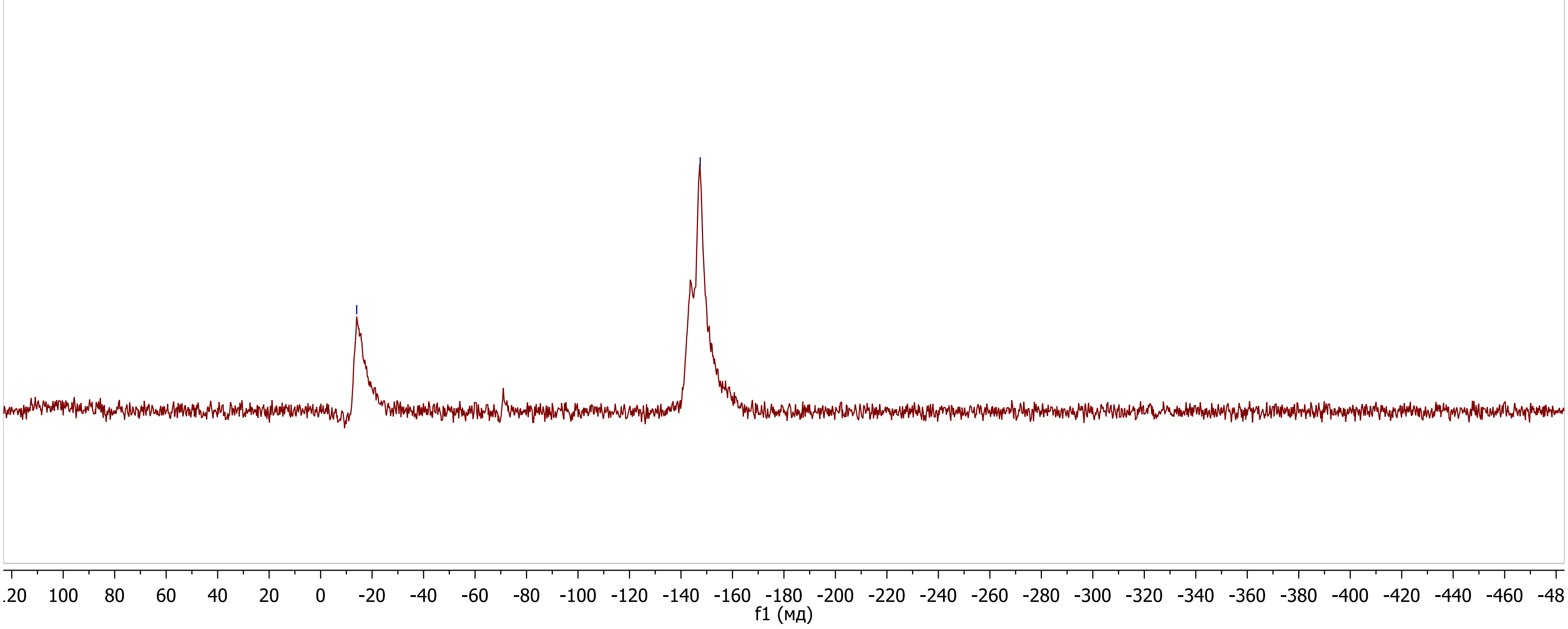

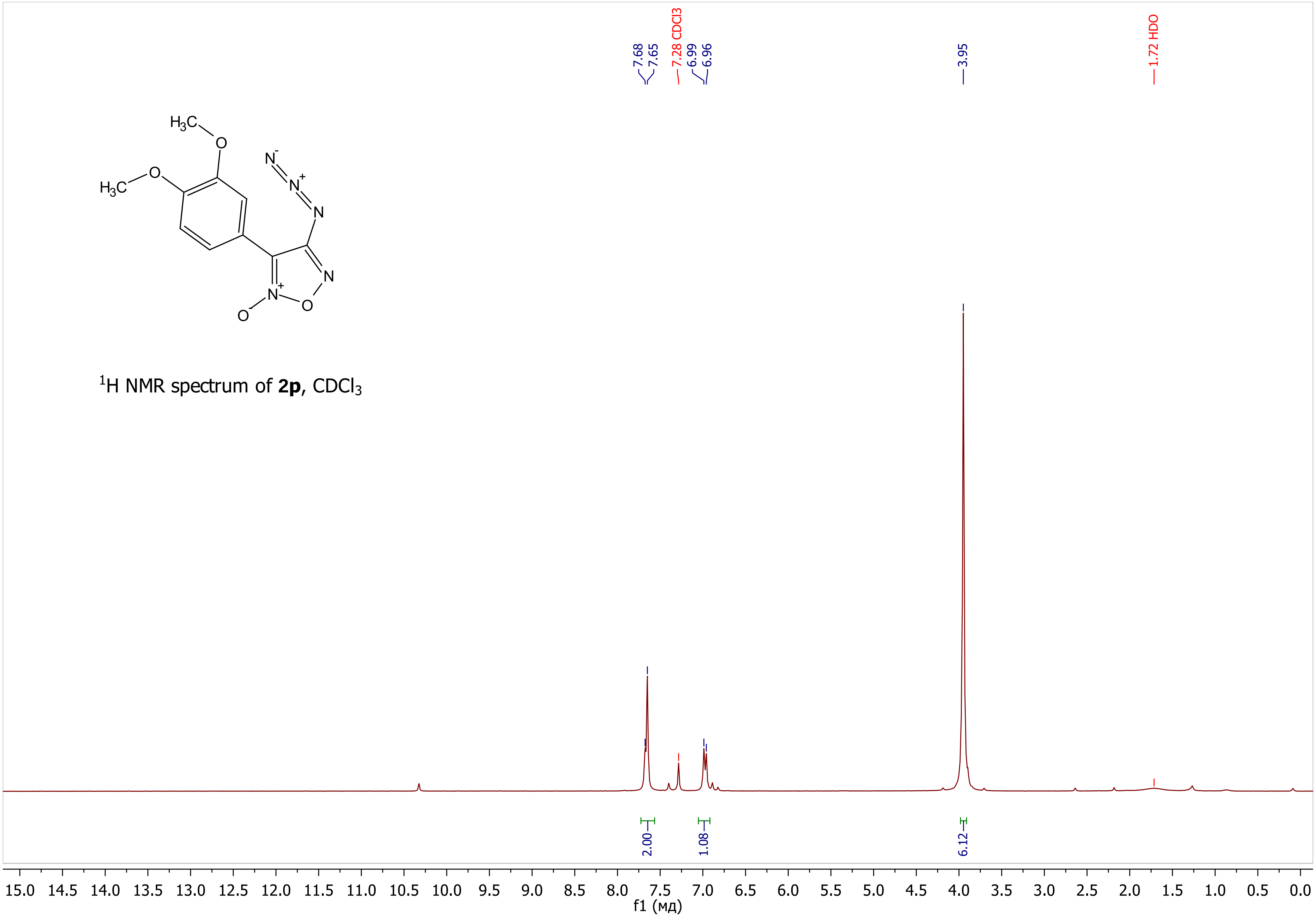

^{1}H NMR spectrum of **2p**, $CDCl_3$

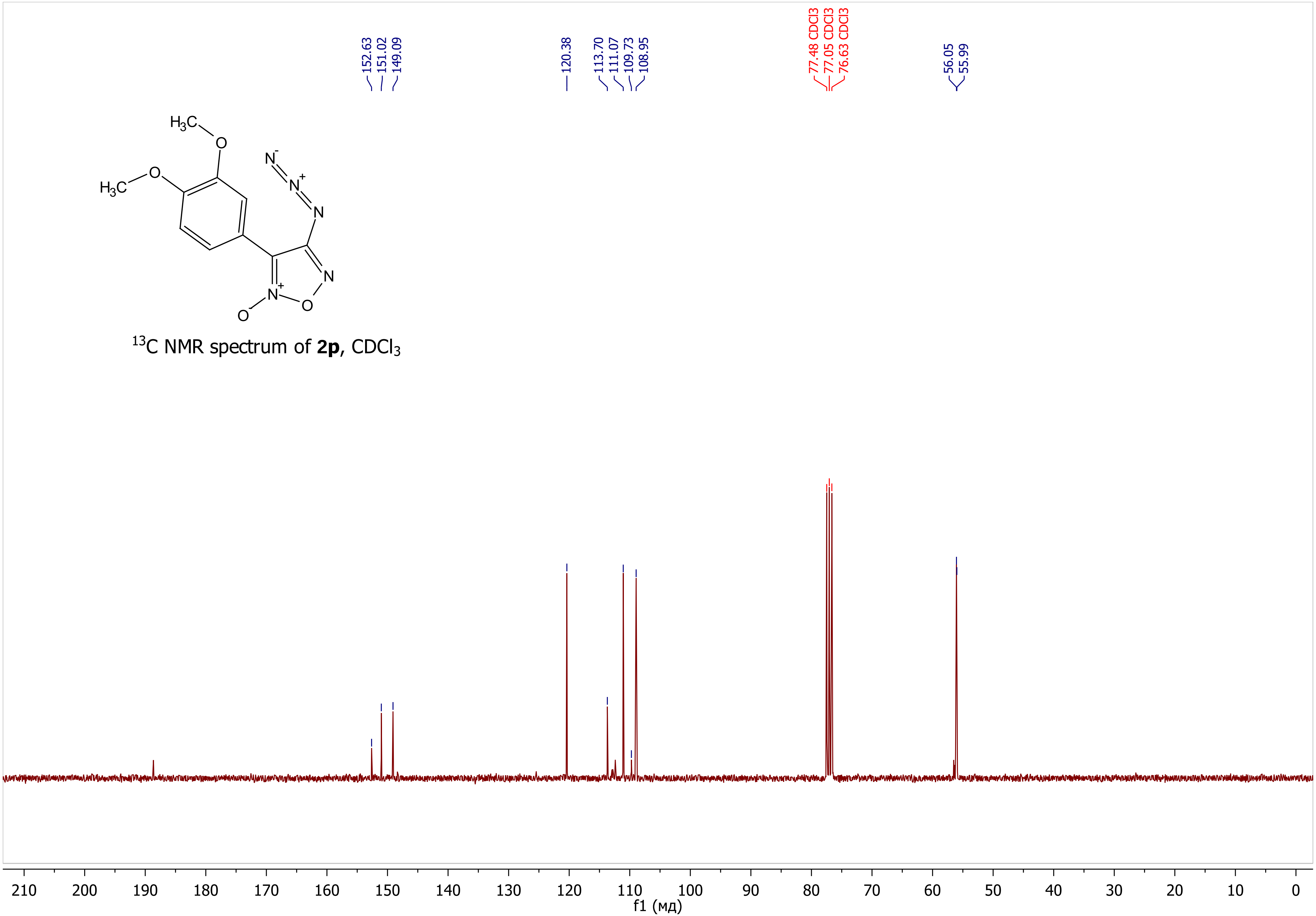
152.63
151.02
149.09
120.38
113.70
111.07
109.73
108.95
77.48 CDCl3
77.05 CDCl3
76.63 CDCl3
56.05
55.99
^{13}C NMR spectrum of **2p**, $CDCl_3$
210
200
190
180
170
160
150
140
130
120
110
100
90
80
70
60
50
40
30
20
10
0
f1 (мд)

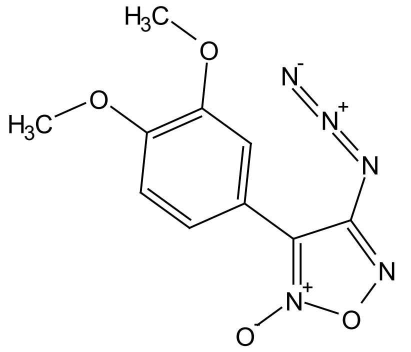

^{14}N NMR spectrum of **2p**, $CDCl_3$

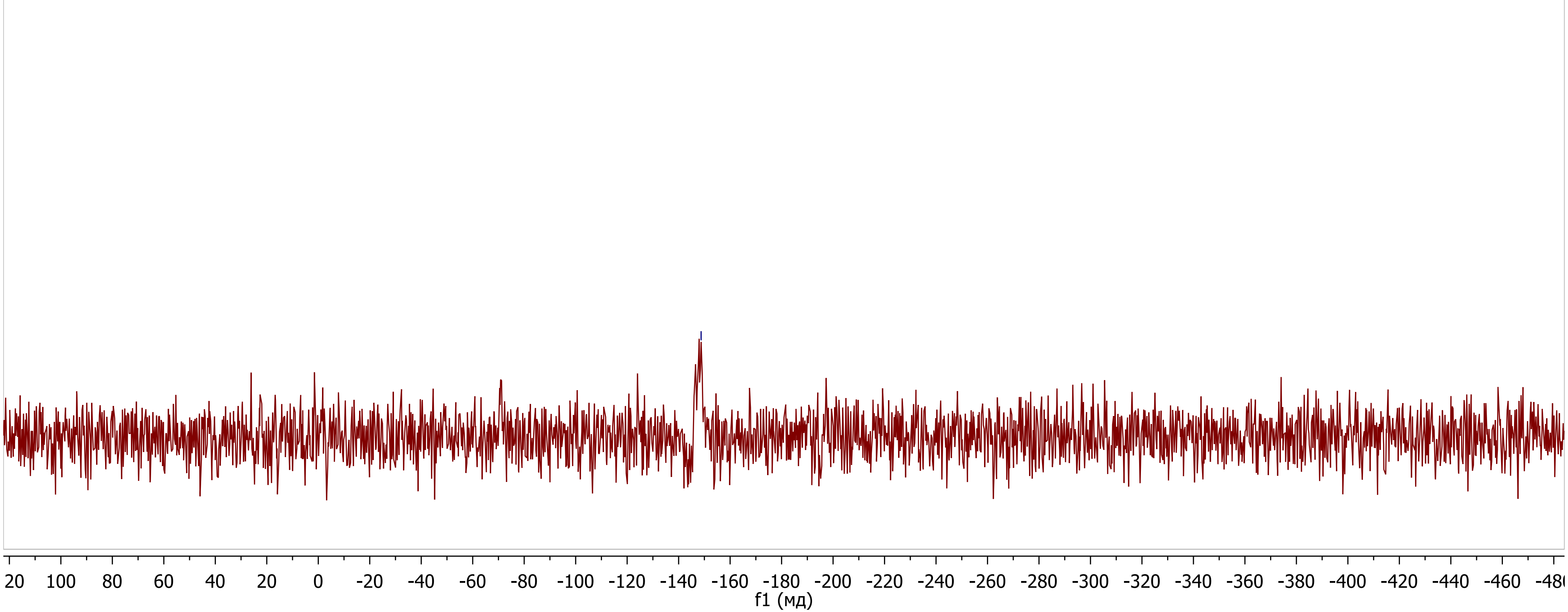

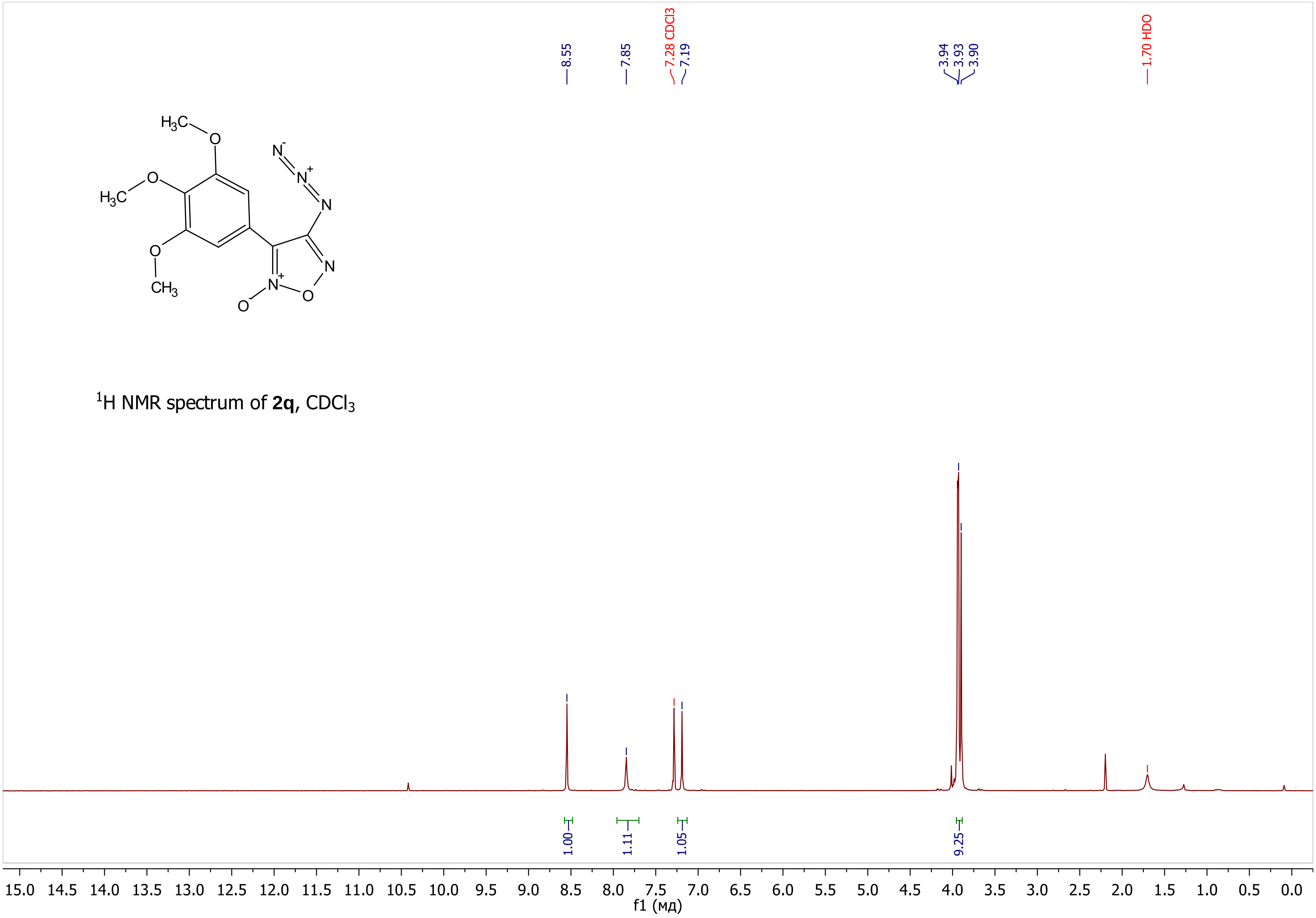

^{1}H NMR spectrum of **2q**, $CDCl_3$

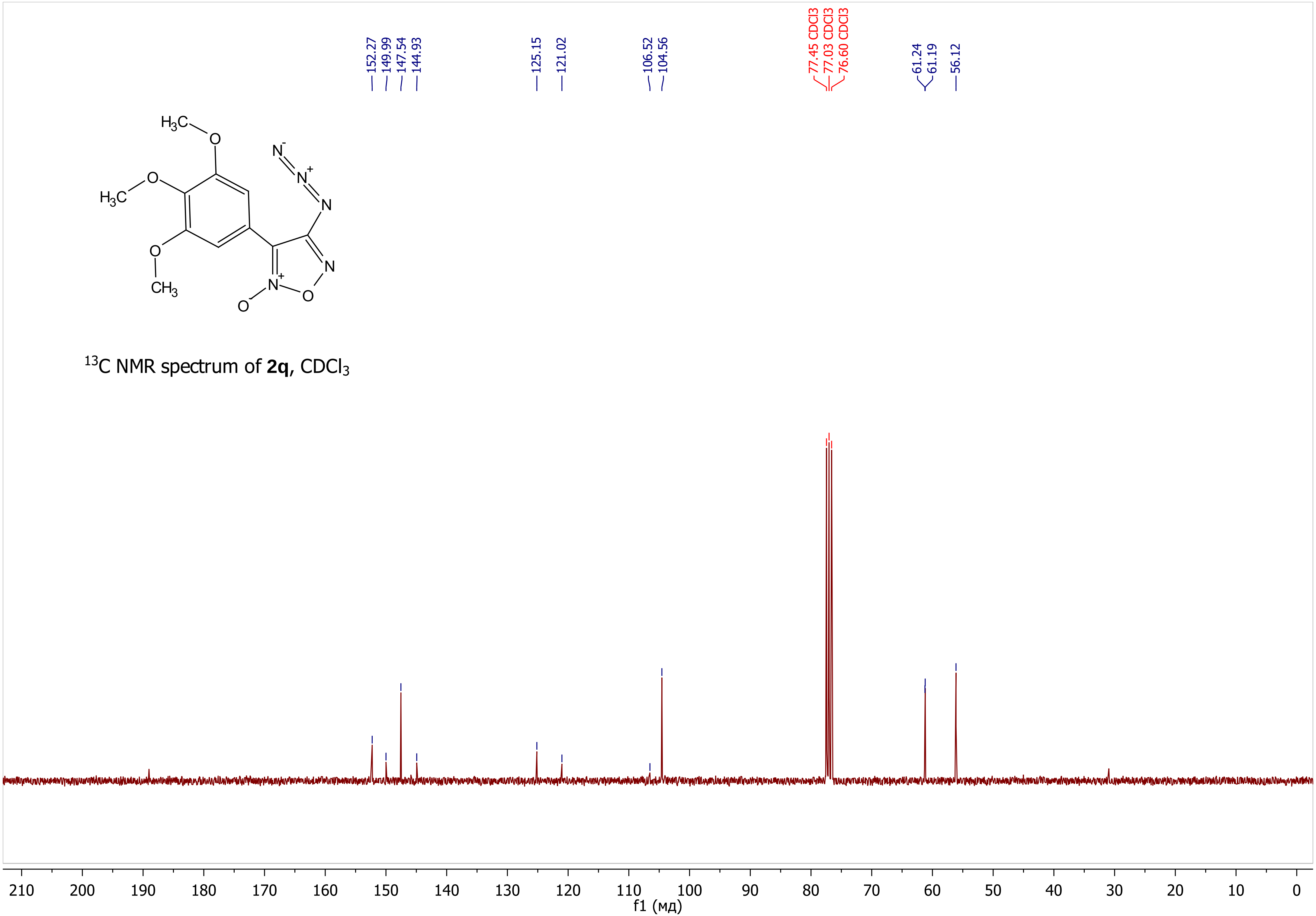

^{13}C NMR spectrum of **2q**, $CDCl_3$

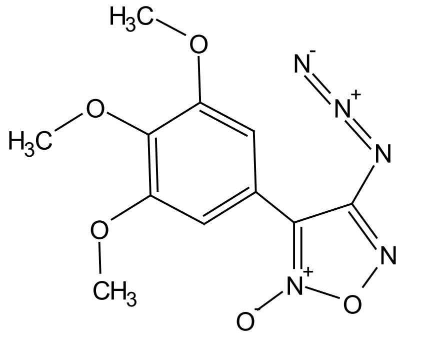

^{14}N NMR spectrum of **2q**, $CDCl_3$

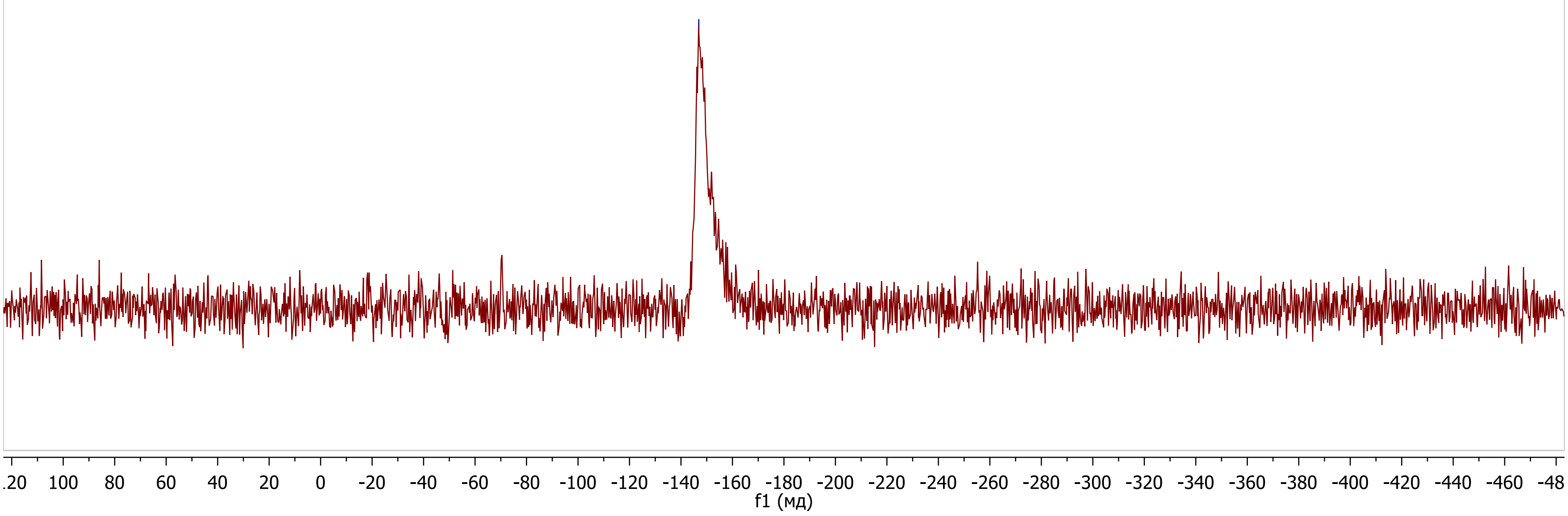

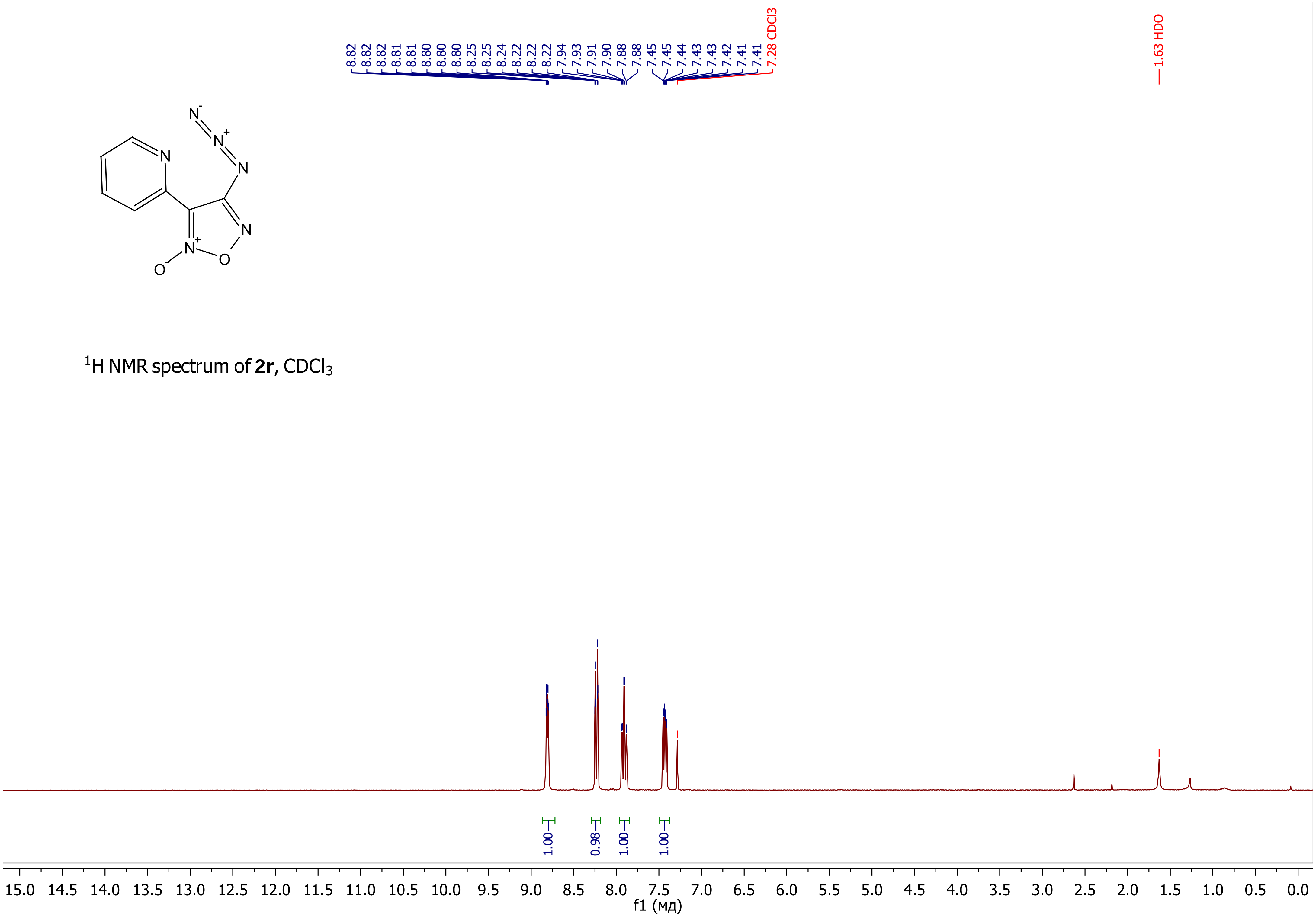

^{1}H NMR spectrum of **2r**, $CDCl_3$

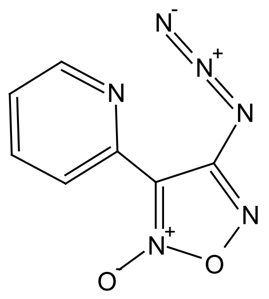

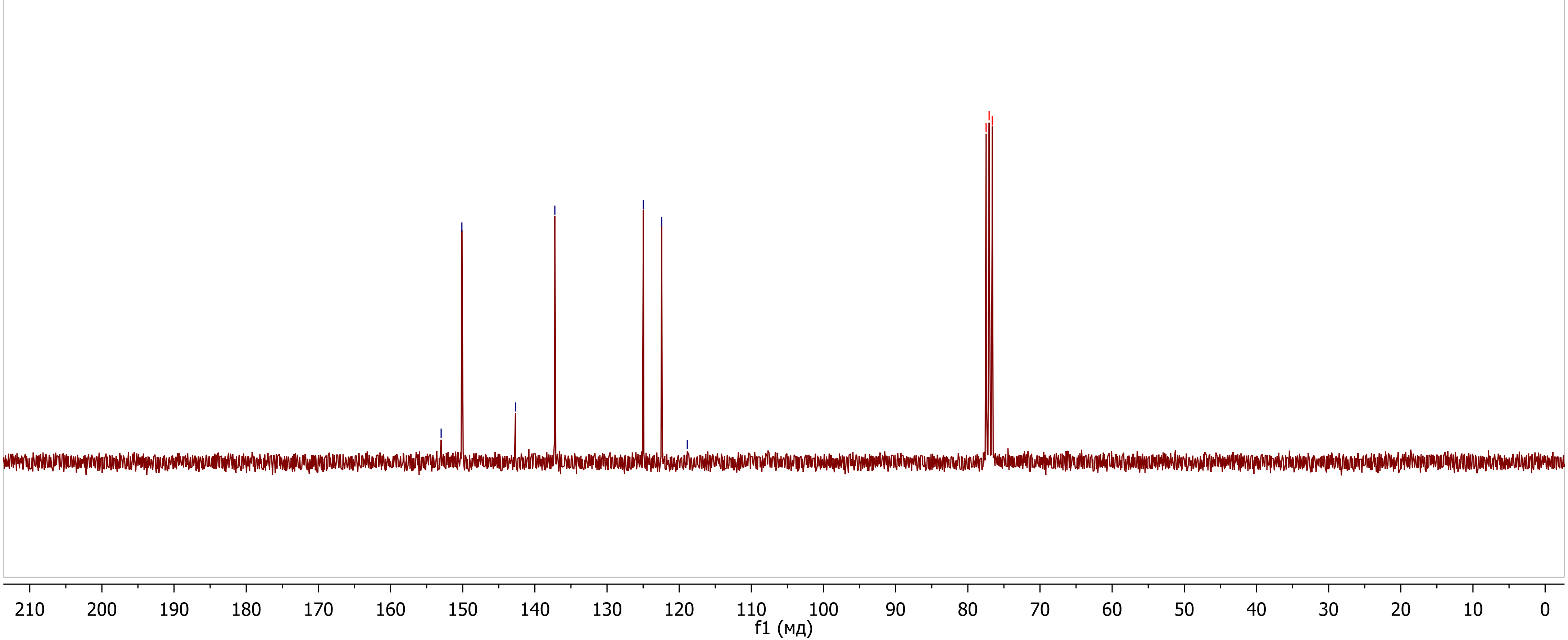

^{13}C NMR spectrum of **2r**, $CDCl_3$

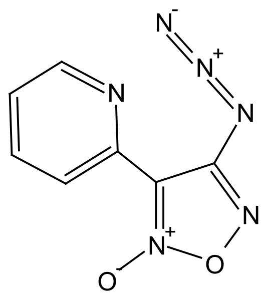

^{14}N NMR spectrum of **2r**, $CDCl_3$

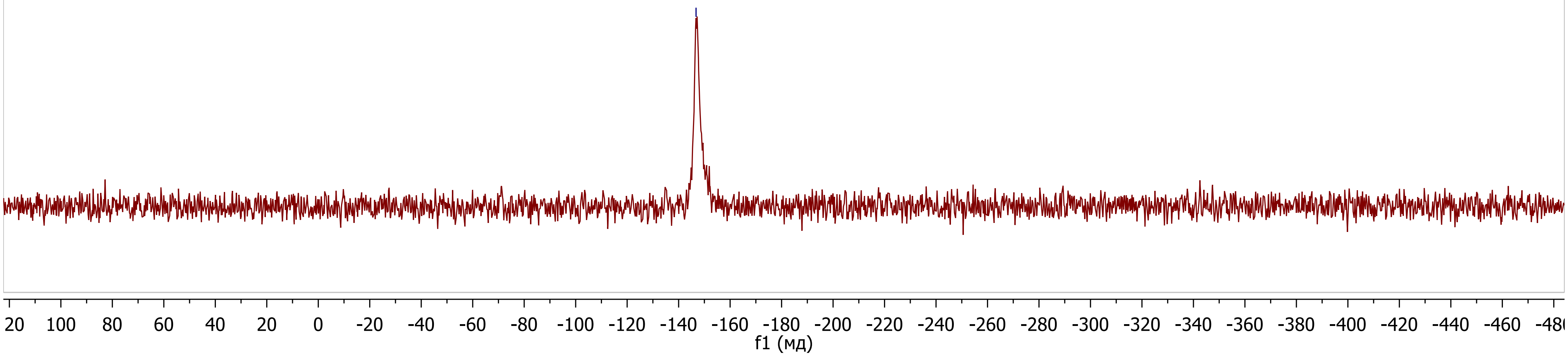

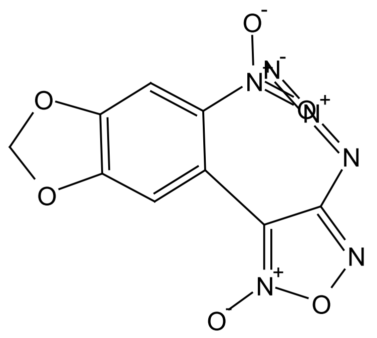

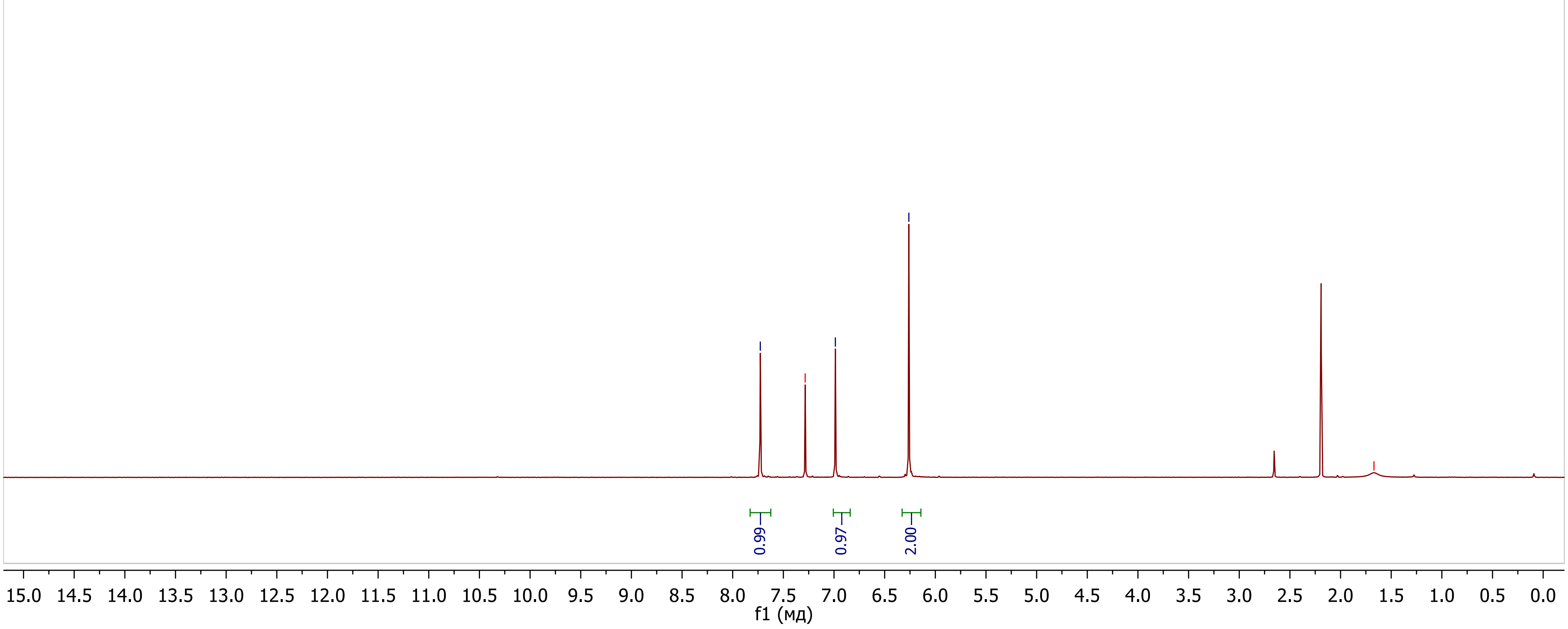

^{1}H NMR spectrum of **2s**, $CDCl_3$

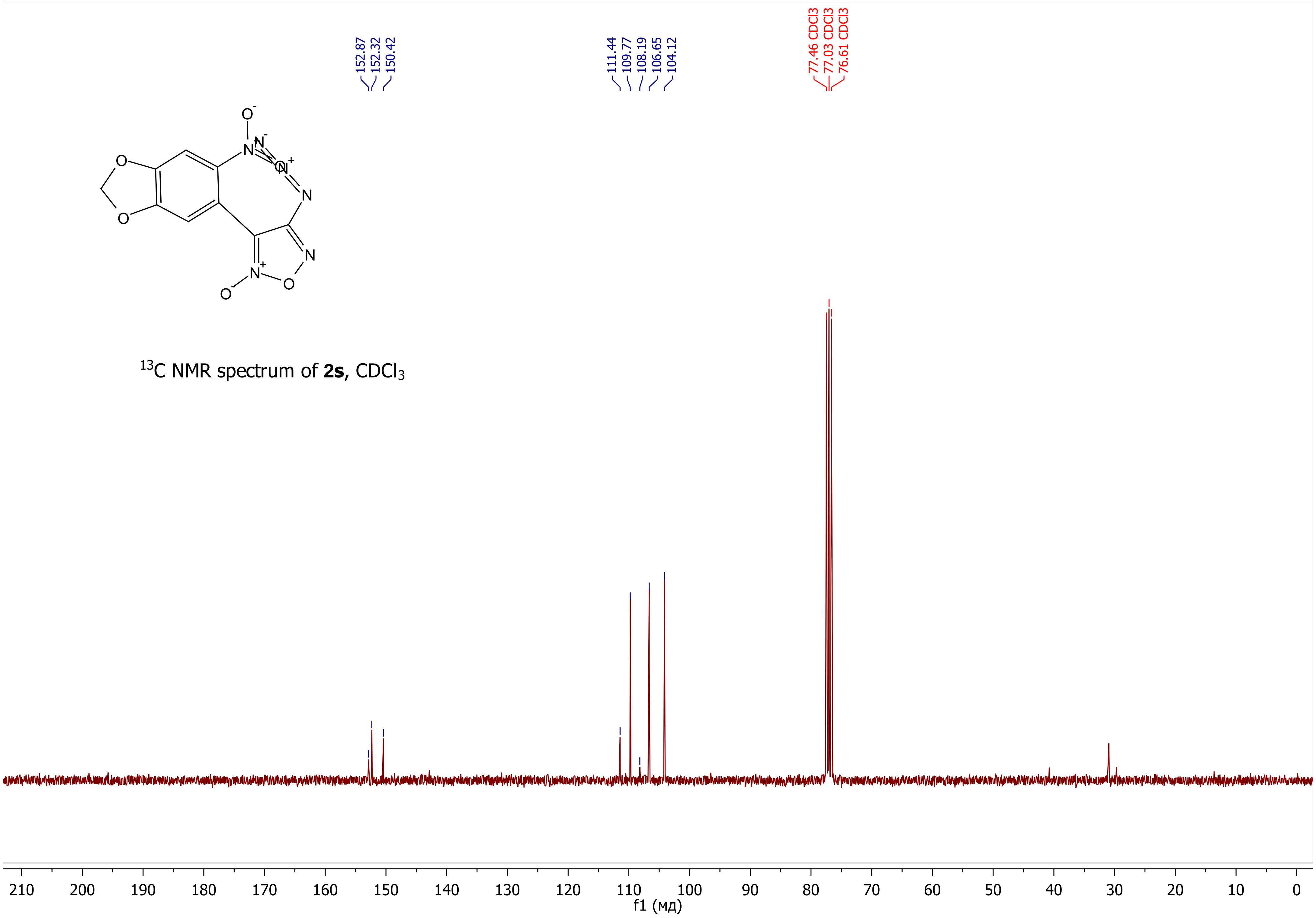

^{13}C NMR spectrum of **2s**, $CDCl_3$

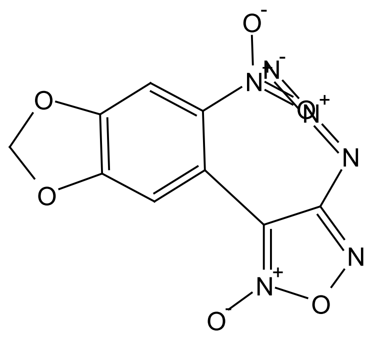

^{14}N NMR spectrum of **2s**, $CDCl_3$

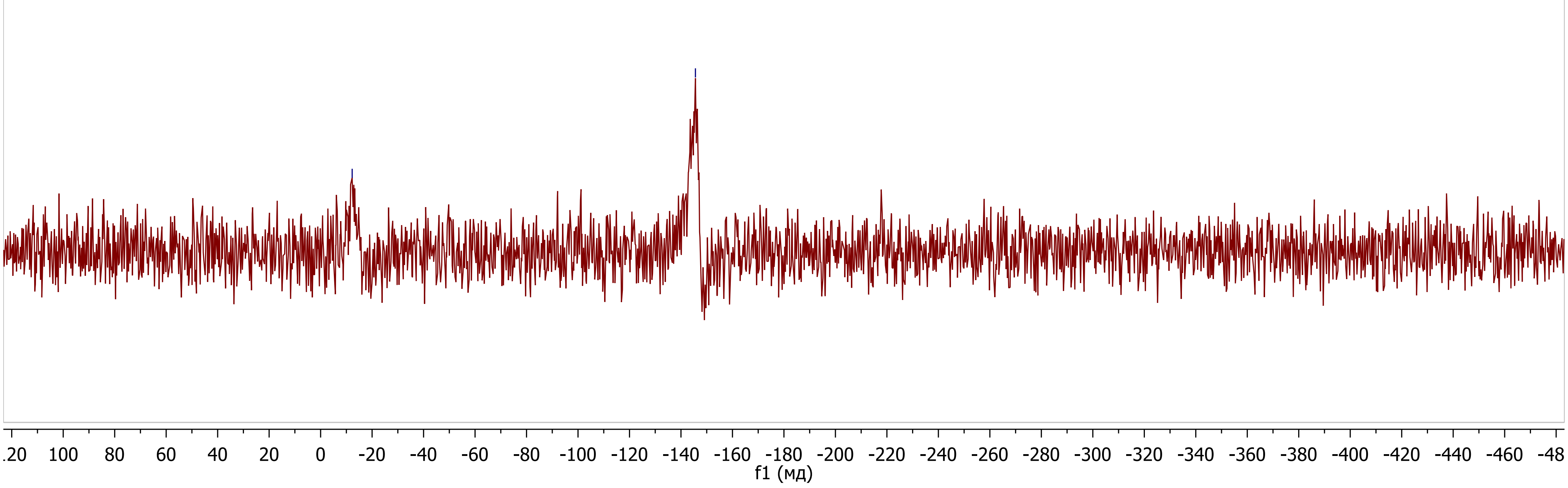

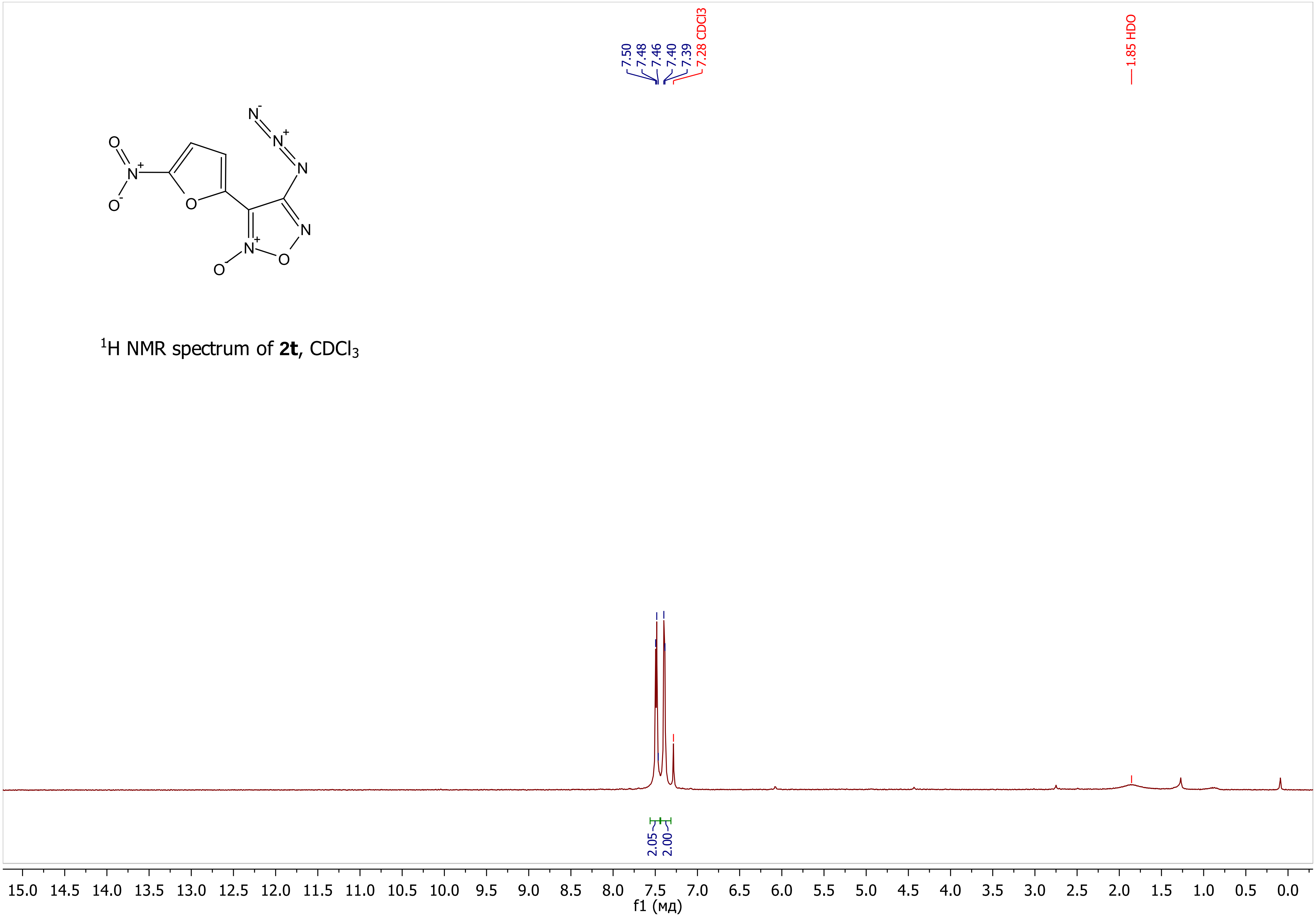

^{1}H NMR spectrum of **2t**, $CDCl_3$

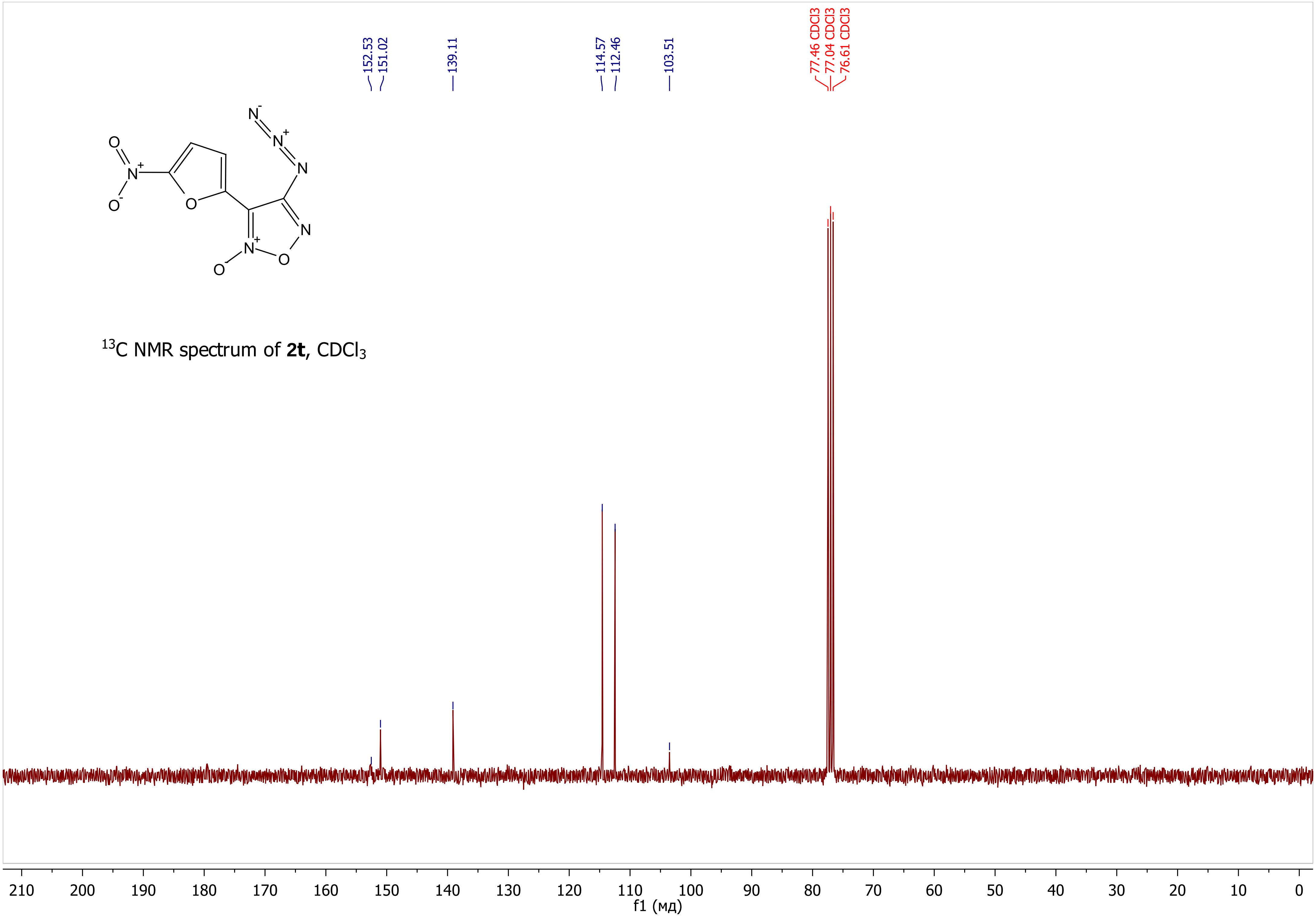

^{13}C NMR spectrum of **2t**, $CDCl_3$

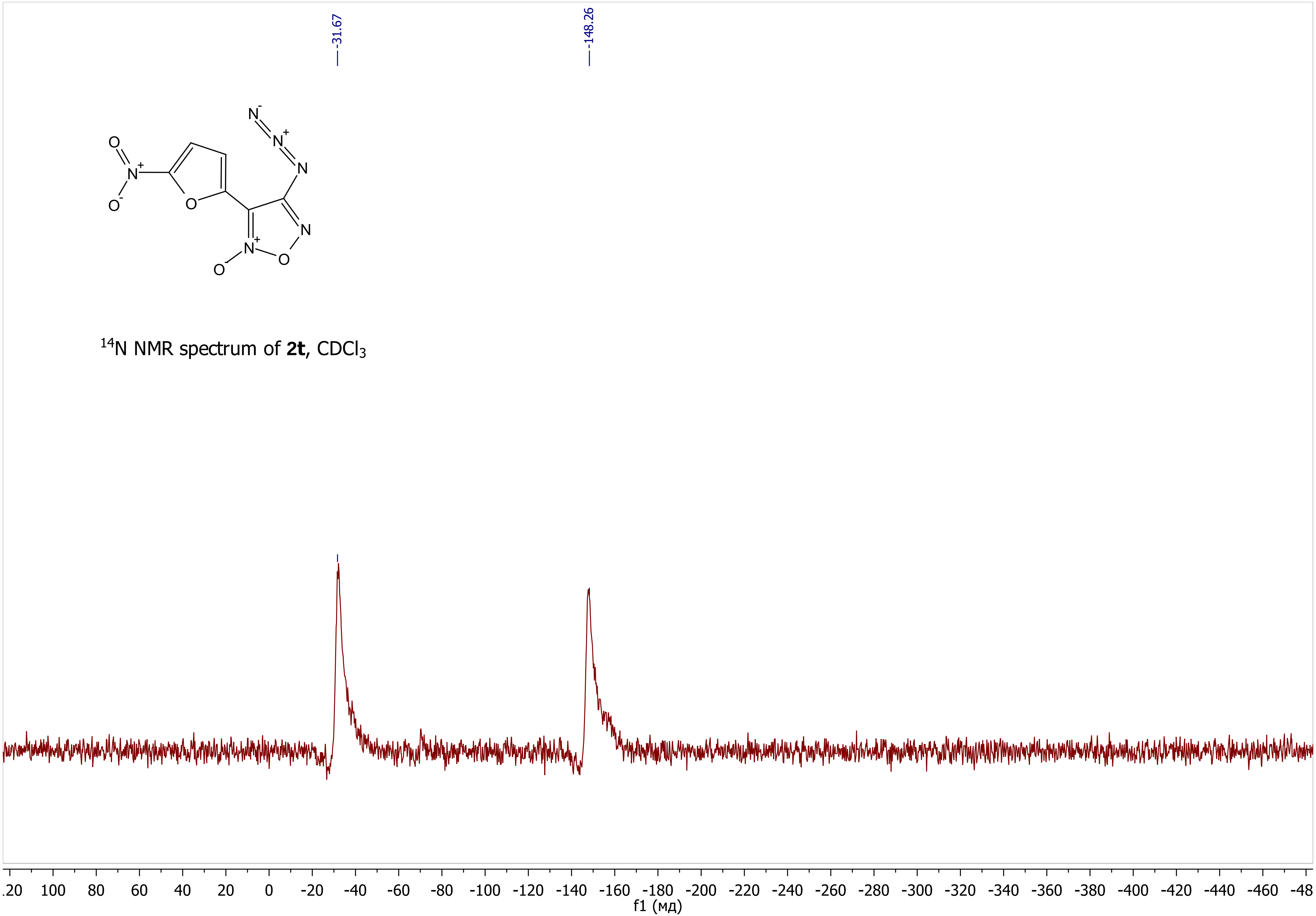

^{14}N NMR spectrum of **2t**, $CDCl_3$

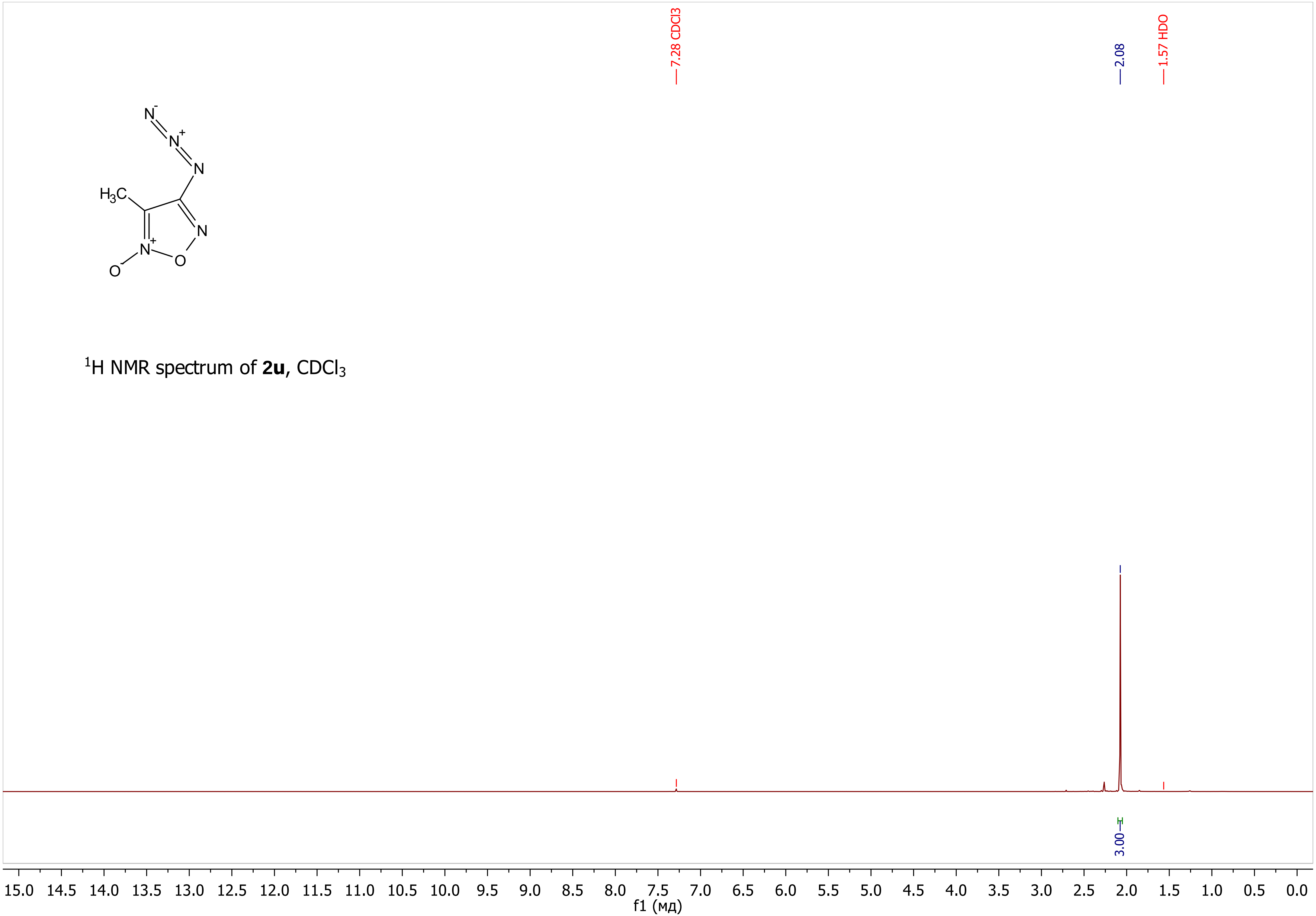

^{1}H NMR spectrum of **2u**, $CDCl_3$

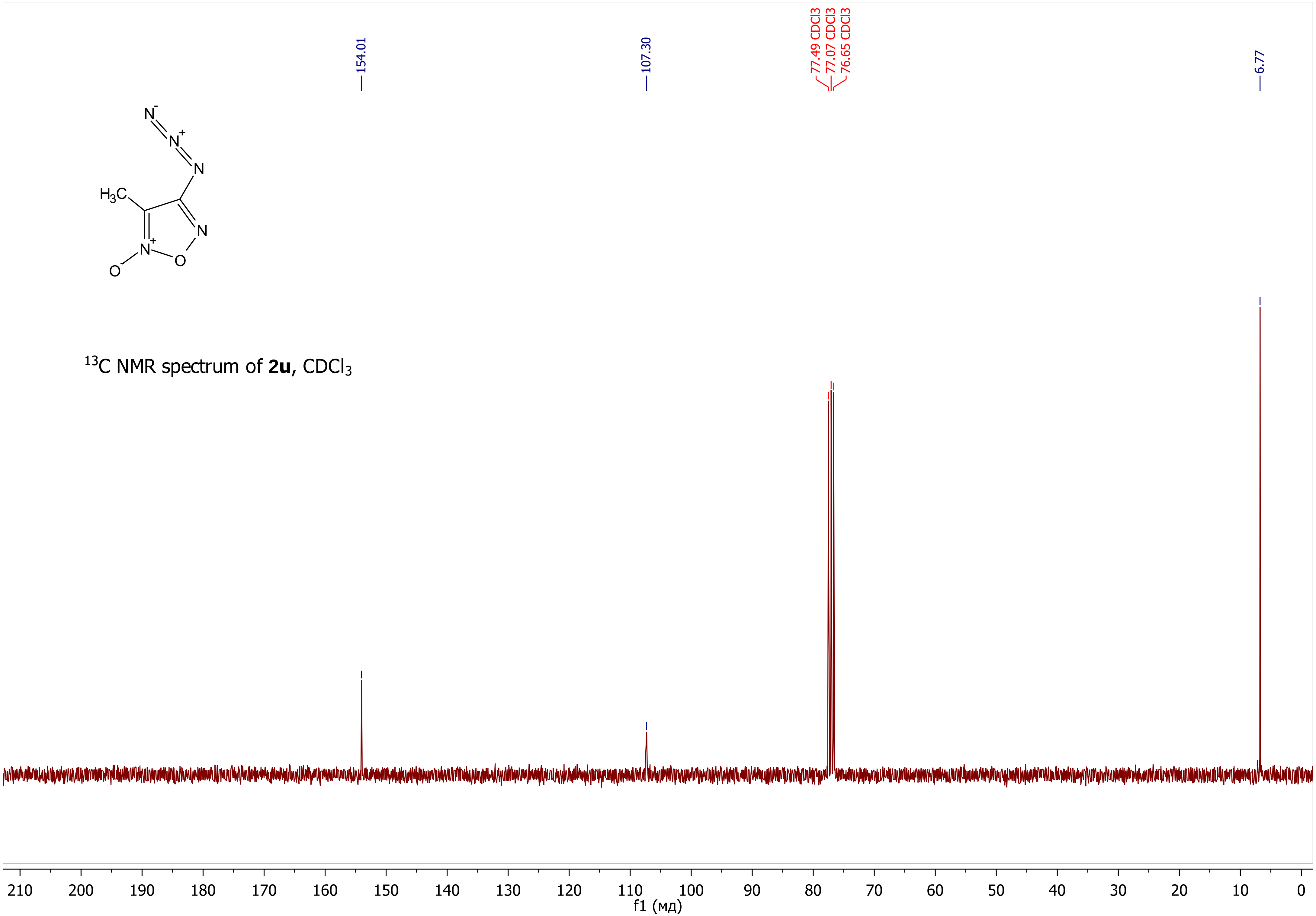

154.01
107.30
77.49 CDCl3
77.07 CDCl3
76.65 CDCl3
6.77
H3C
13C NMR spectrum of 2u, CDCl3
210 200 190 180 170 160 150 140 130 120 110 100 90 80 70 60 50 40 30 20 10 0
f1 (мд)

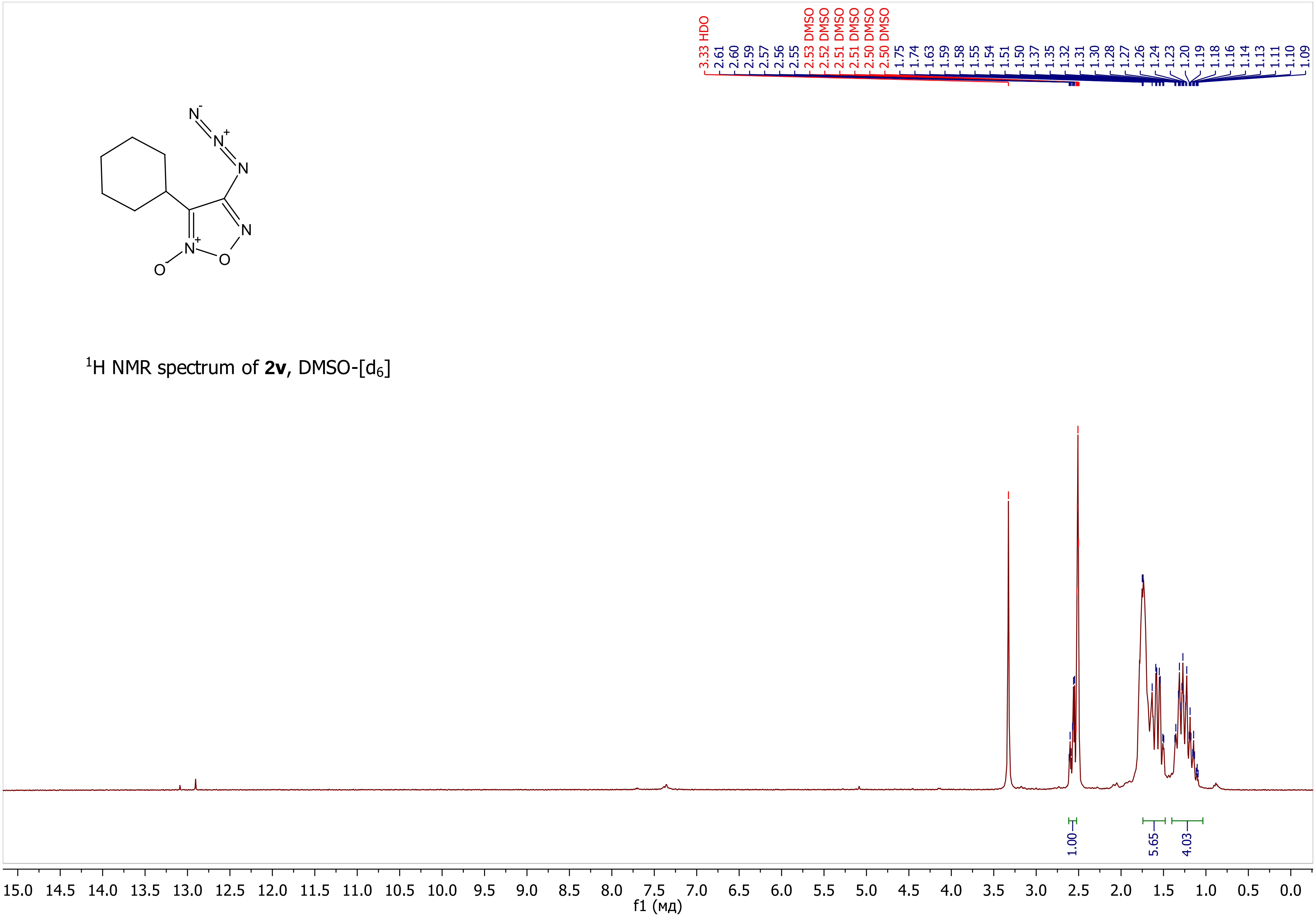

^{1}H NMR spectrum of **2v**, DMSO-[d$_6$]

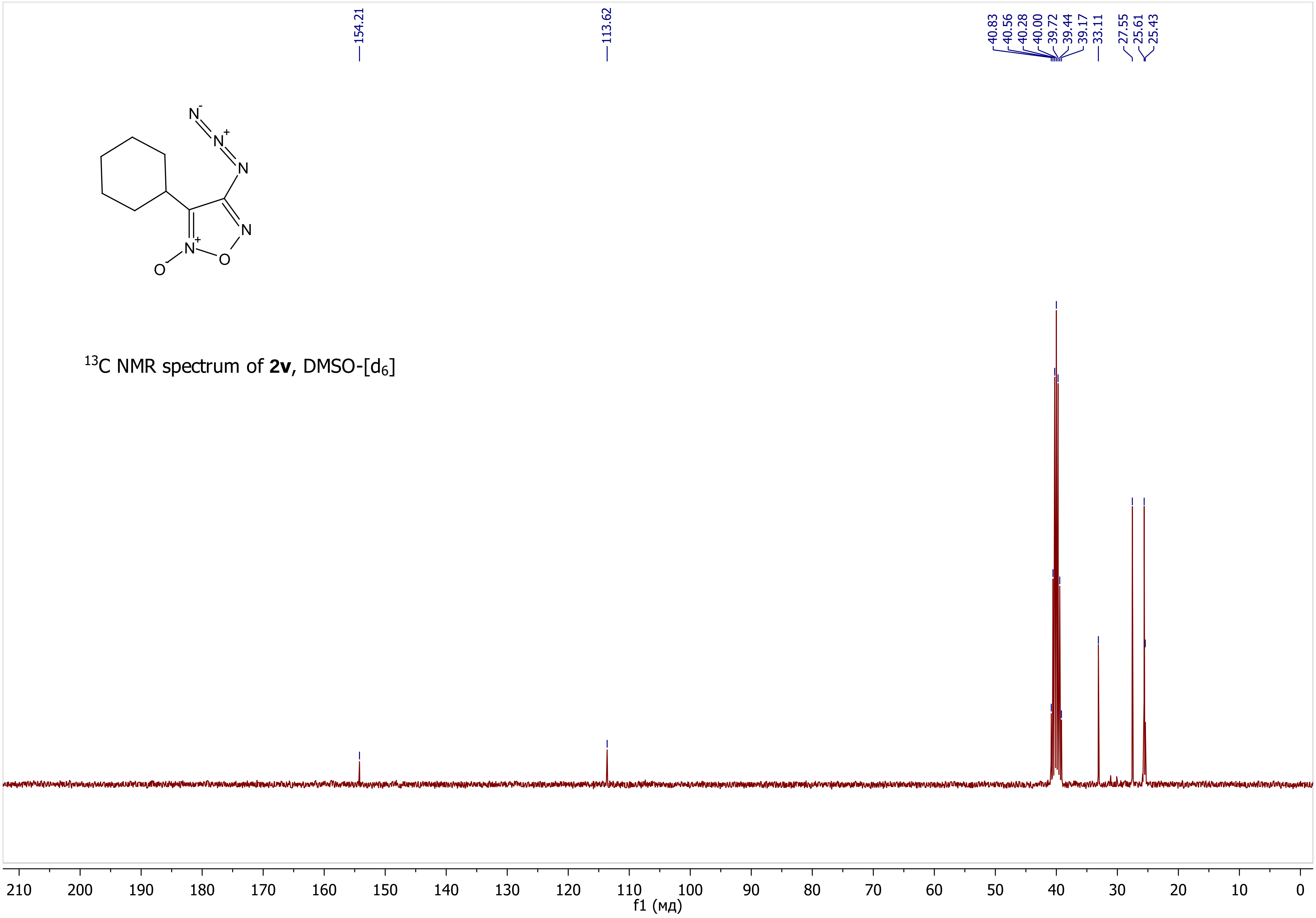

^{13}C NMR spectrum of **2v**, DMSO-[d_6]

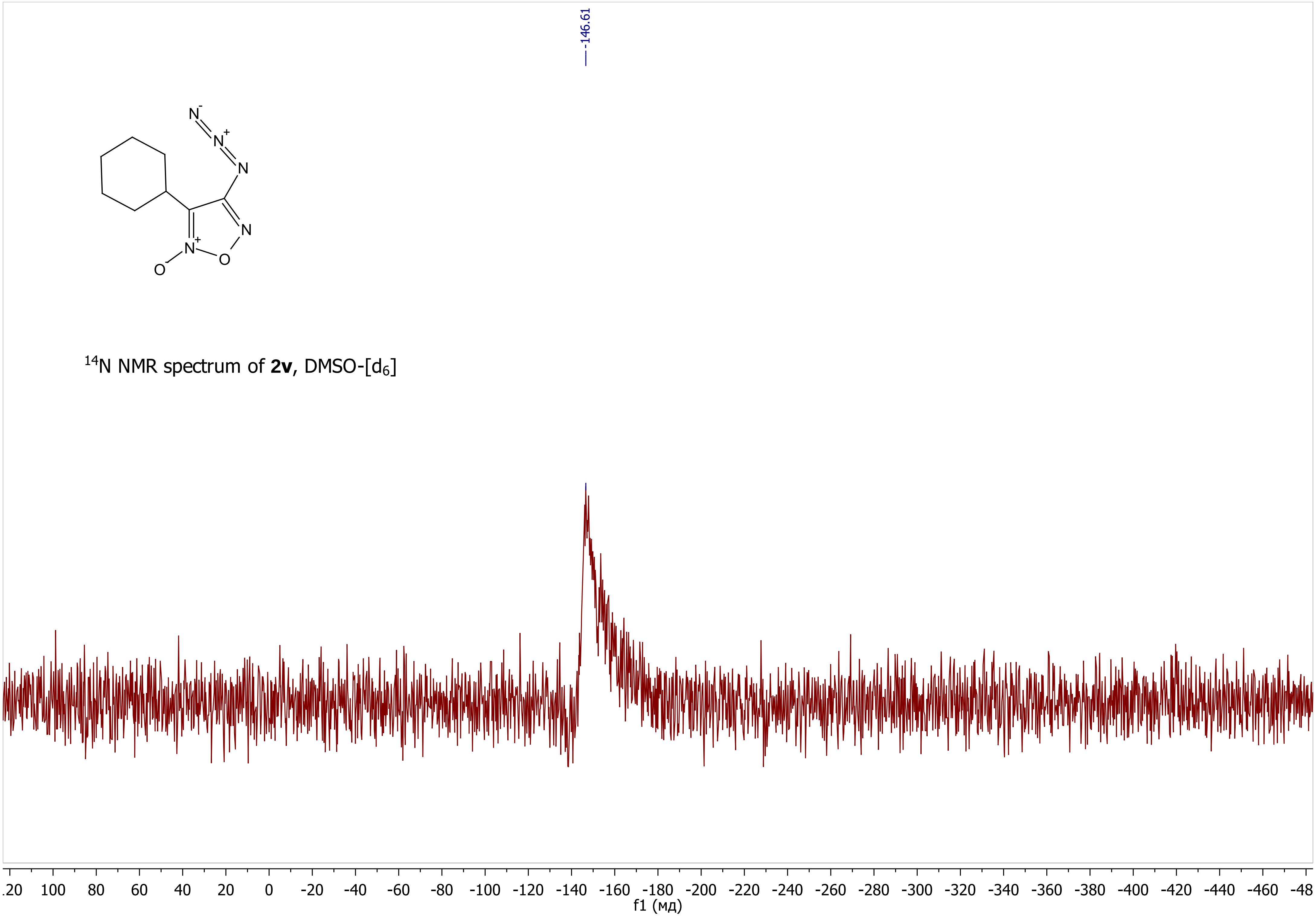

^{14}N NMR spectrum of **2v**, DMSO-[d_6]

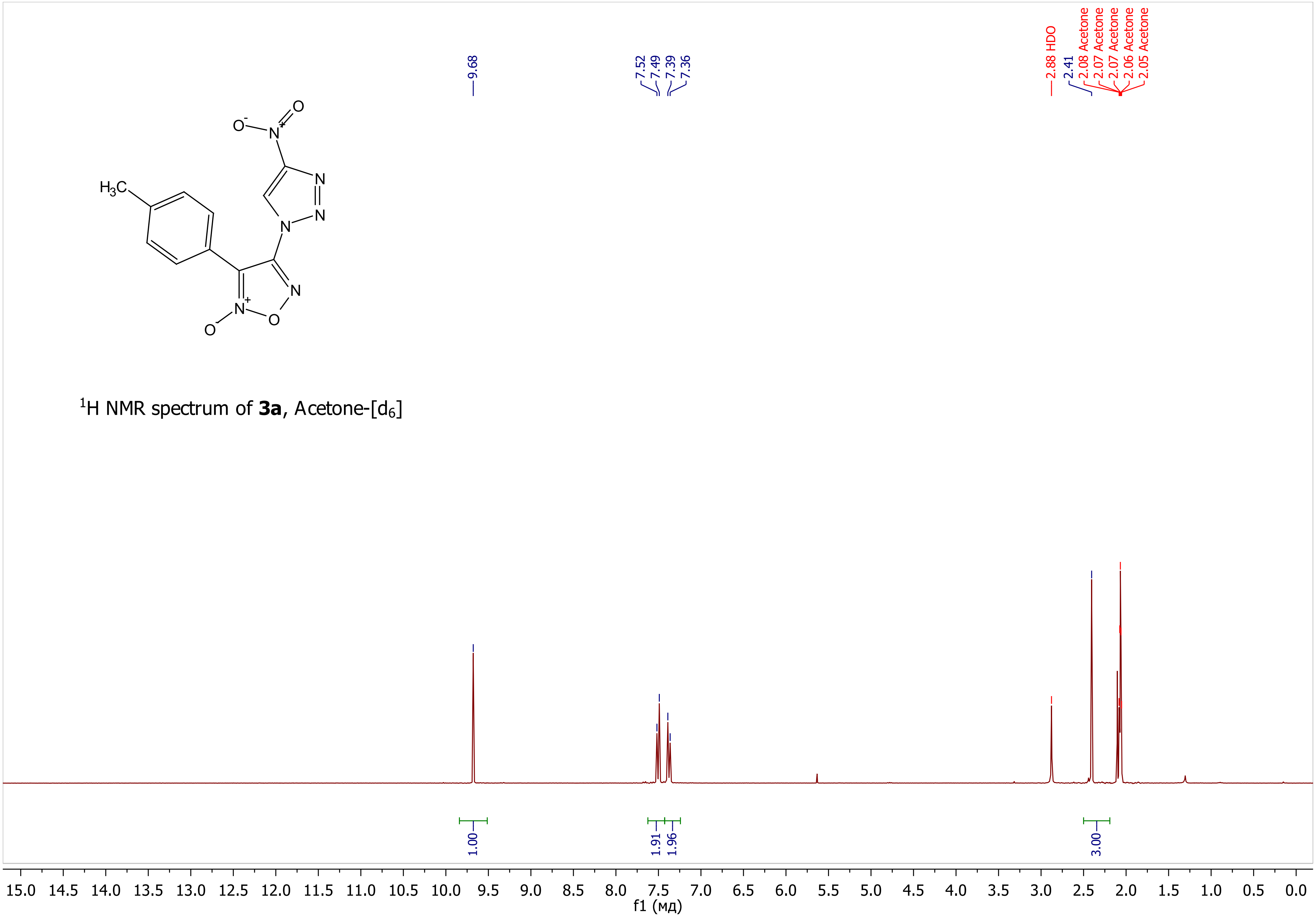

^{1}H NMR spectrum of **3a**, Acetone-[d_6]

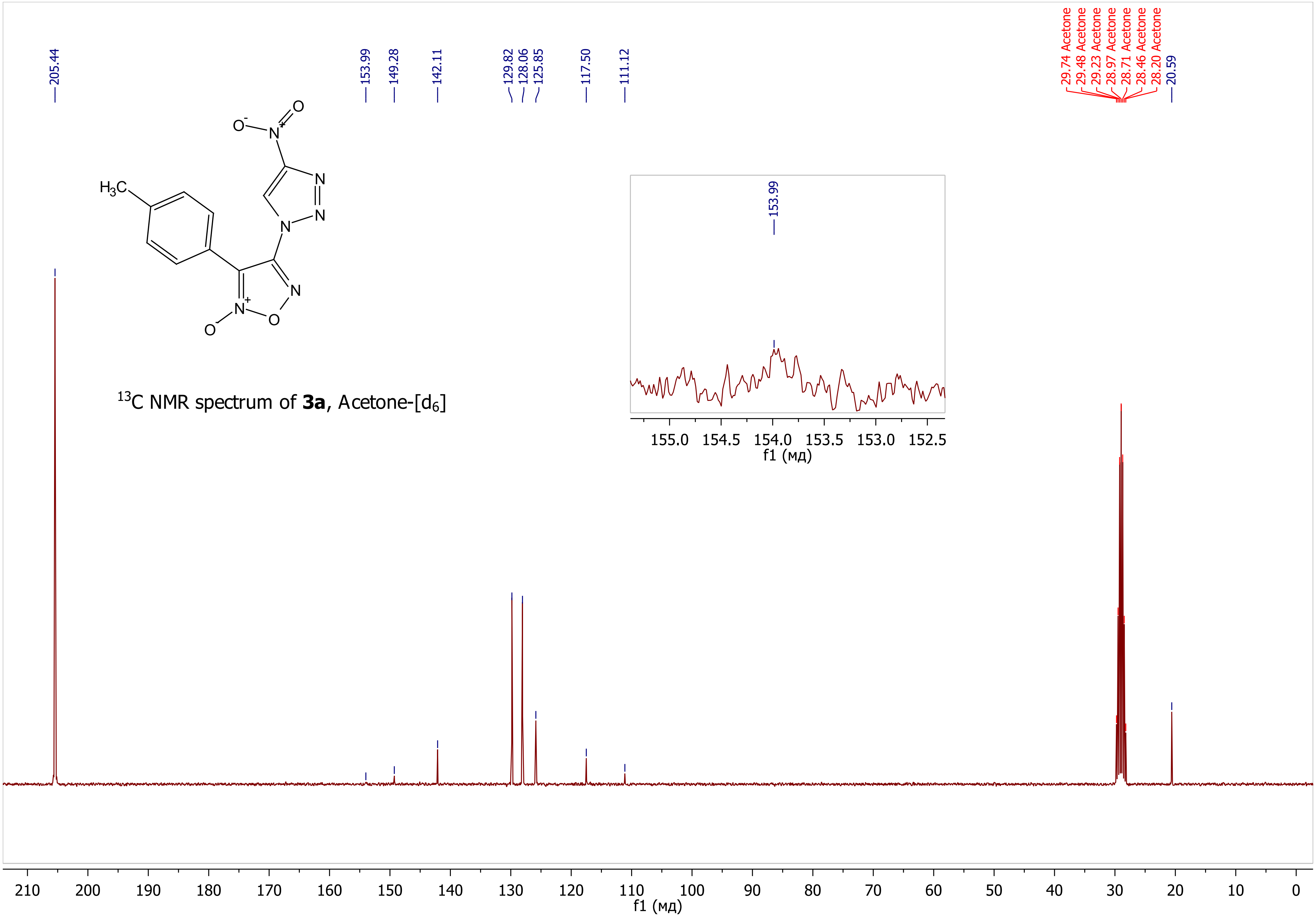

^{13}C NMR spectrum of **3a**, Acetone-[d$_6$]

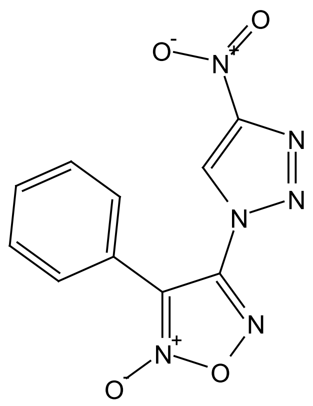

^{1}H NMR spectrum of **3b**, Acetone-[d_6]

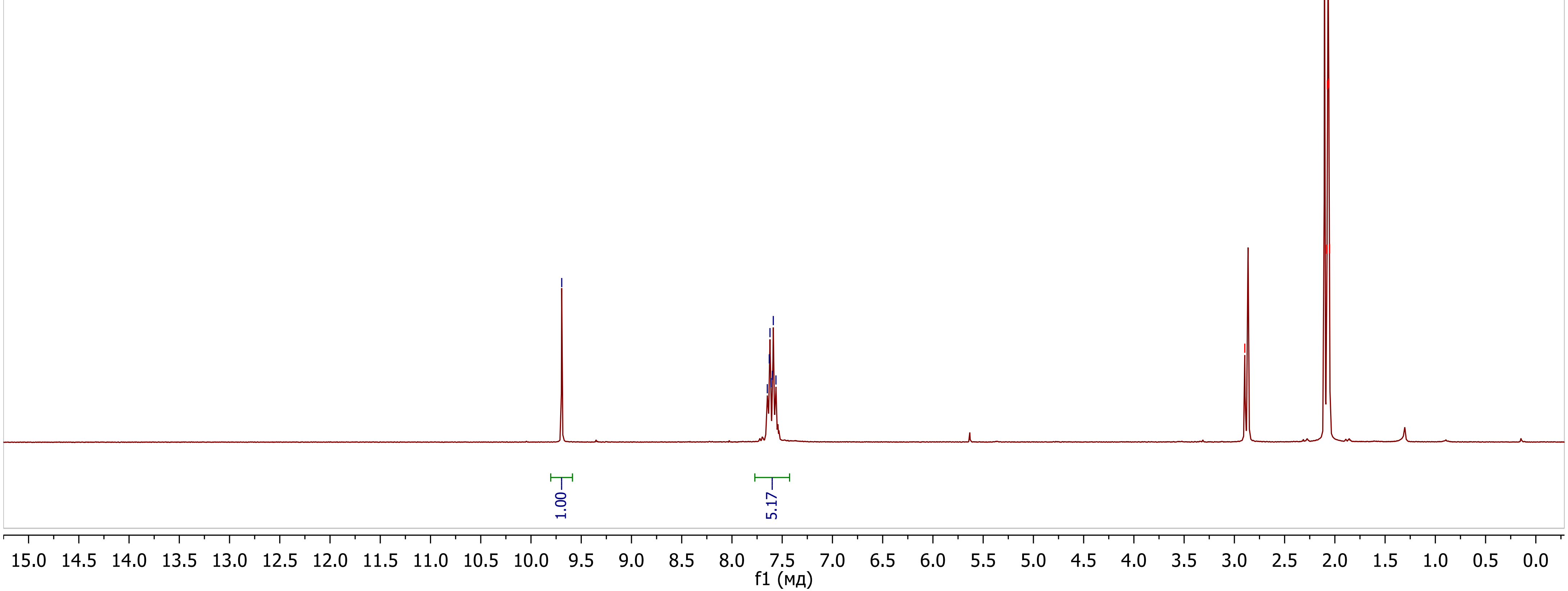

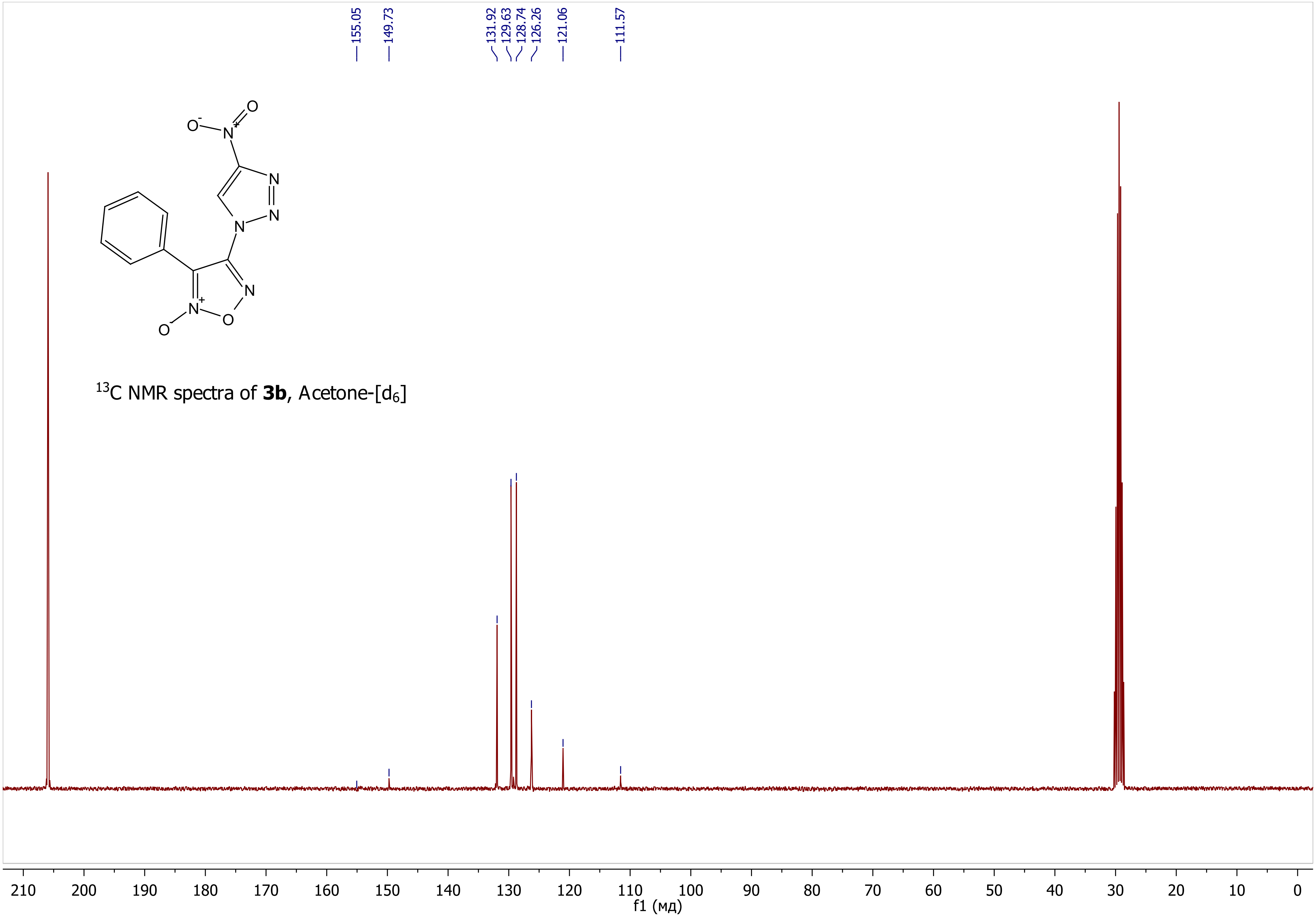

155.05
149.73
131.92
129.63
128.74
126.26
121.06
111.57
^{13}C NMR spectra of **3b**, Acetone-[d$_6$]
210 200 190 180 170 160 150 140 130 120 110 100 90 80 70 60 50 40 30 20 10 0
f1 (мд)

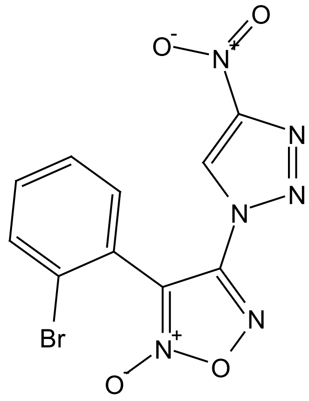

^{1}H NMR spectrum of **3c**, Acetone-[d$_6$]

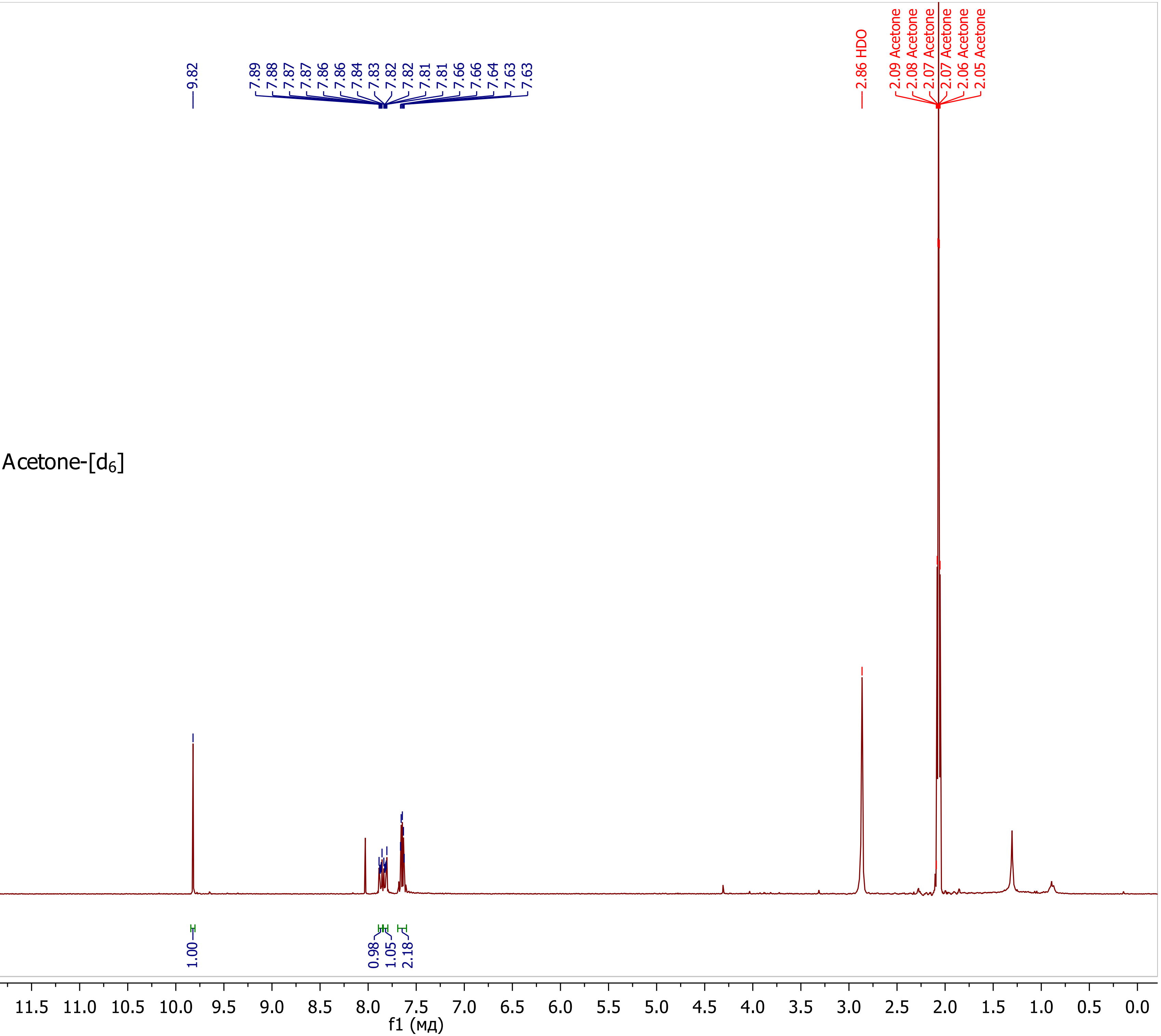

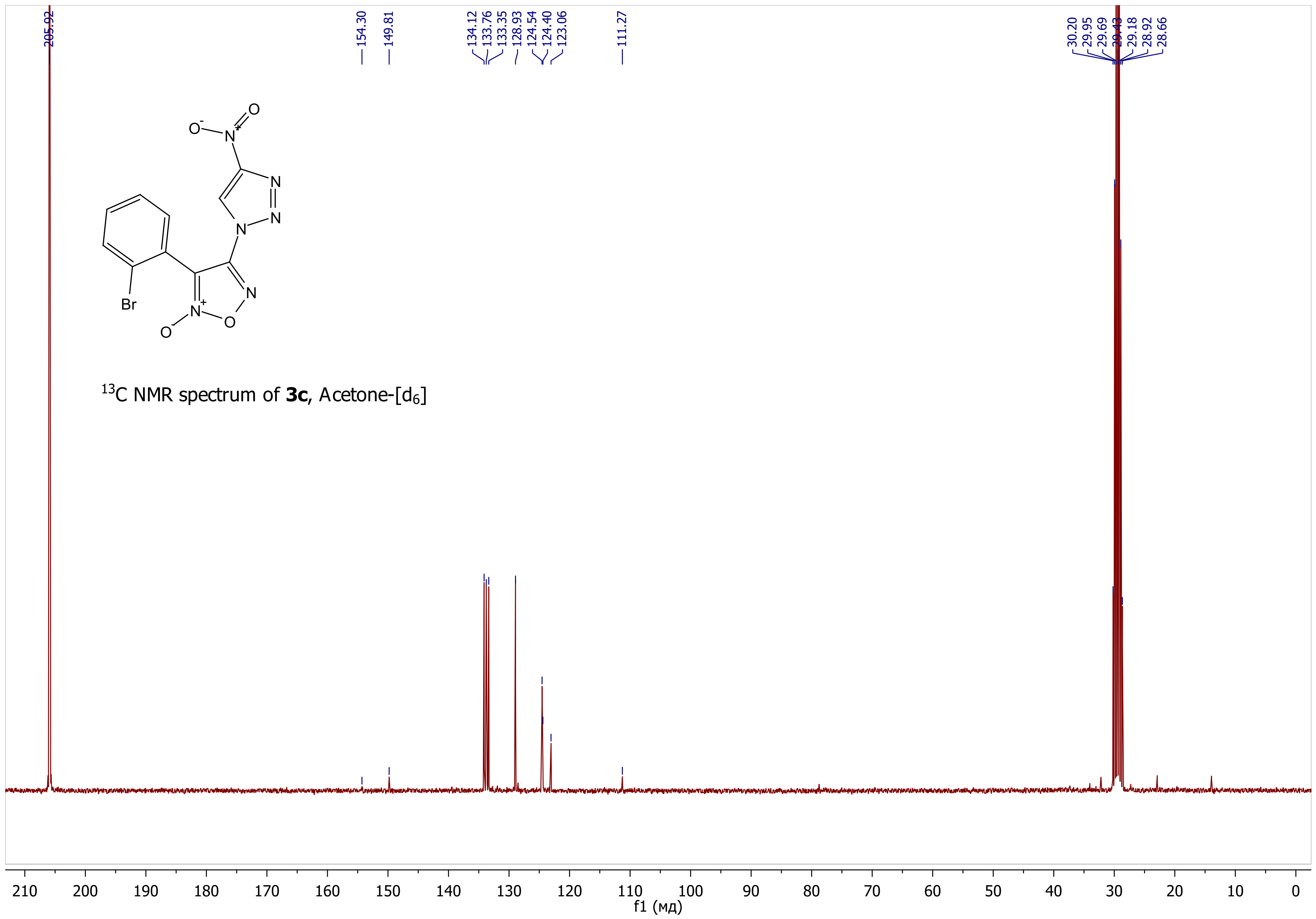

^{13}C NMR spectrum of **3c**, Acetone-[d_6]

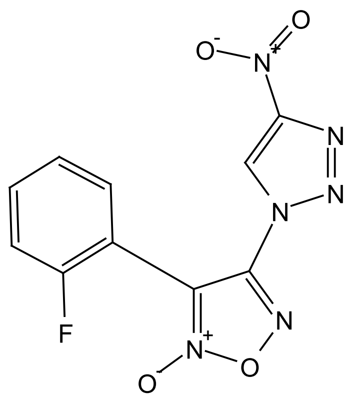

^{1}H NMR spectrum of **3d**, Acetone[d_6]

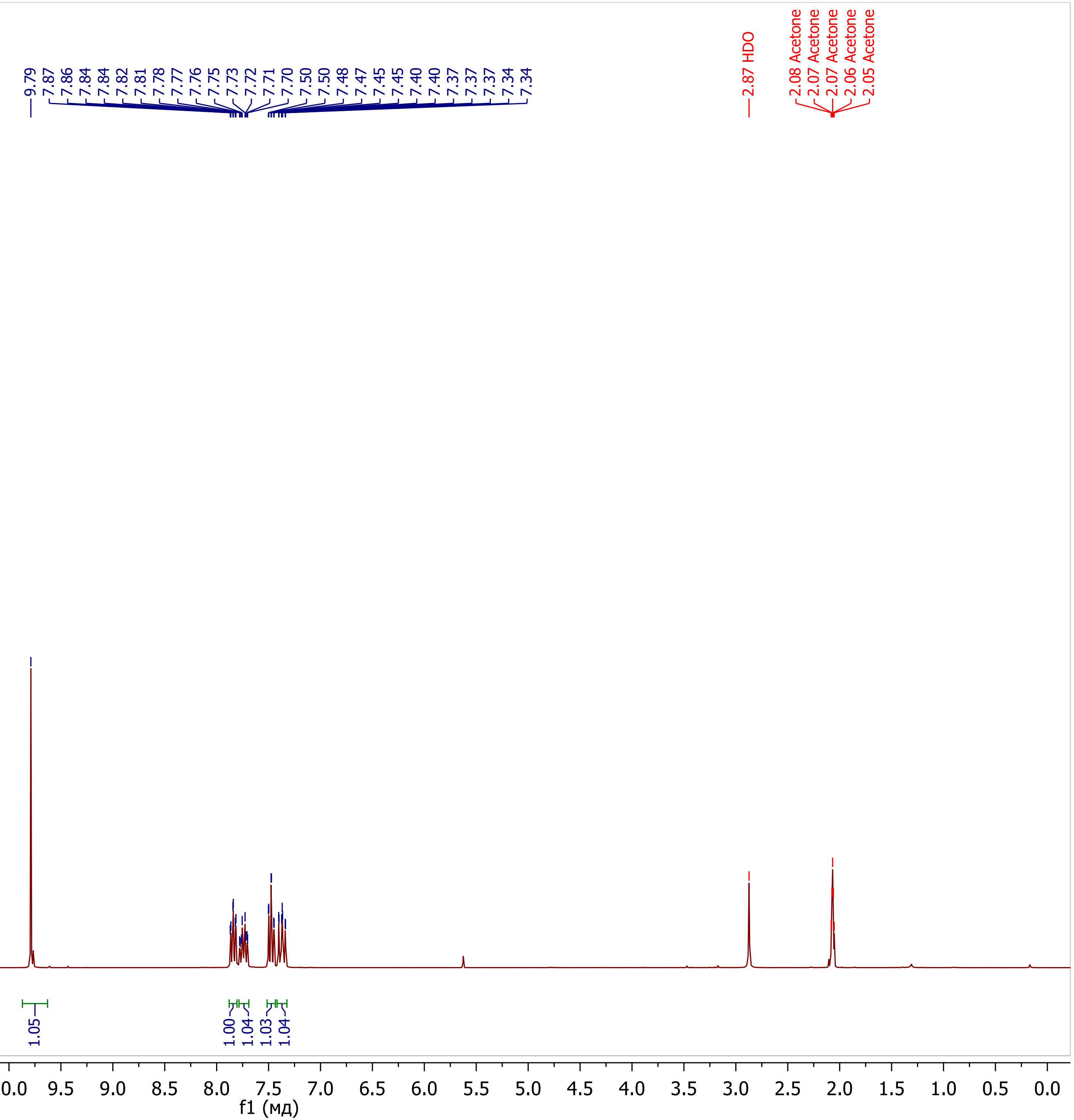

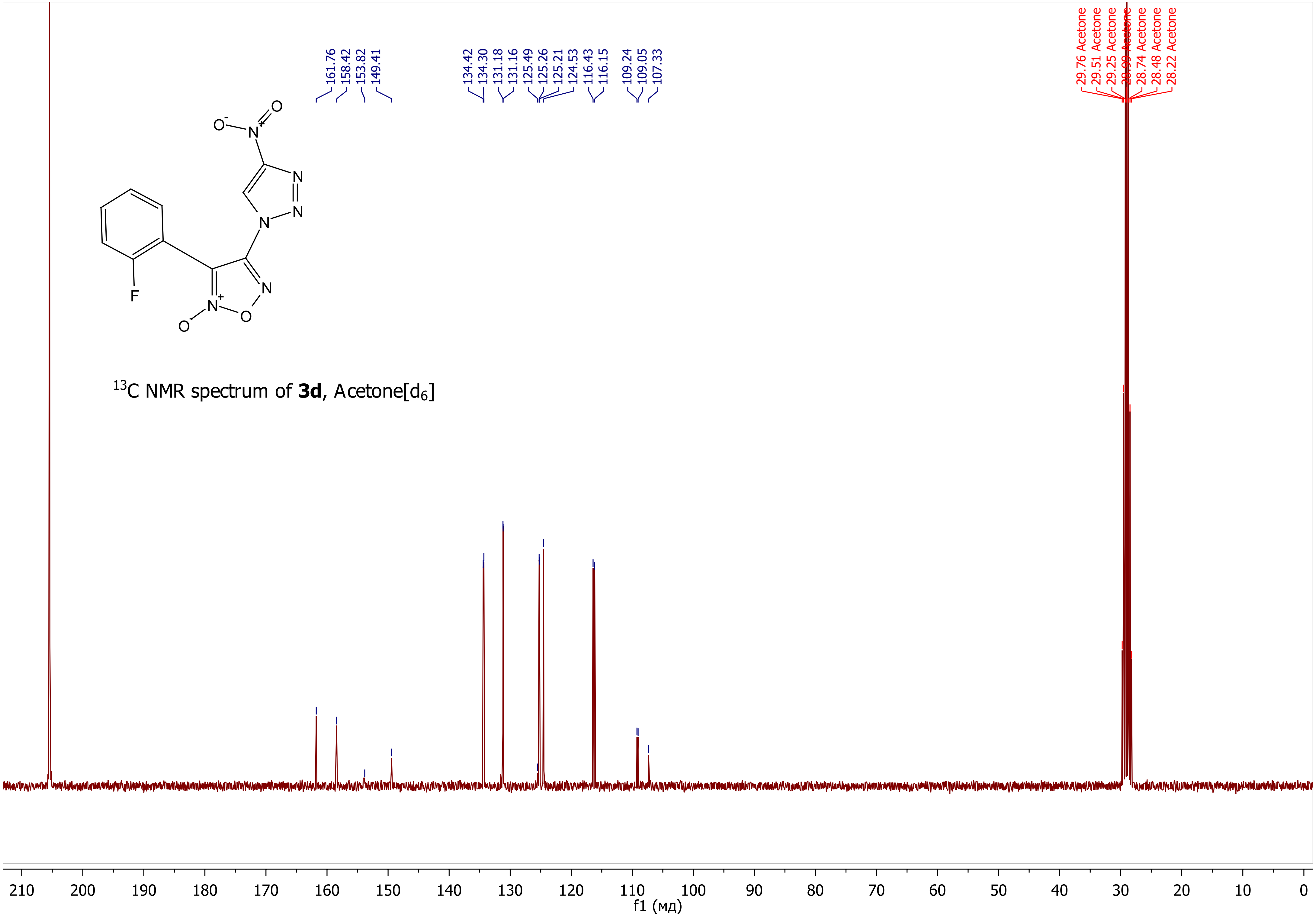

^{13}C NMR spectrum of **3d**, Acetone[d$_6$]

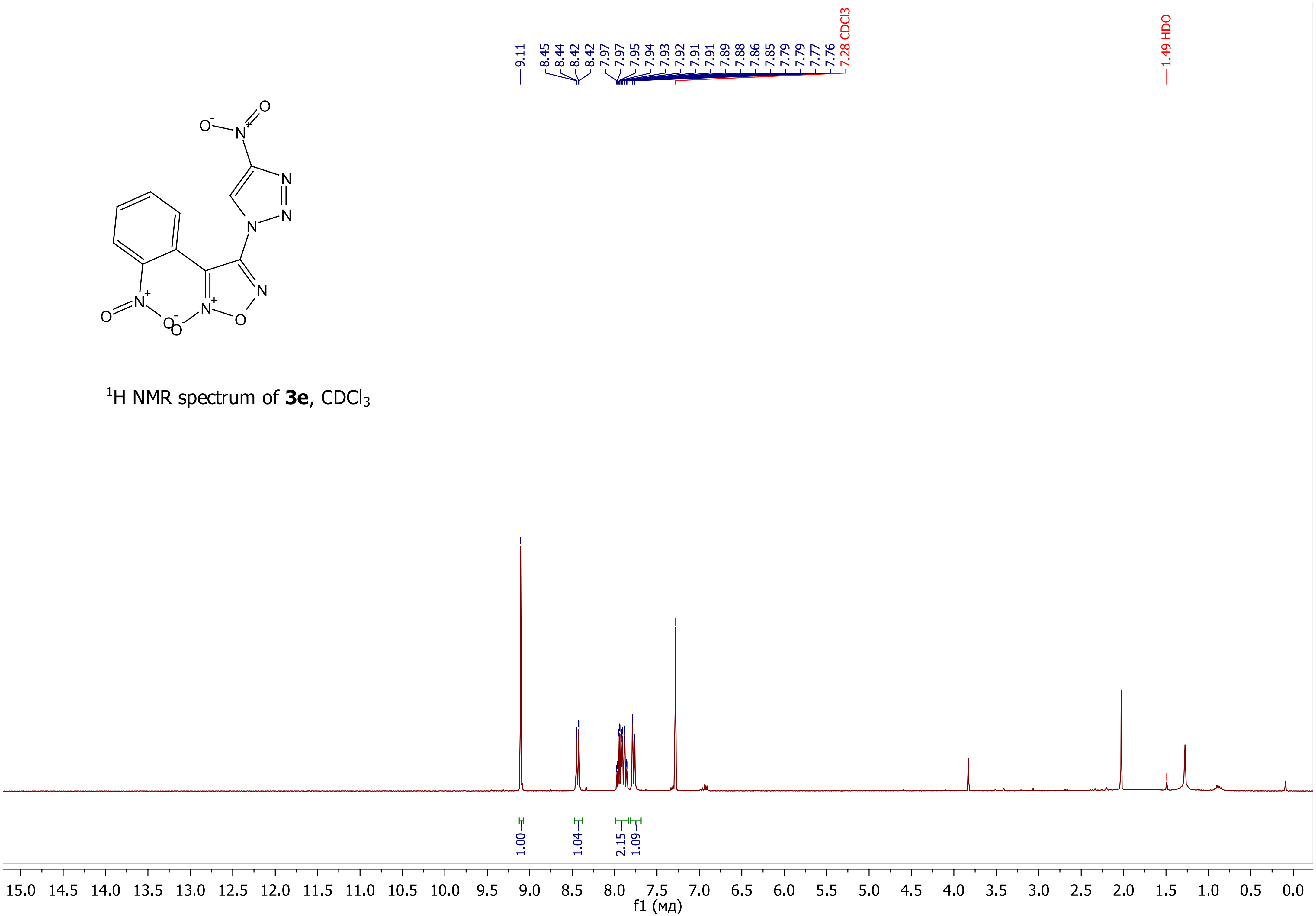

^{1}H NMR spectrum of **3e**, $CDCl_3$

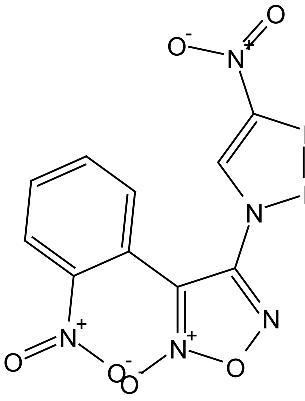

^{13}C NMR spectrum of **3e**, $CDCl_3$

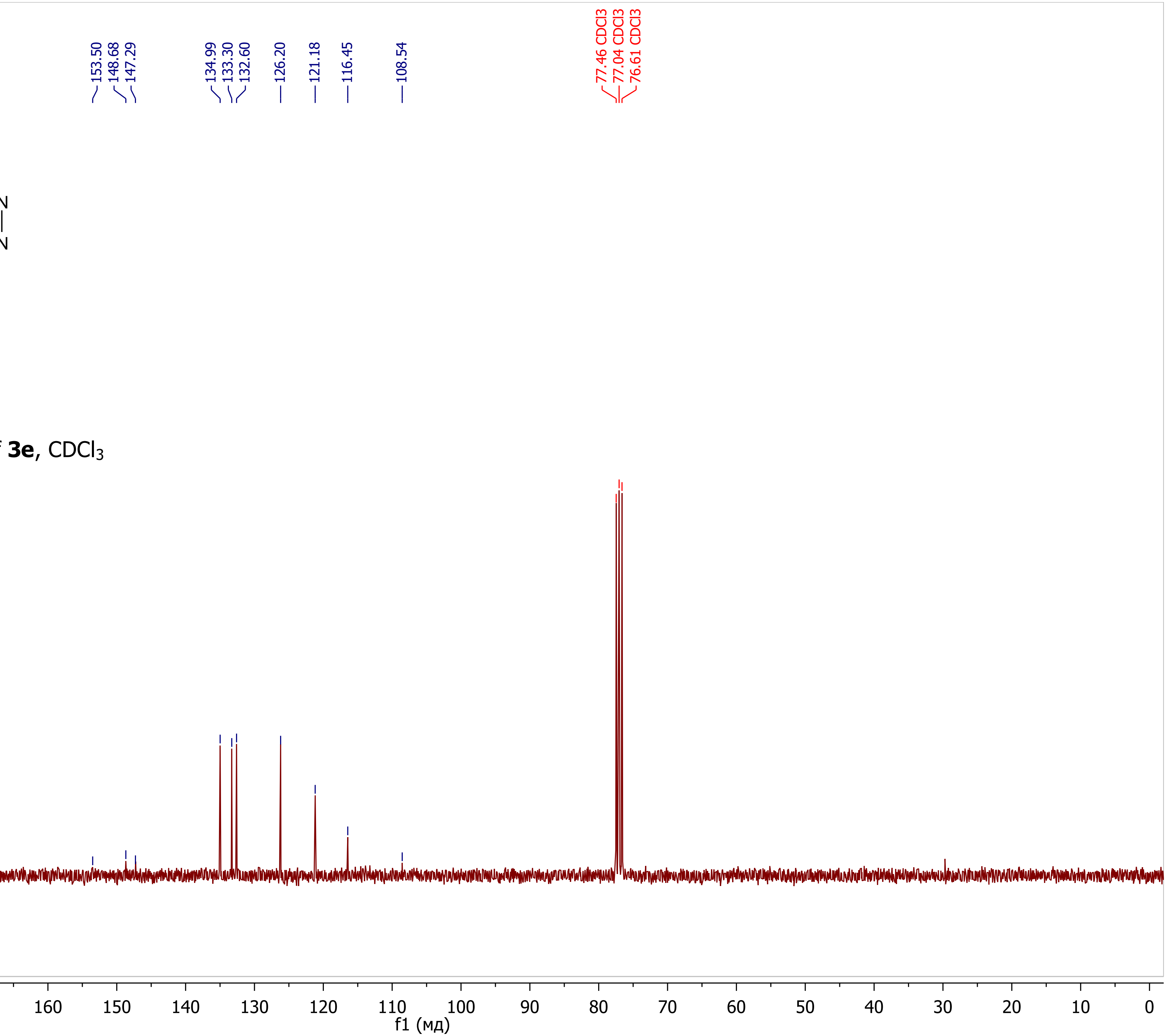

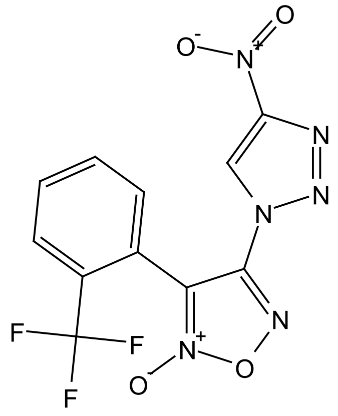

^{1}H NMR spectrum of **3f**, $CDCl_3$

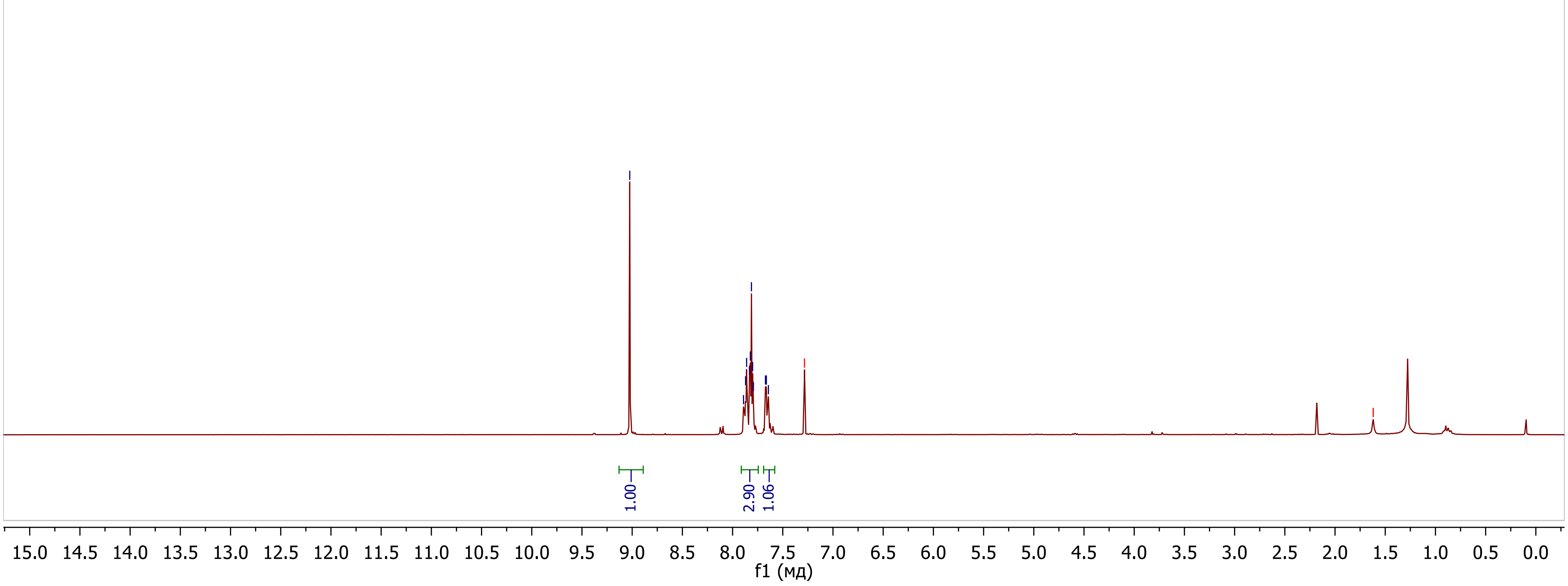

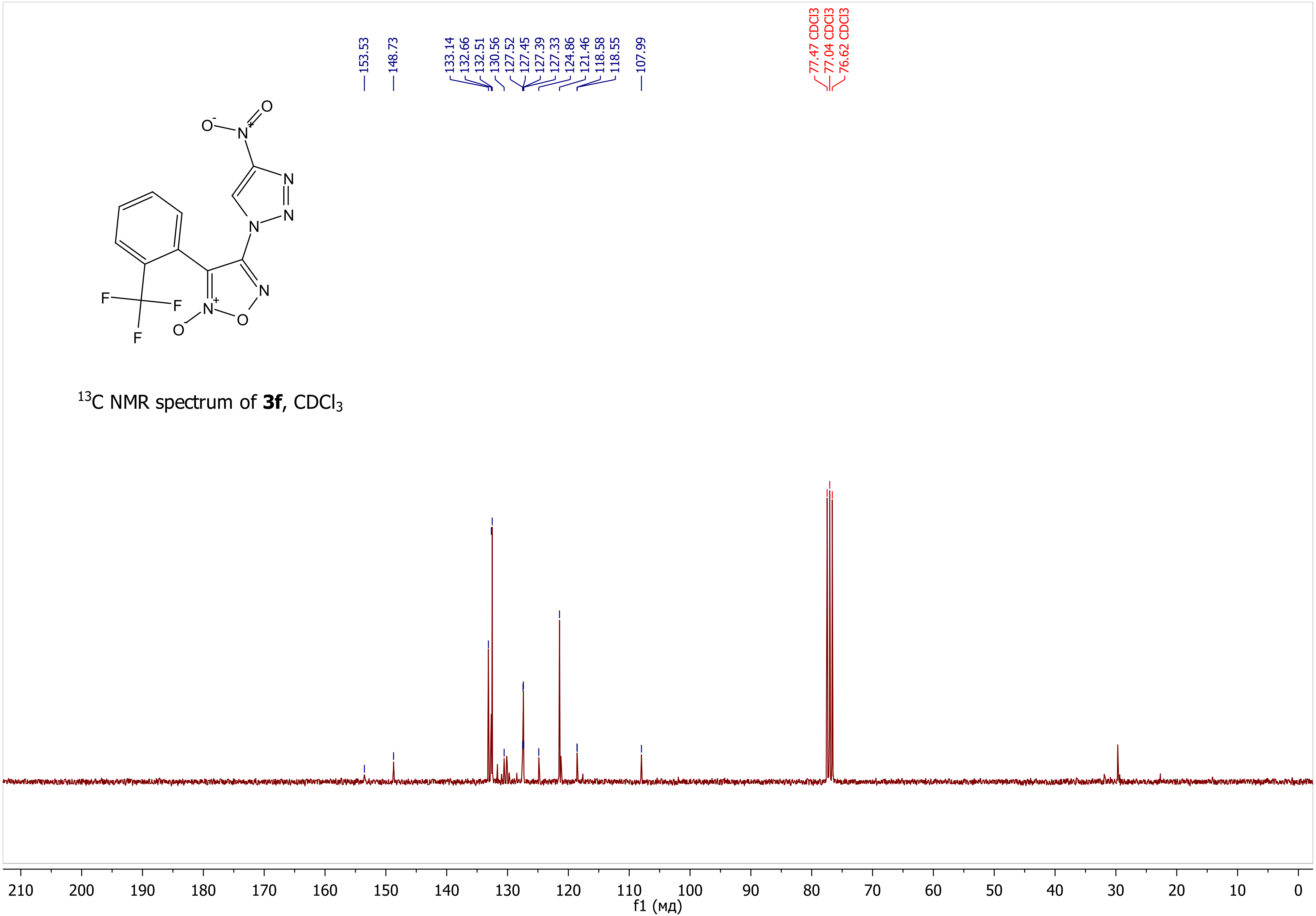

^{13}C NMR spectrum of **3f**, $CDCl_3$

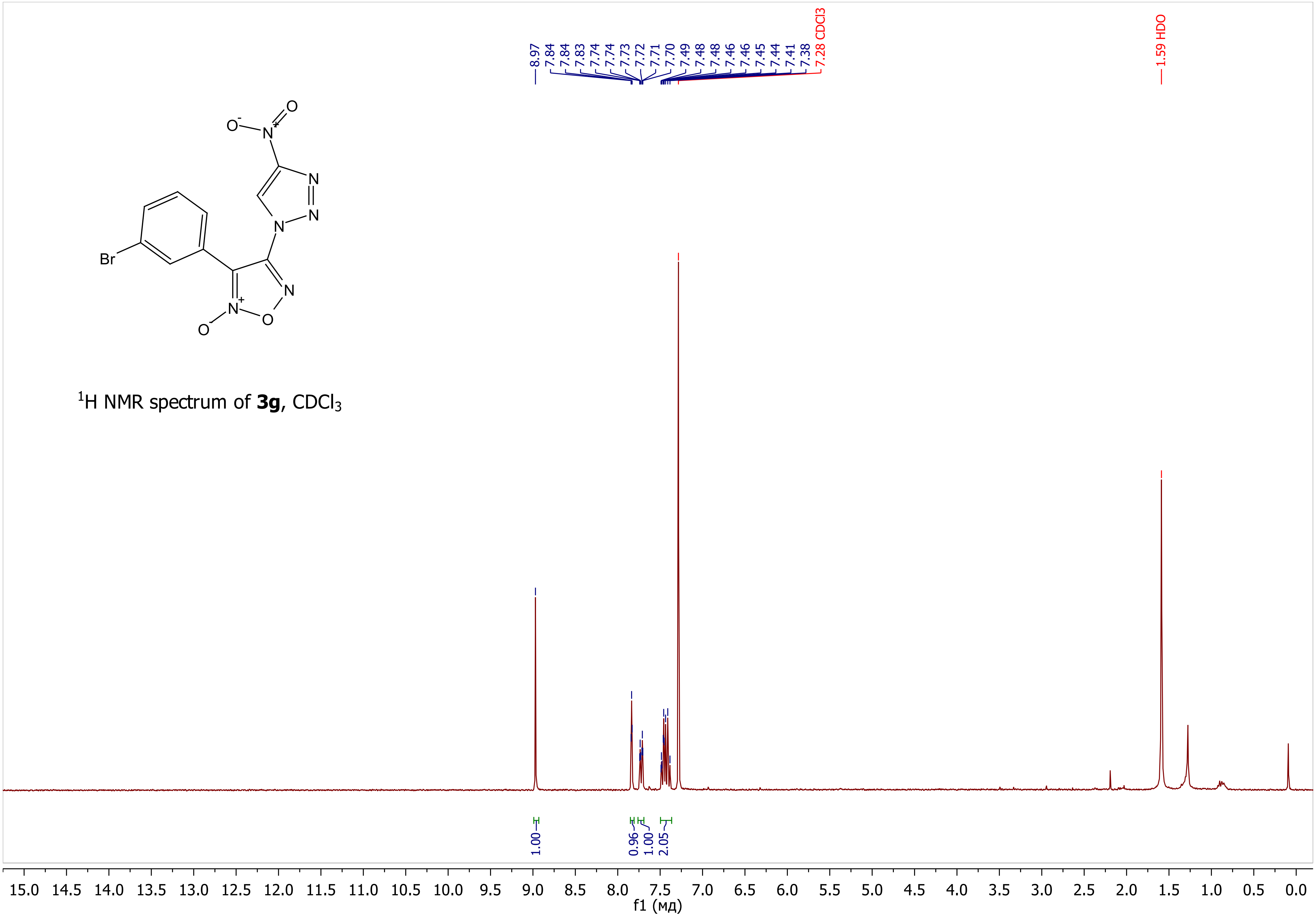

^{1}H NMR spectrum of **3g**, $CDCl_3$

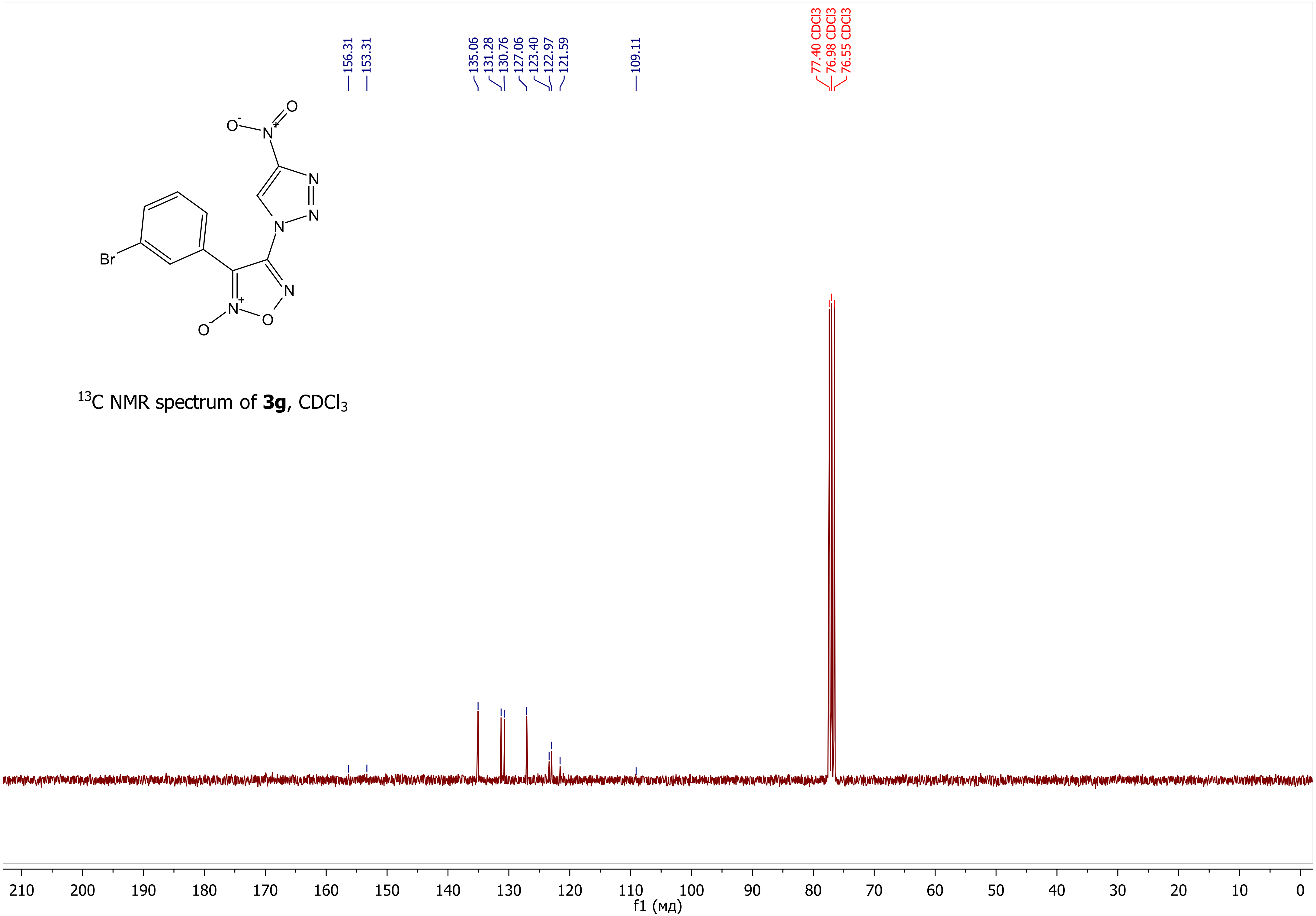

^{13}C NMR spectrum of **3g**, $CDCl_3$

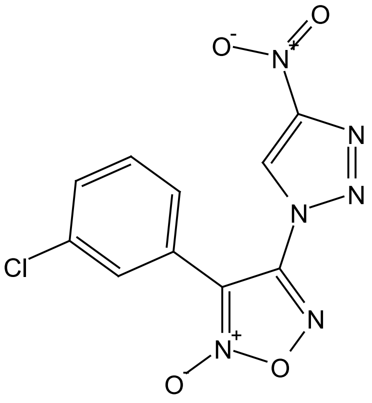

^{1}H NMR spectrum of **3h**, $CDCl_3$

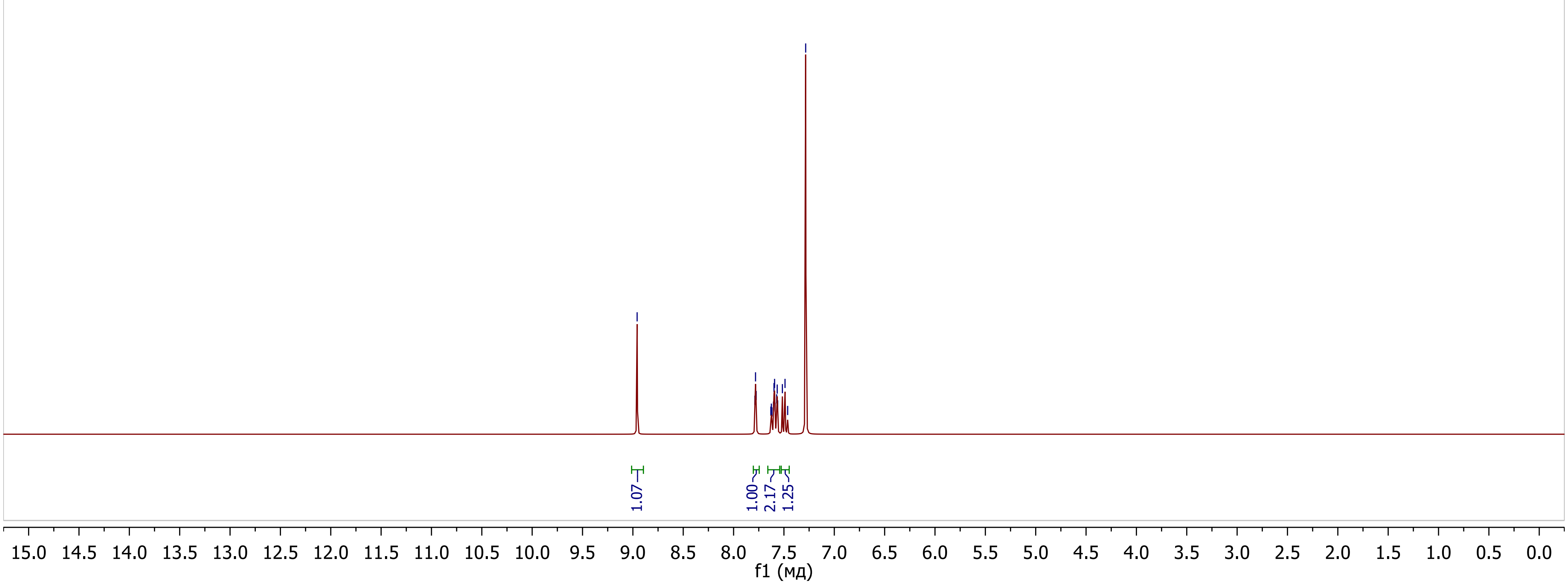

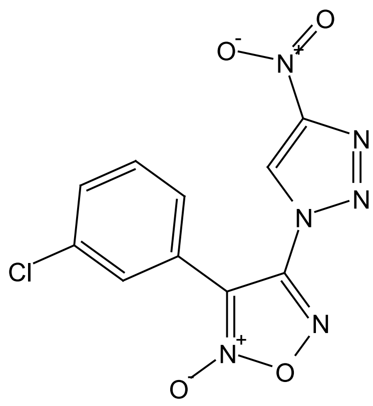

^{13}C NMR spectrum of **3h**, $CDCl_3$

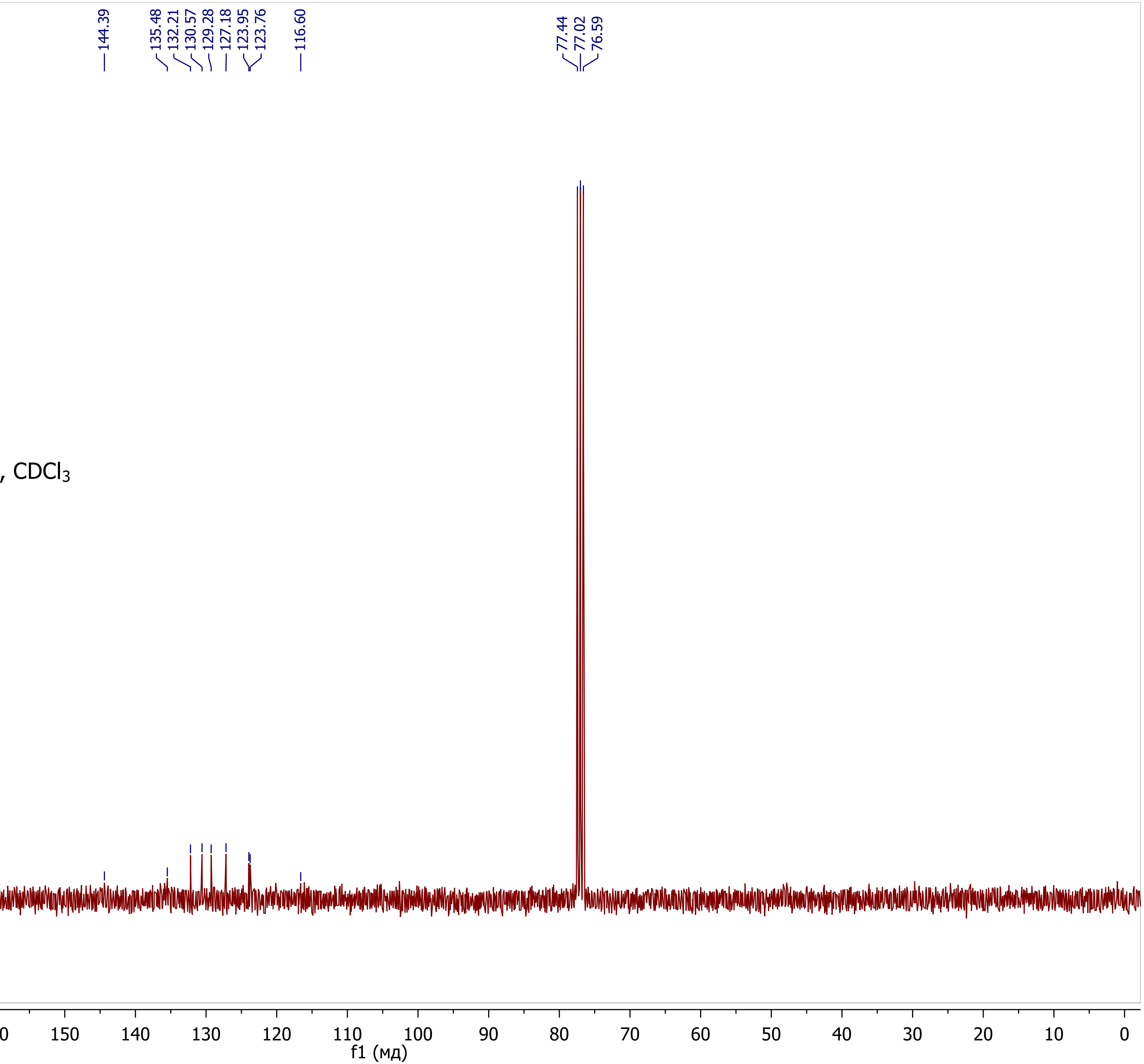

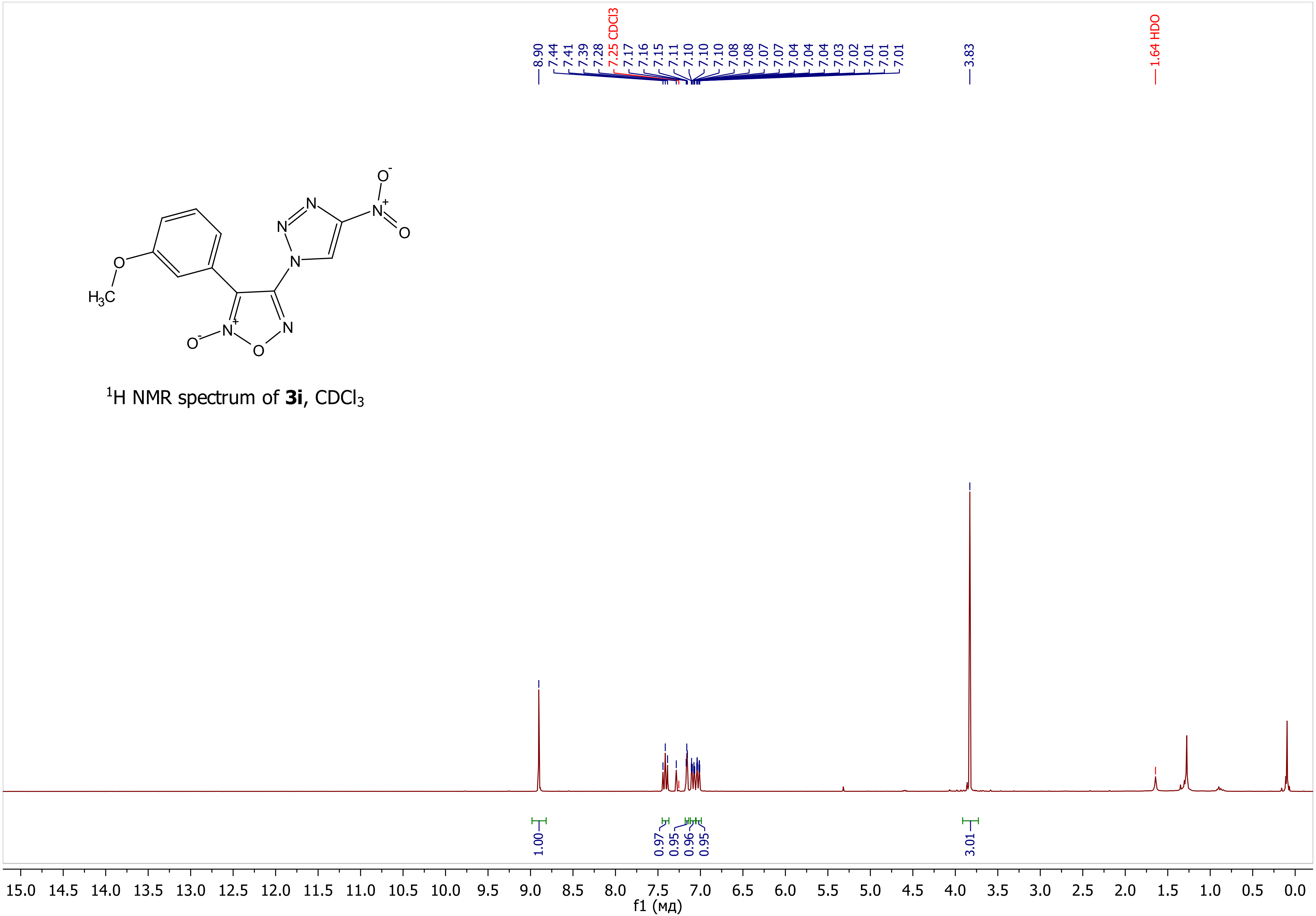

^{1}H NMR spectrum of **3i**, $CDCl_3$

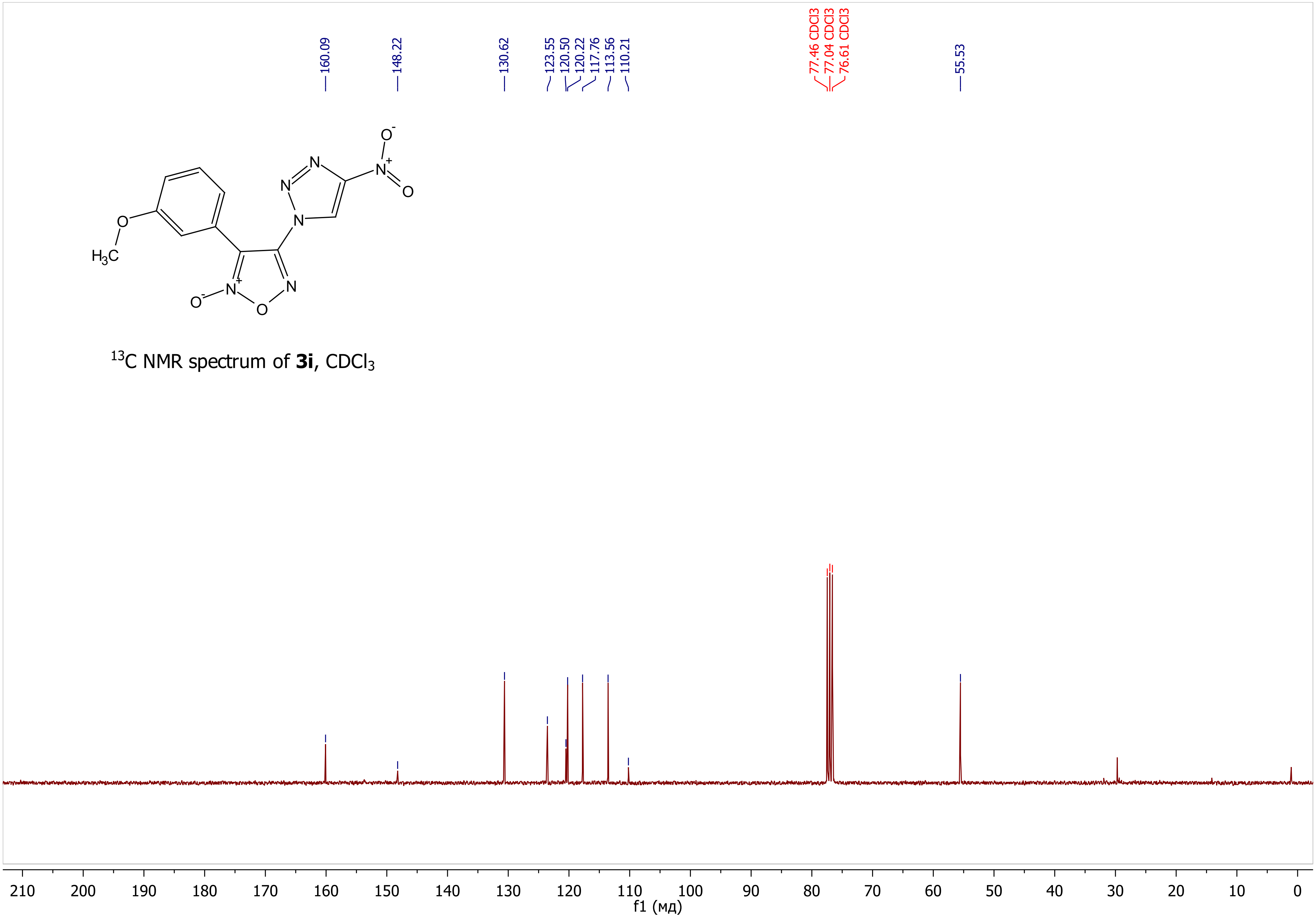

^{13}C NMR spectrum of **3i**, $CDCl_3$

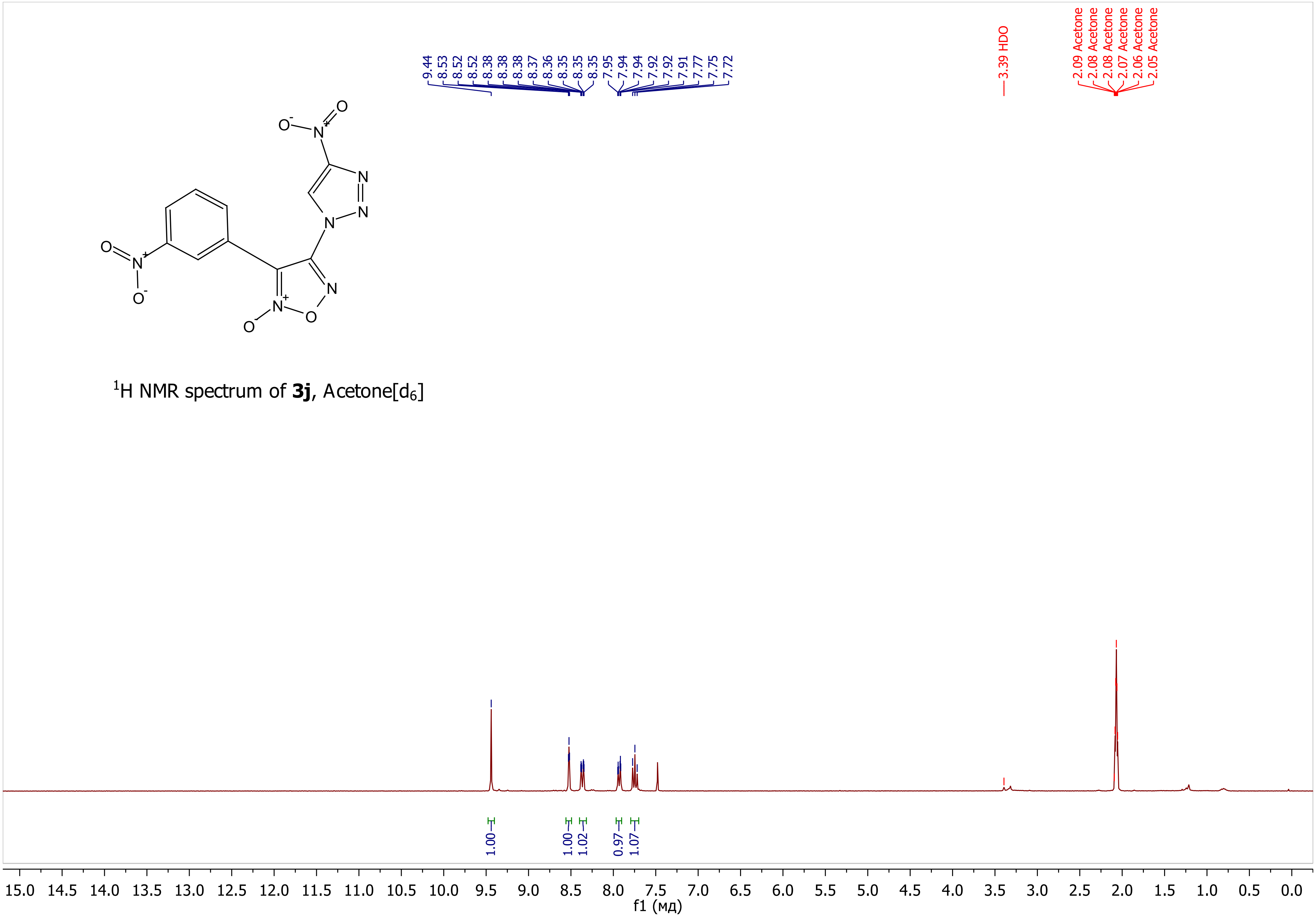

^{1}H NMR spectrum of **3j**, Acetone[d$_6$]

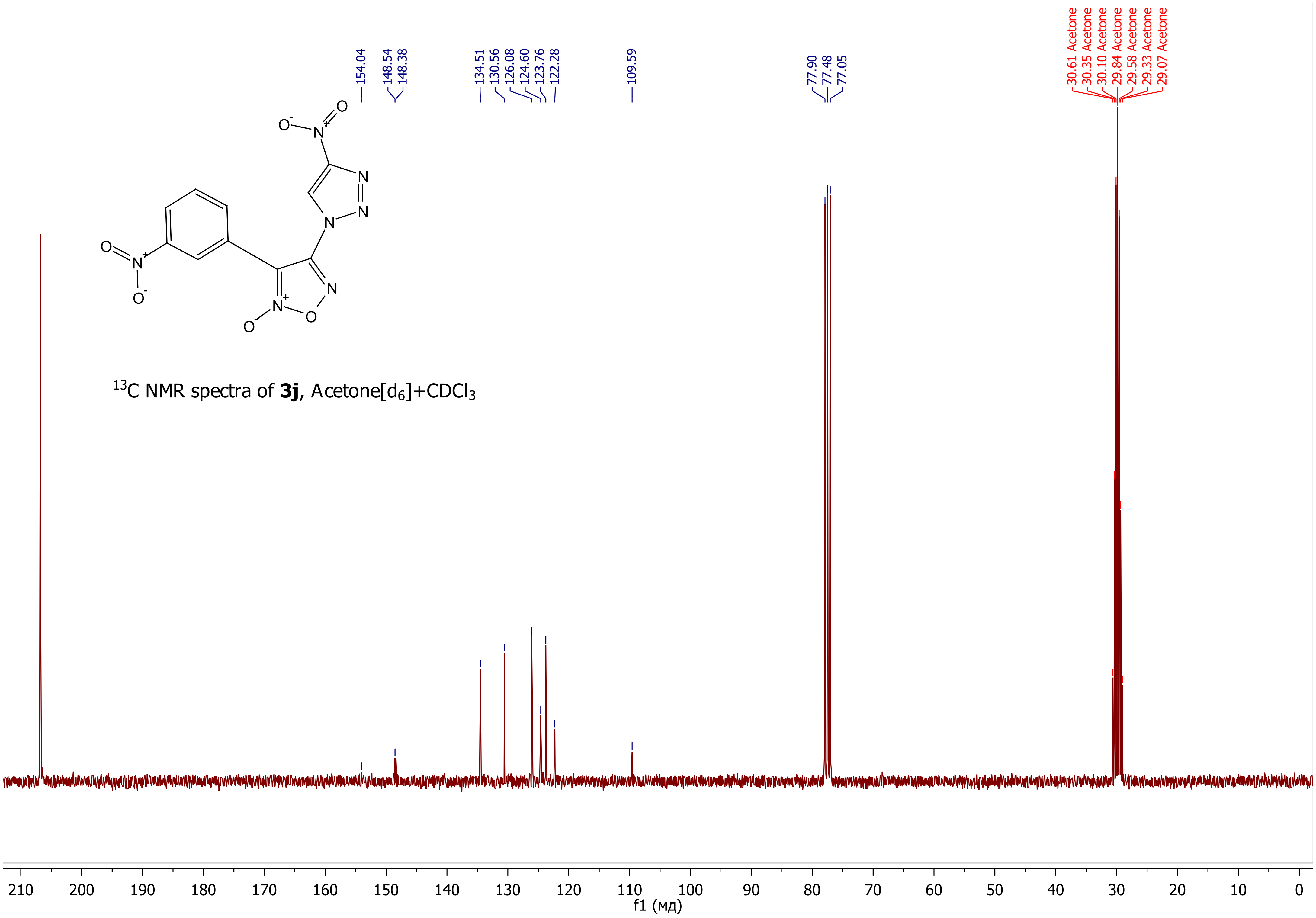

^{13}C NMR spectra of **3j**, Acetone[d_6]+$CDCl_3$

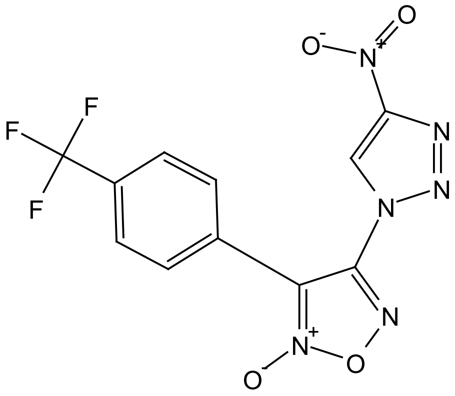

^{13}C NMR spectra of **3k**, $CDCl_3$

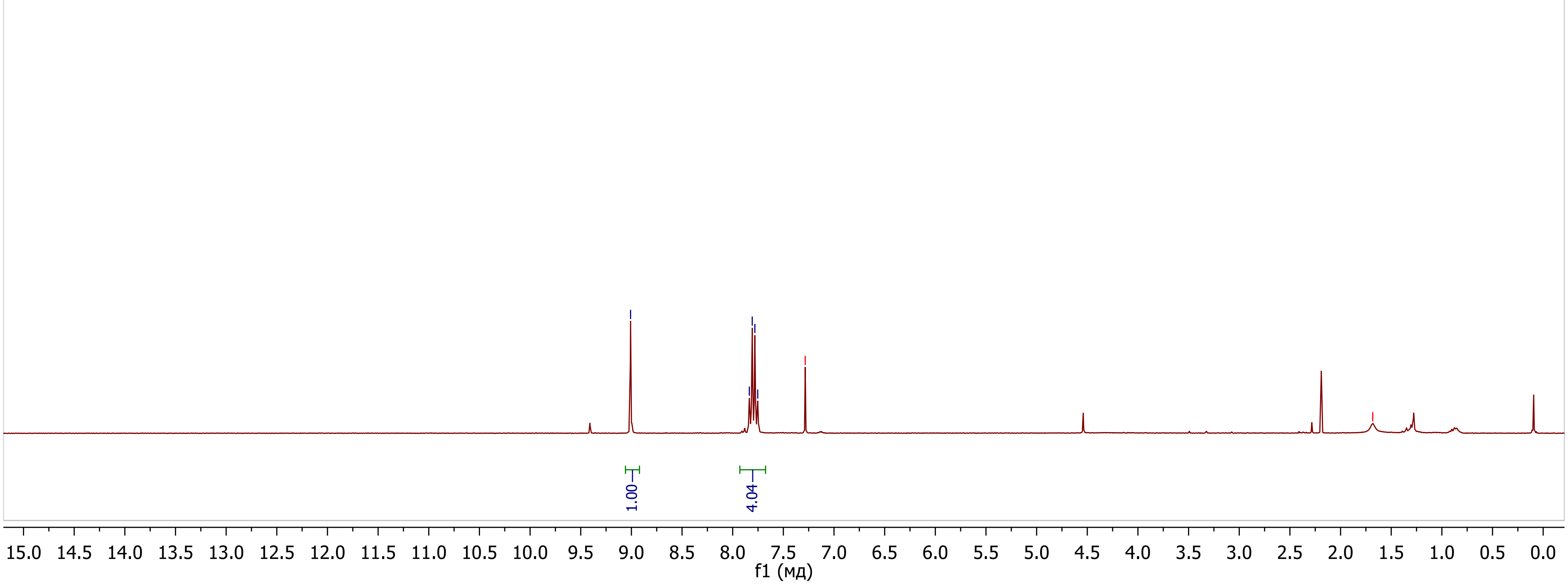

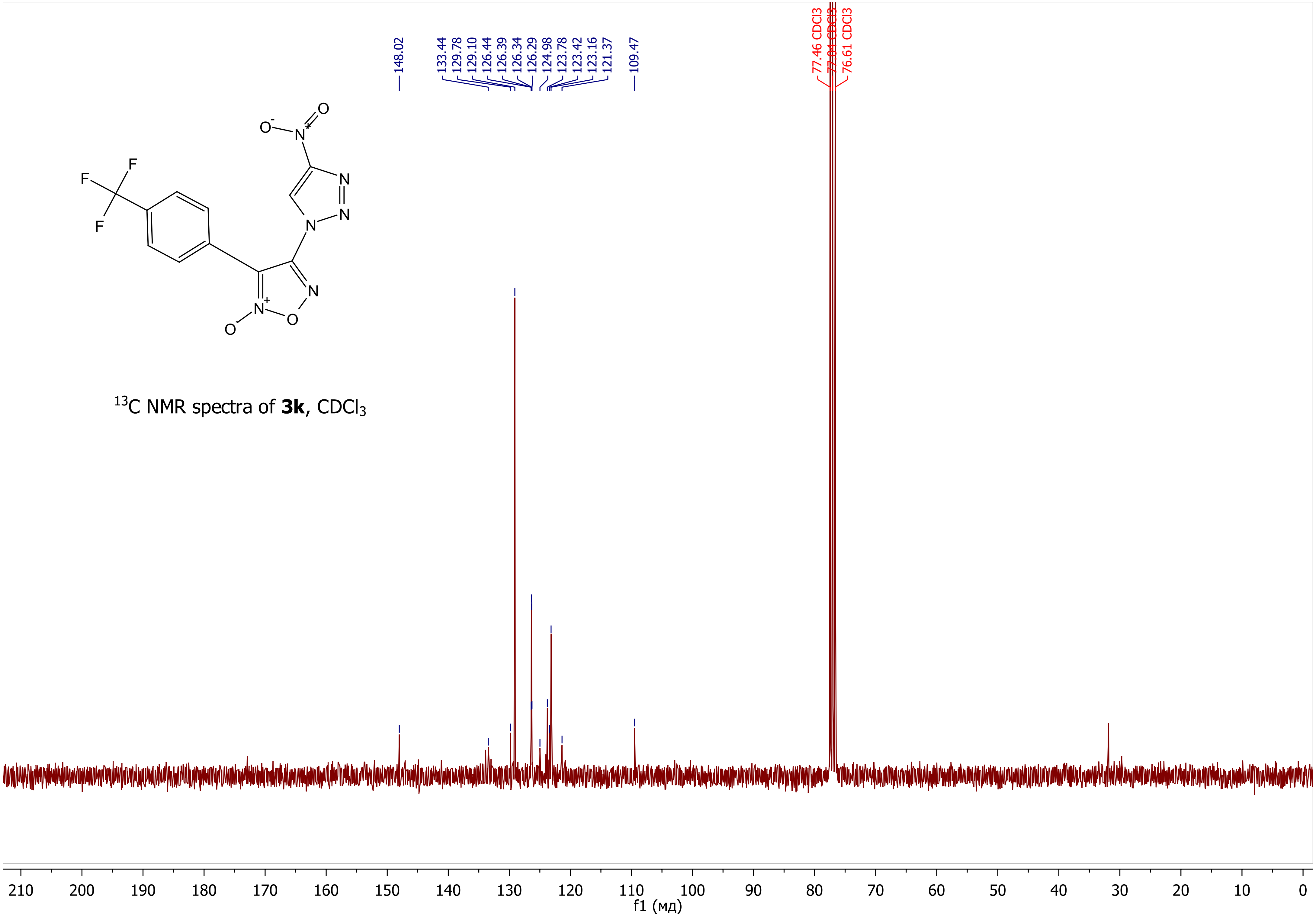

^{13}C NMR spectra of **3k**, $CDCl_3$

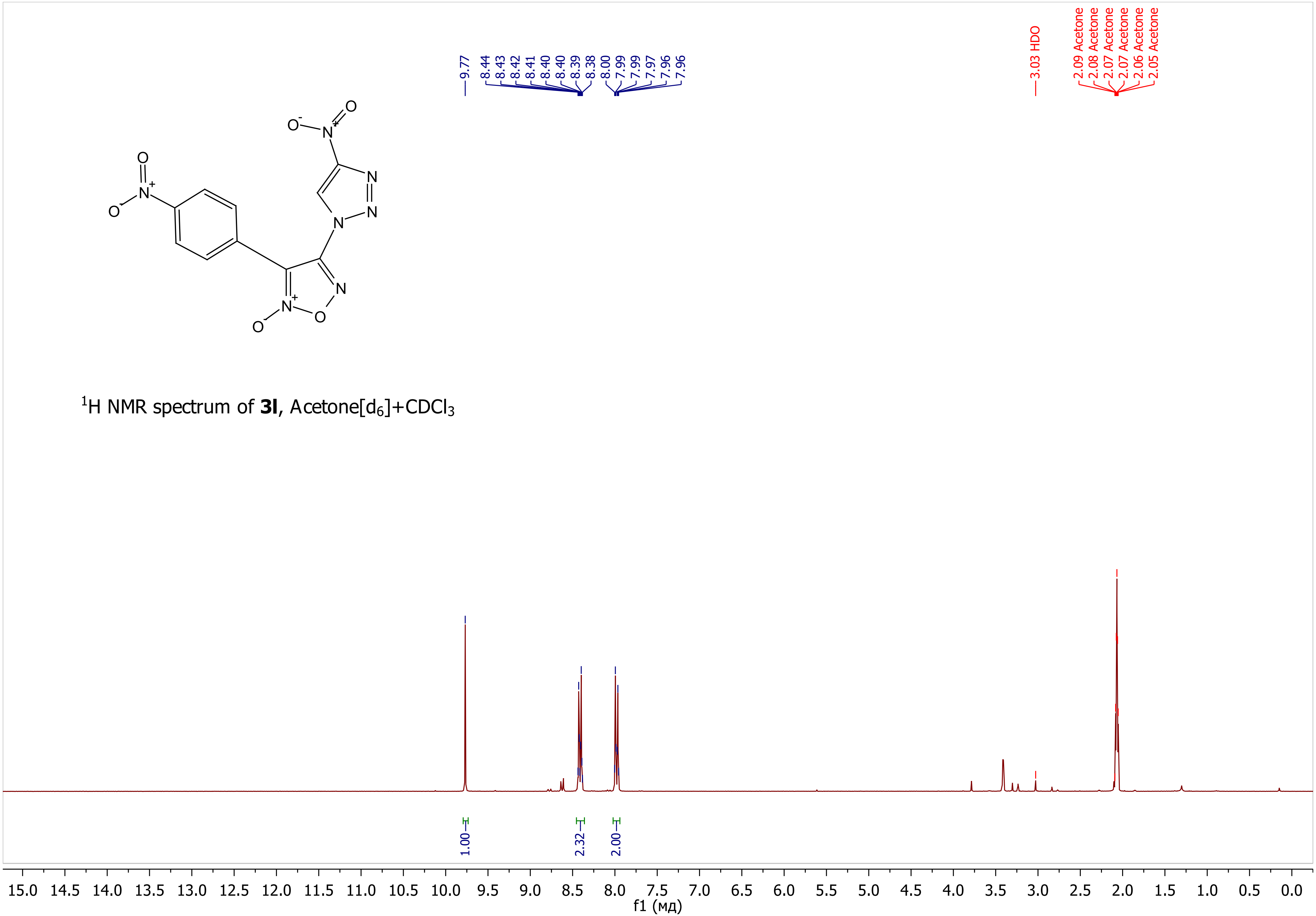

^{1}H NMR spectrum of **3l**, Acetone[d$_6$]+CDCl$_3$

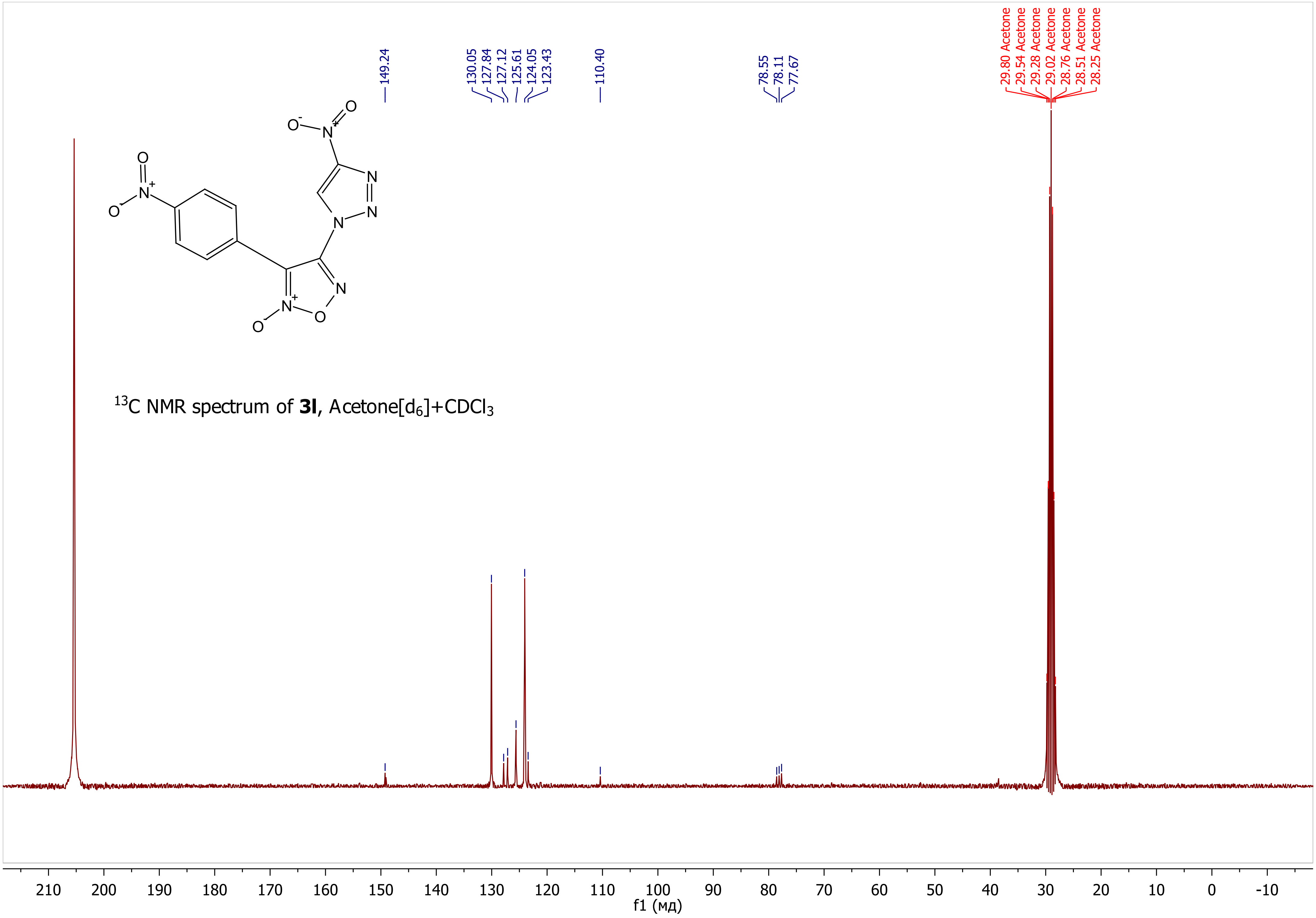
149.24
130.05
127.84
127.12
125.61
124.05
123.43
110.40
78.55
78.11
77.67
29.80 Acetone
29.54 Acetone
29.28 Acetone
29.02 Acetone
28.76 Acetone
28.51 Acetone
28.25 Acetone
^{13}C NMR spectrum of **3l**, Acetone[d$_6$]+CDCl$_3$
210 200 190 180 170 160 150 140 130 120 110 100 90 80 70 60 50 40 30 20 10 0 -10
f1 (мд)

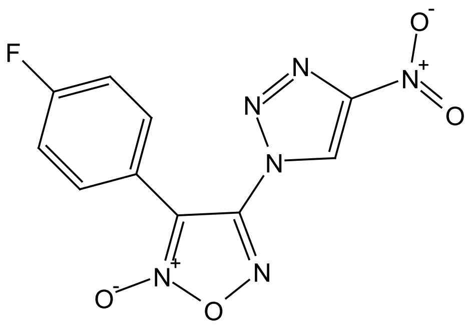

^{1}H NMR spectrum of **3m**, $CDCl_3$

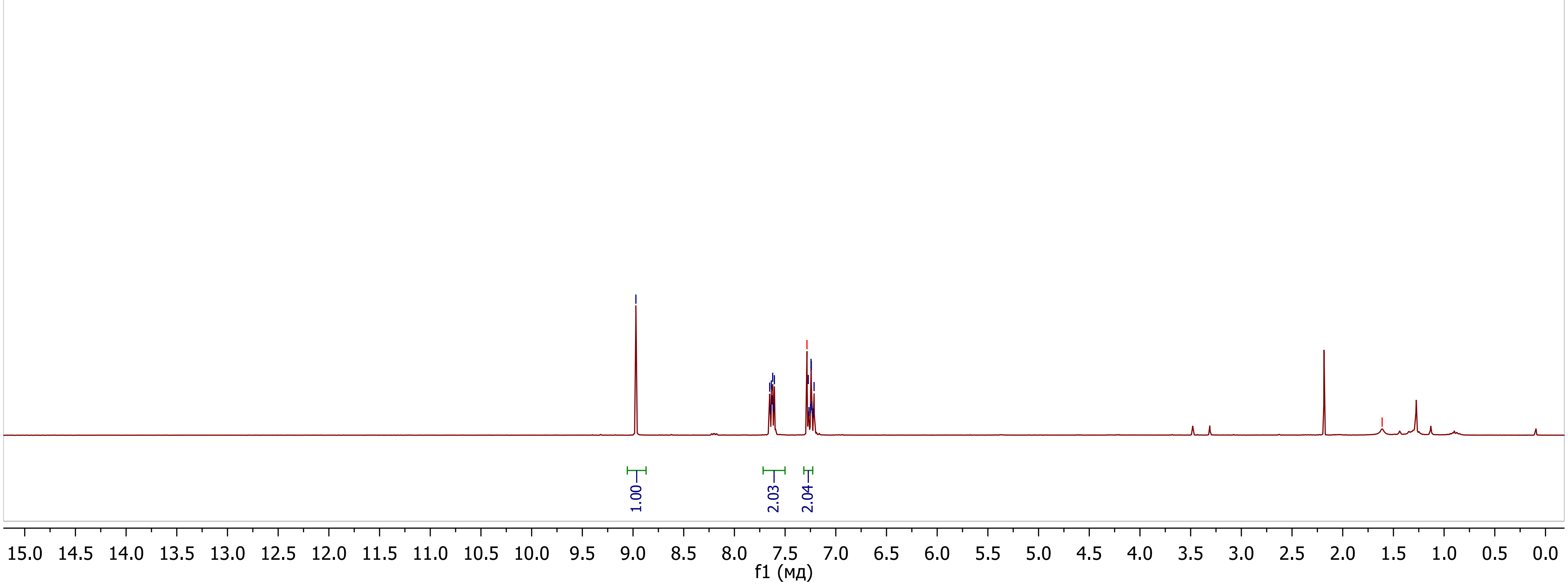

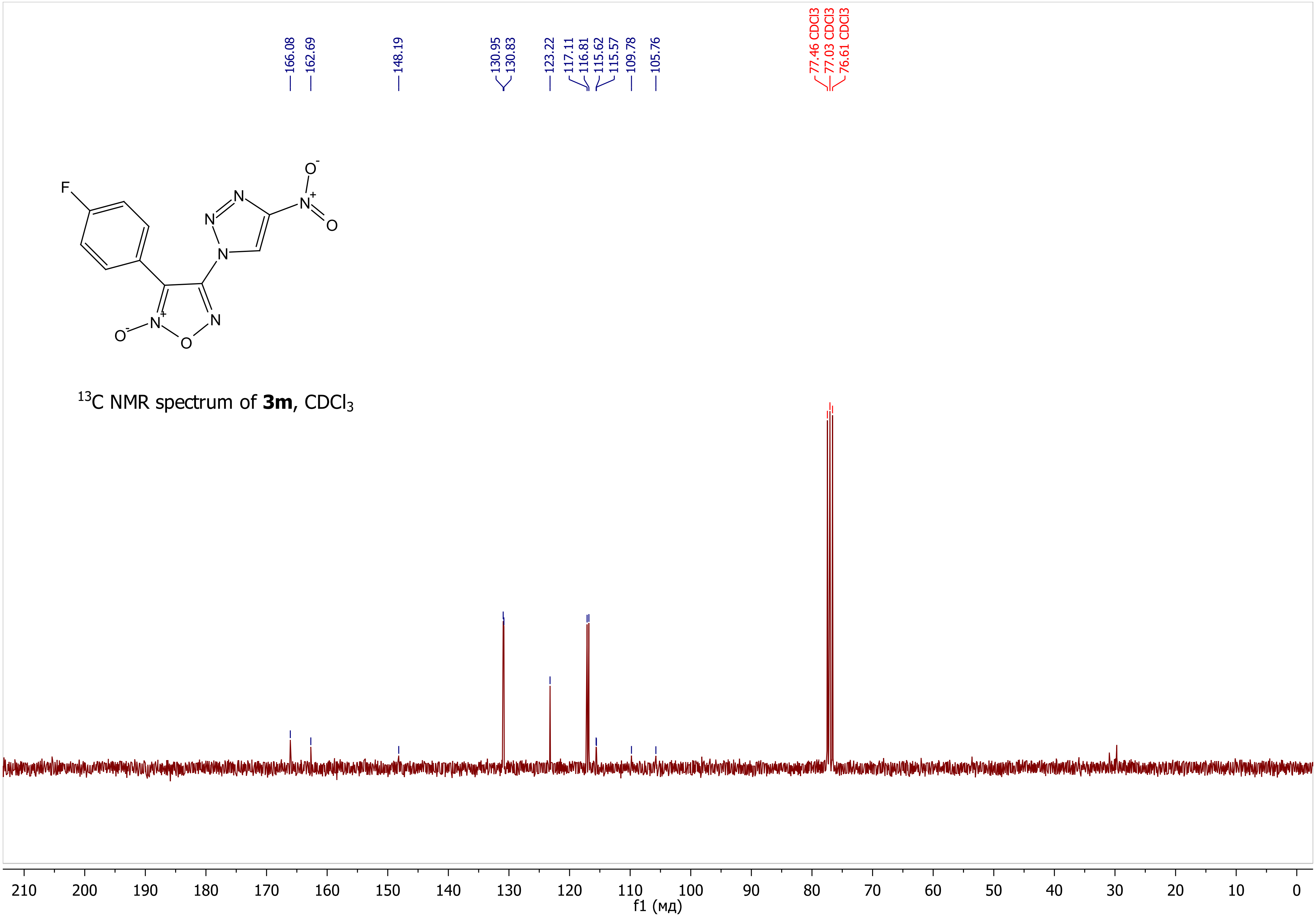

^{13}C NMR spectrum of **3m**, $CDCl_3$

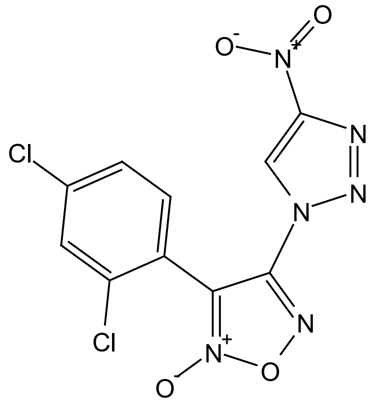

^{1}H NMR spectrum of **3n**, $CDCl_3$

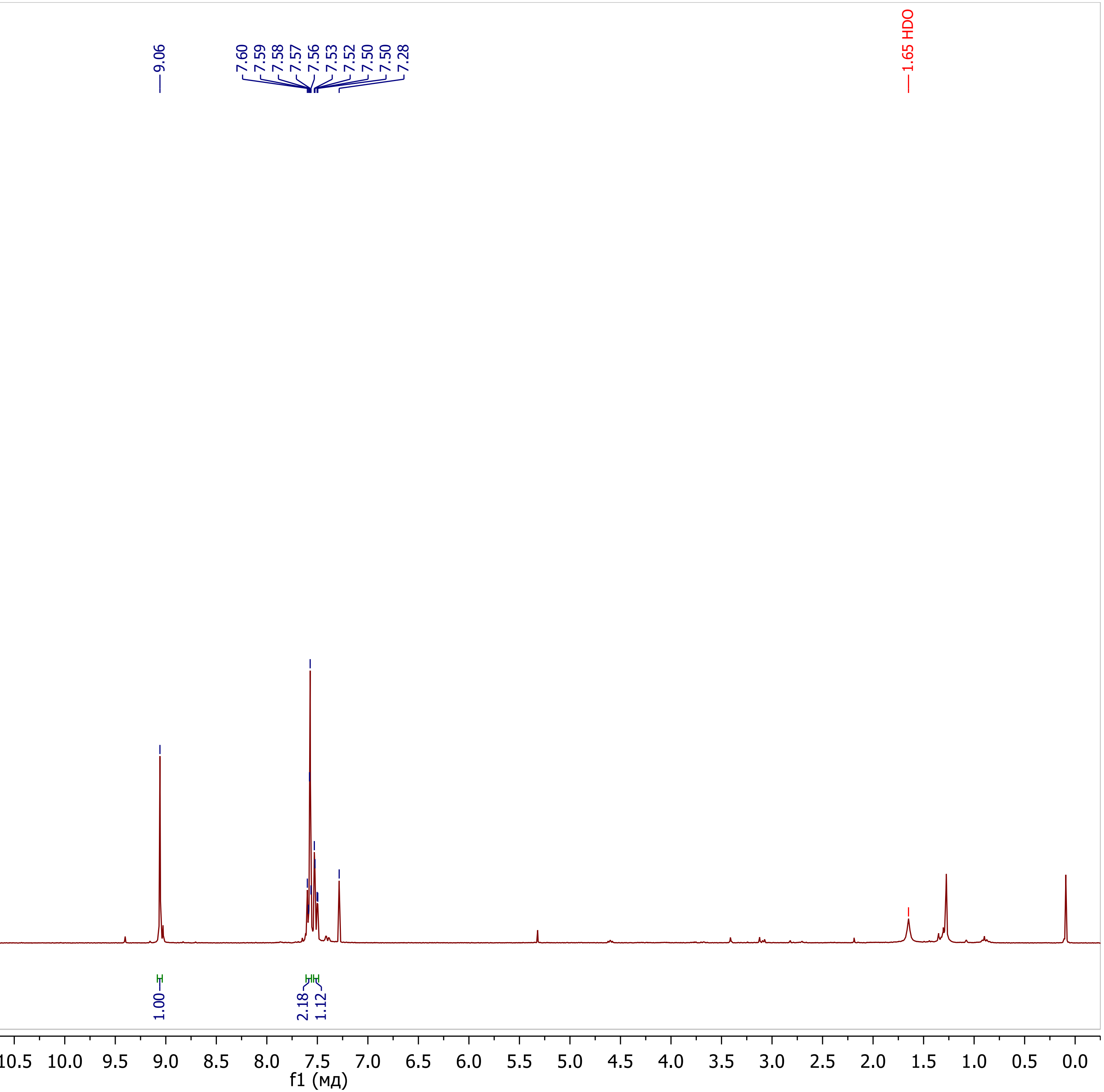

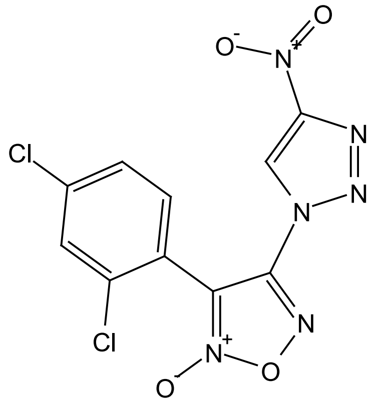

^{13}C NMR spectrum of **3n**, $CDCl_3$

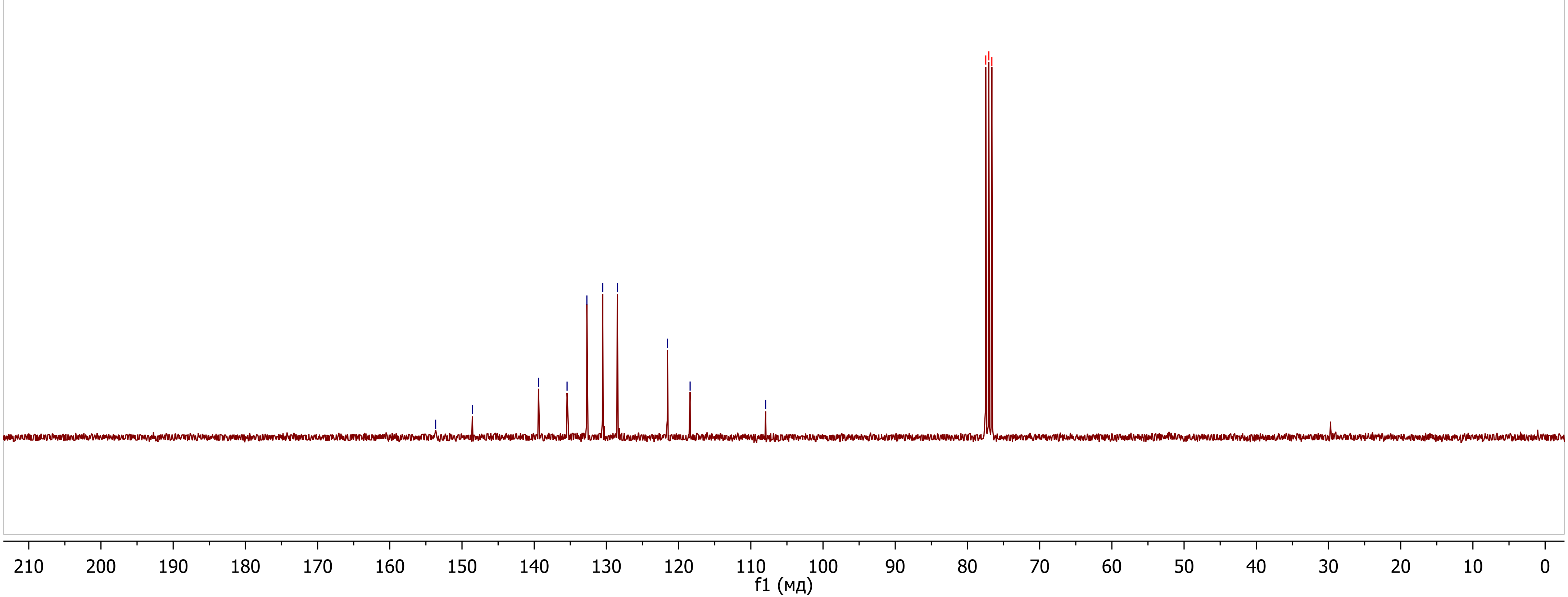

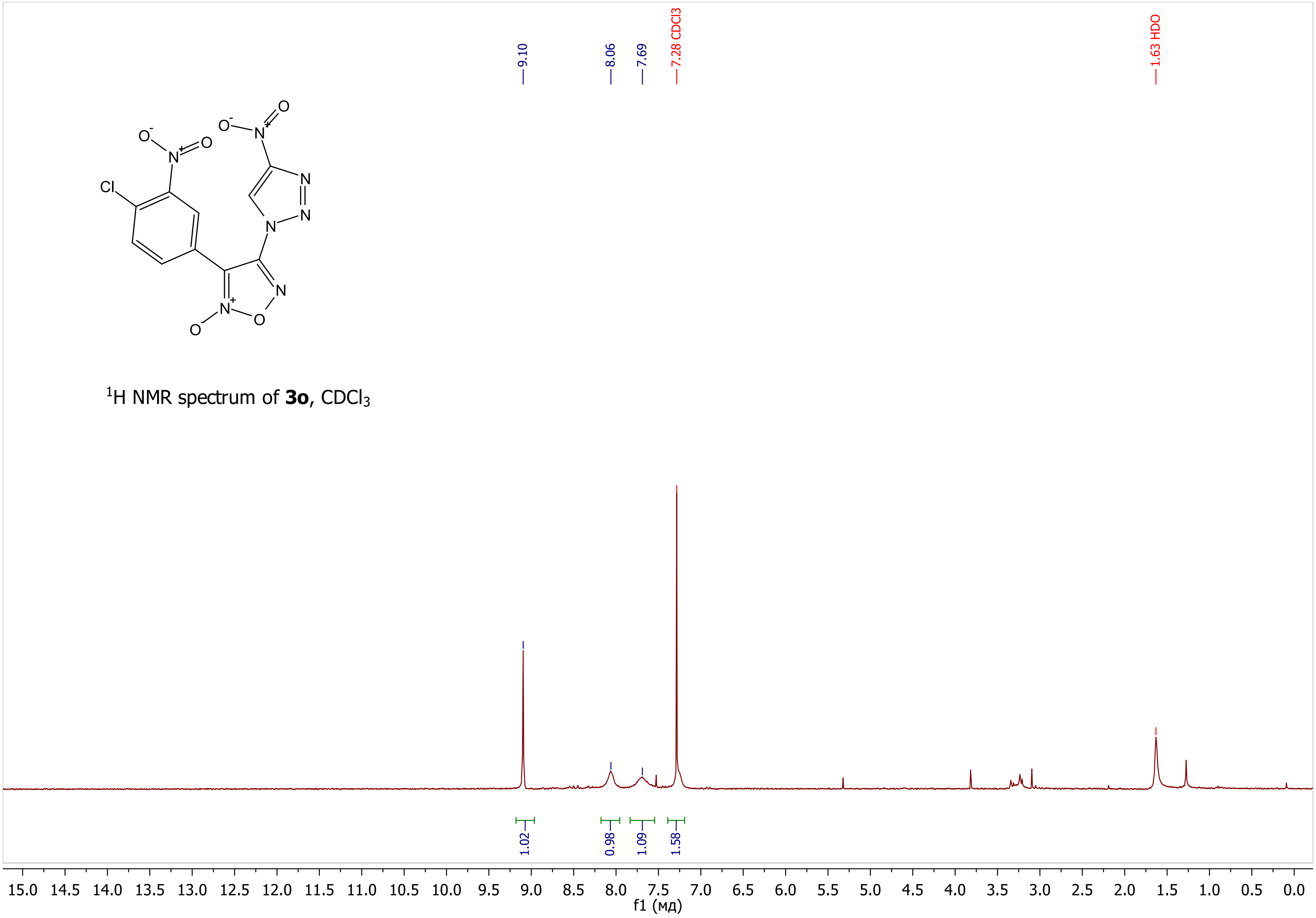

^{1}H NMR spectrum of **3o**, $CDCl_3$

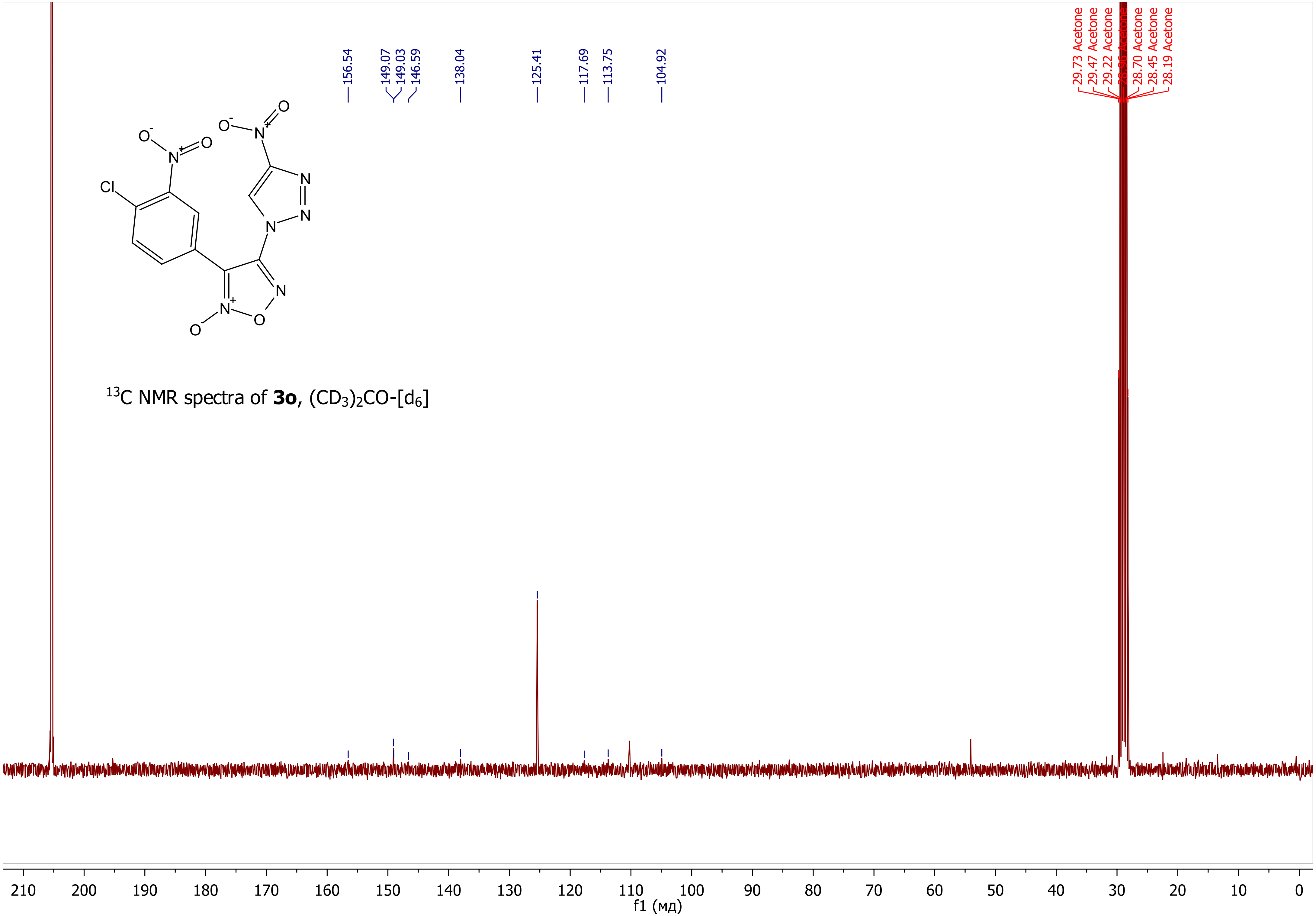

^{13}C NMR spectra of **3o**, $(CD_3)_2CO$-[d_6]

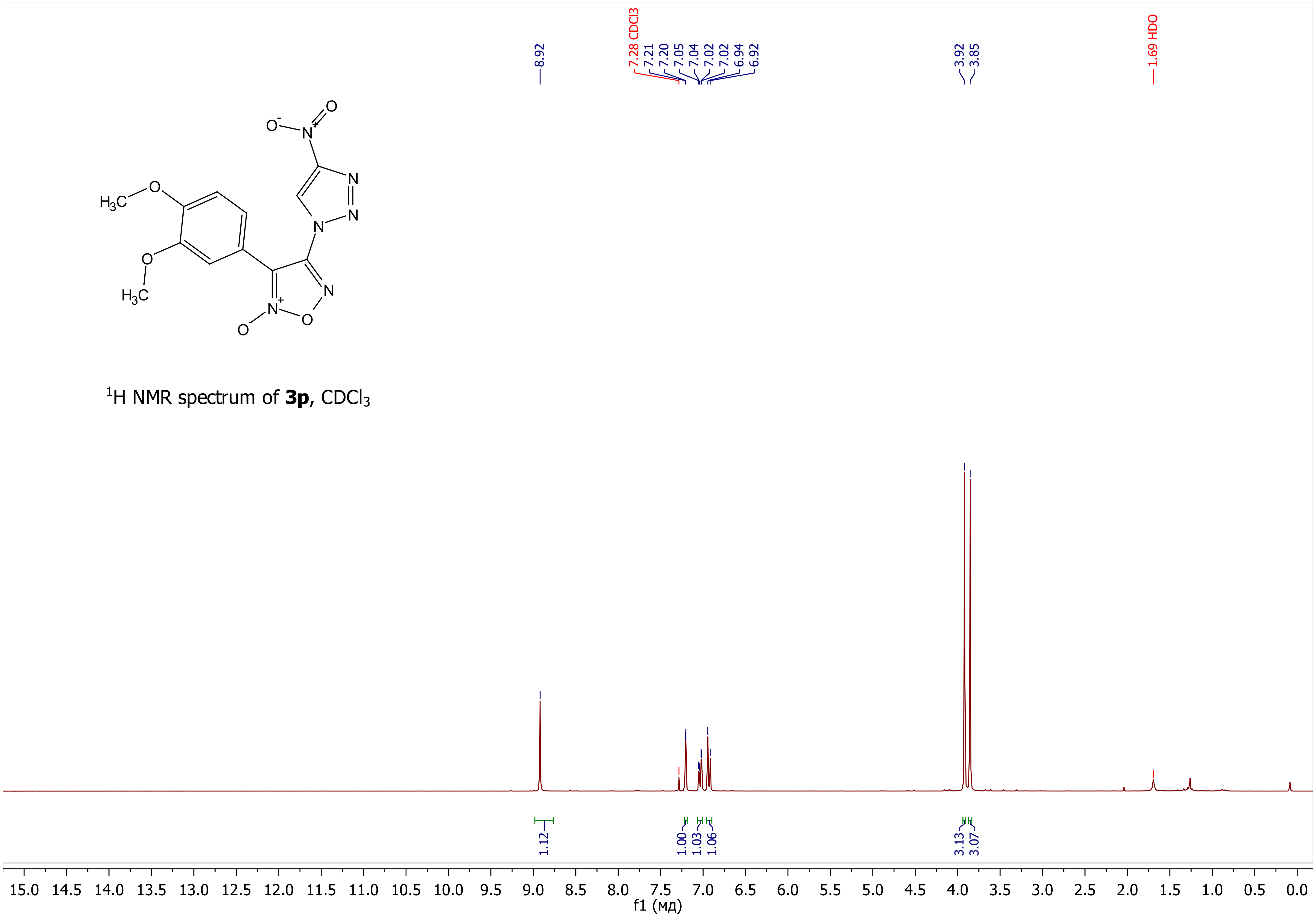

^{1}H NMR spectrum of **3p**, $CDCl_3$

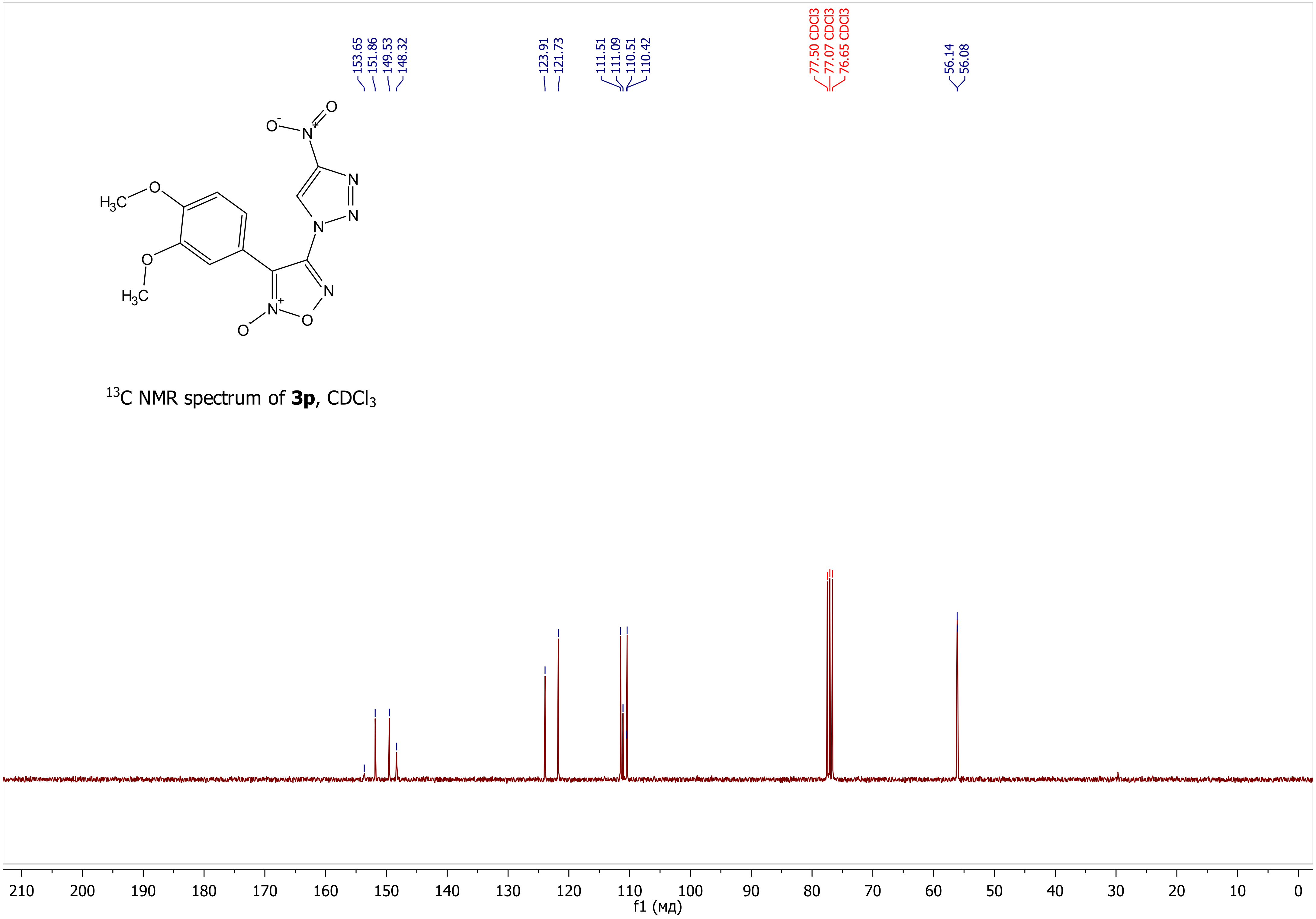

^{13}C NMR spectrum of **3p**, $CDCl_3$

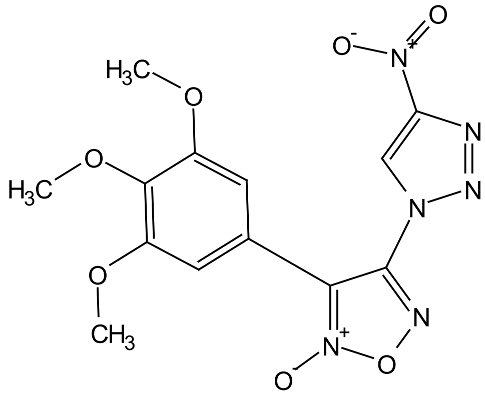

^{1}H NMR spectrum of **3q**, $CDCl_3$

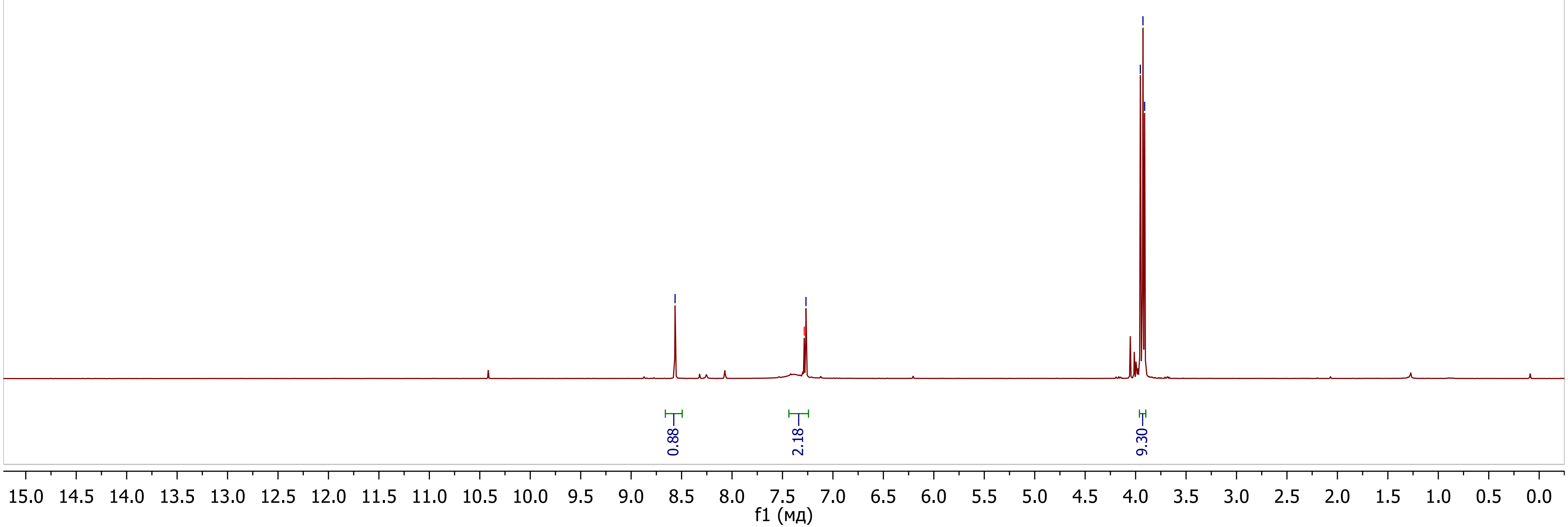

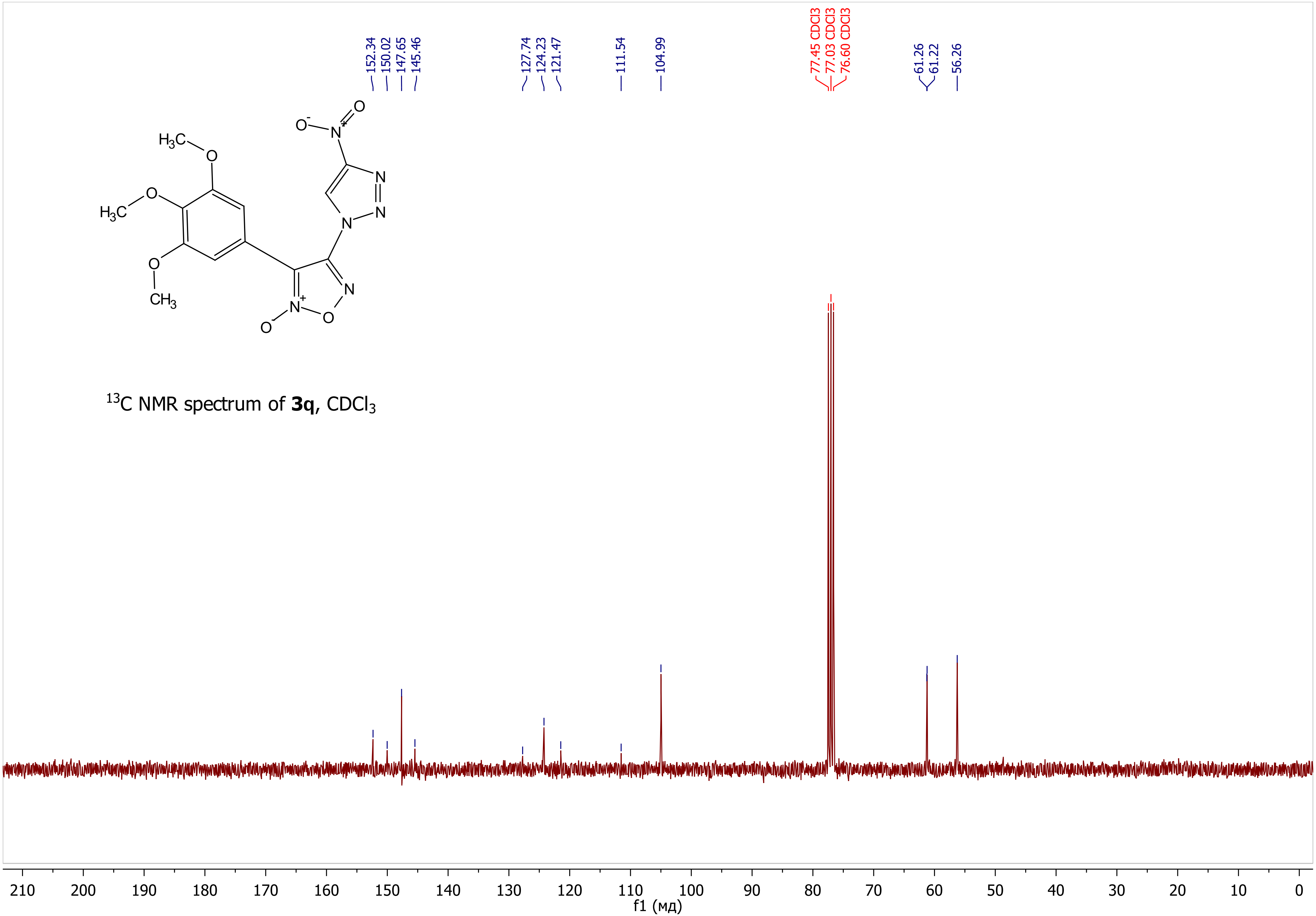

^{13}C NMR spectrum of **3q**, $CDCl_3$

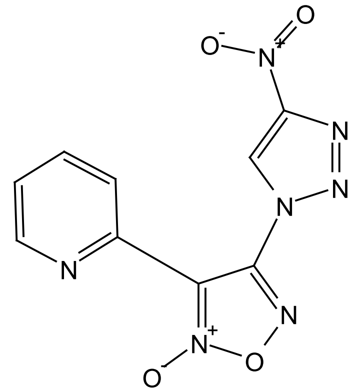

^{1}H NMR spectrum of **3r**, $CDCl_3$

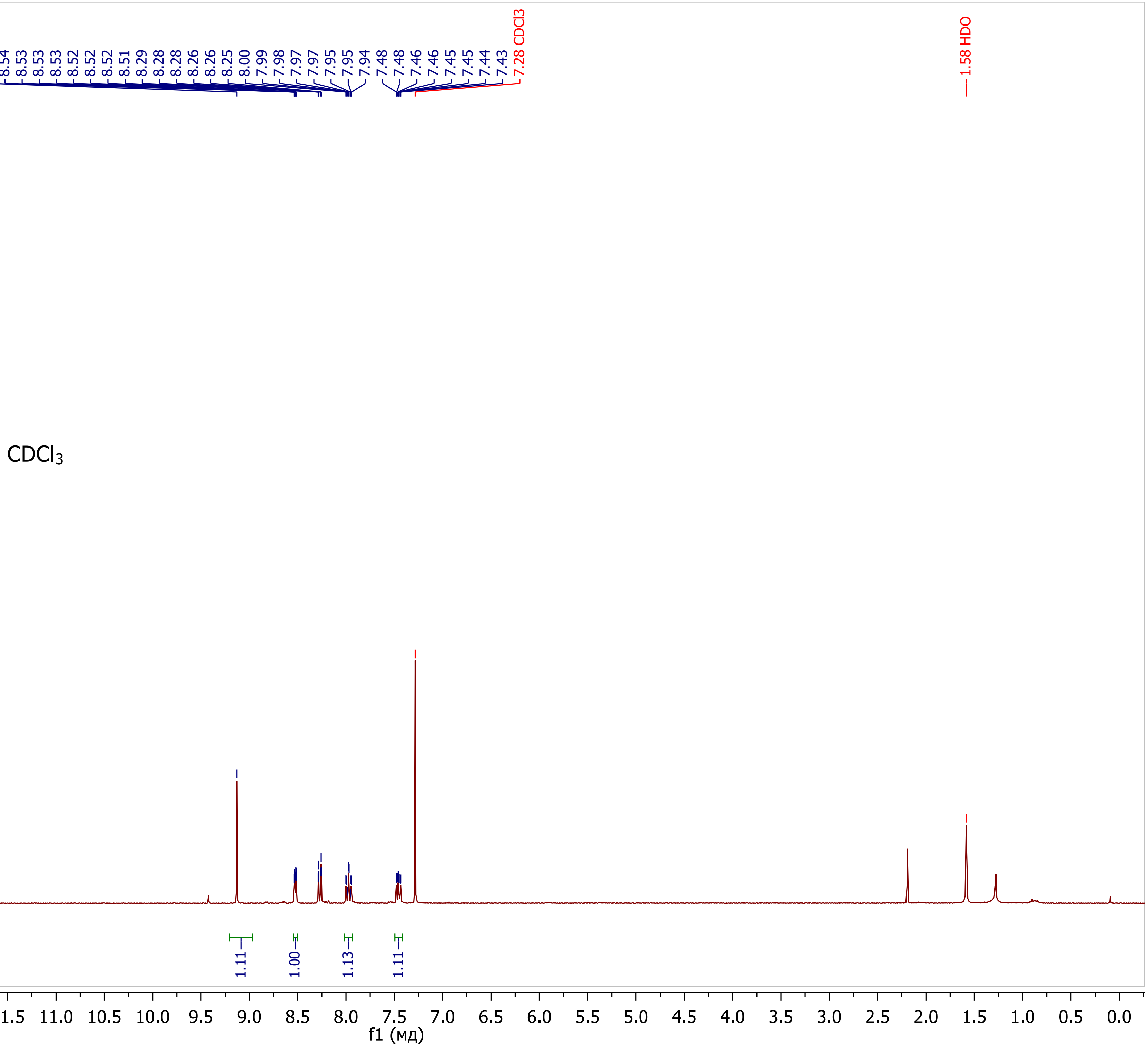

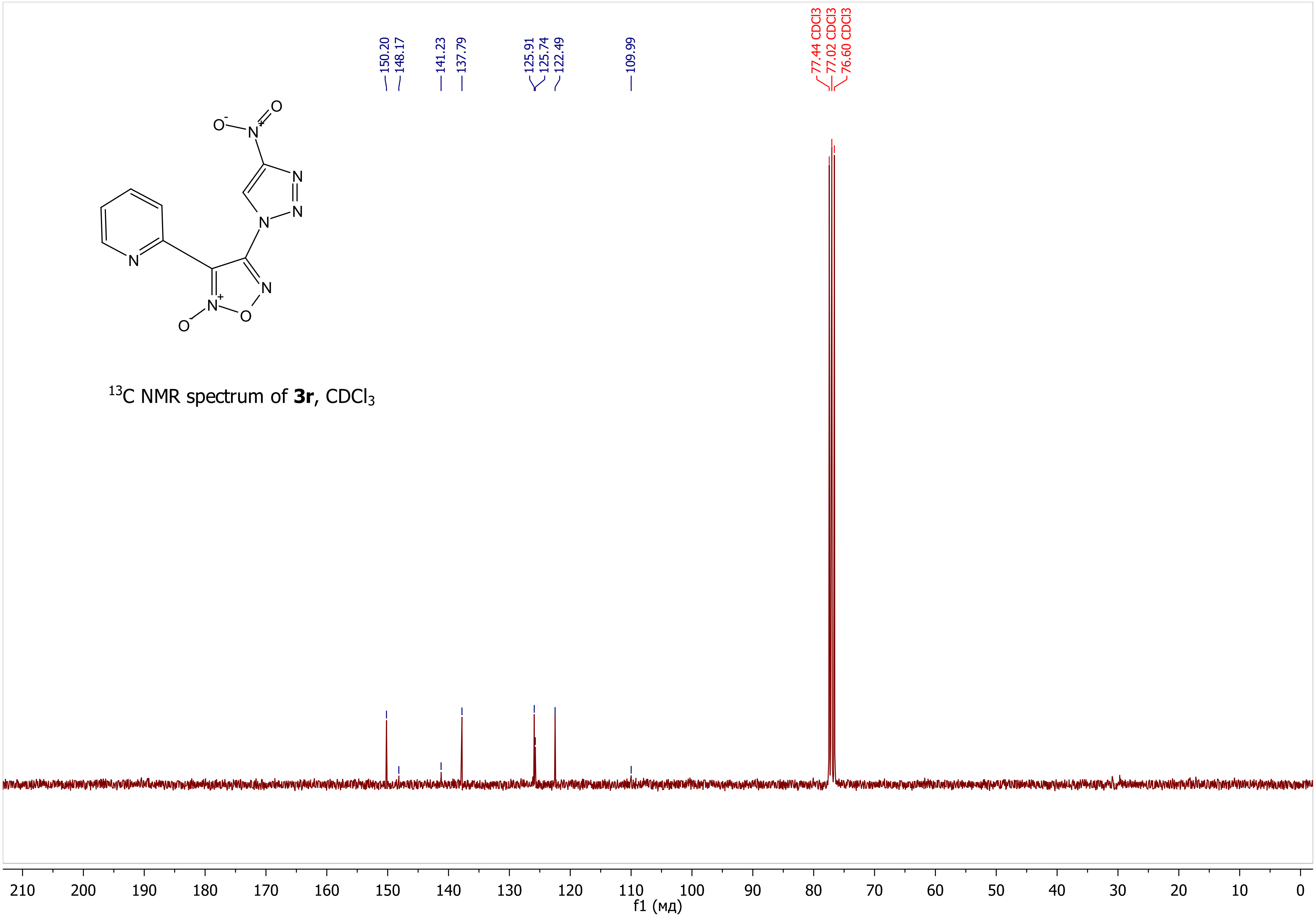

^{13}C NMR spectrum of **3r**, $CDCl_3$

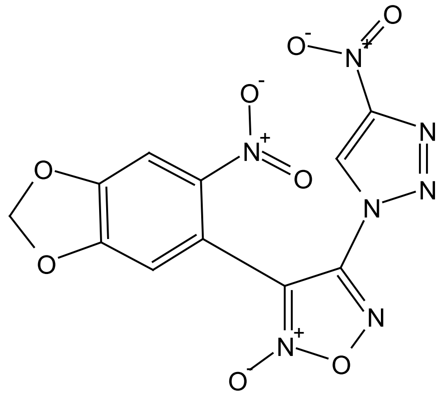

^{1}H NMR spectrum of **3s**, $CDCl_3$

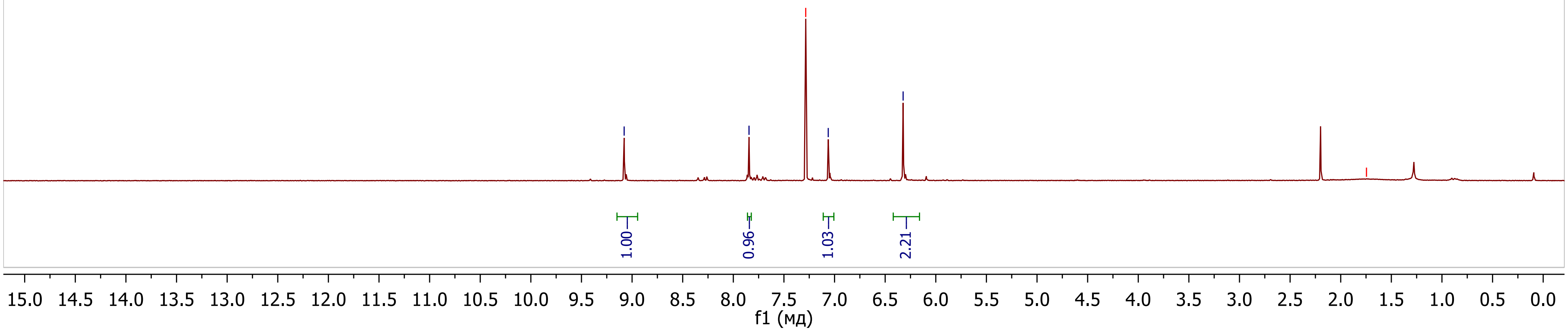

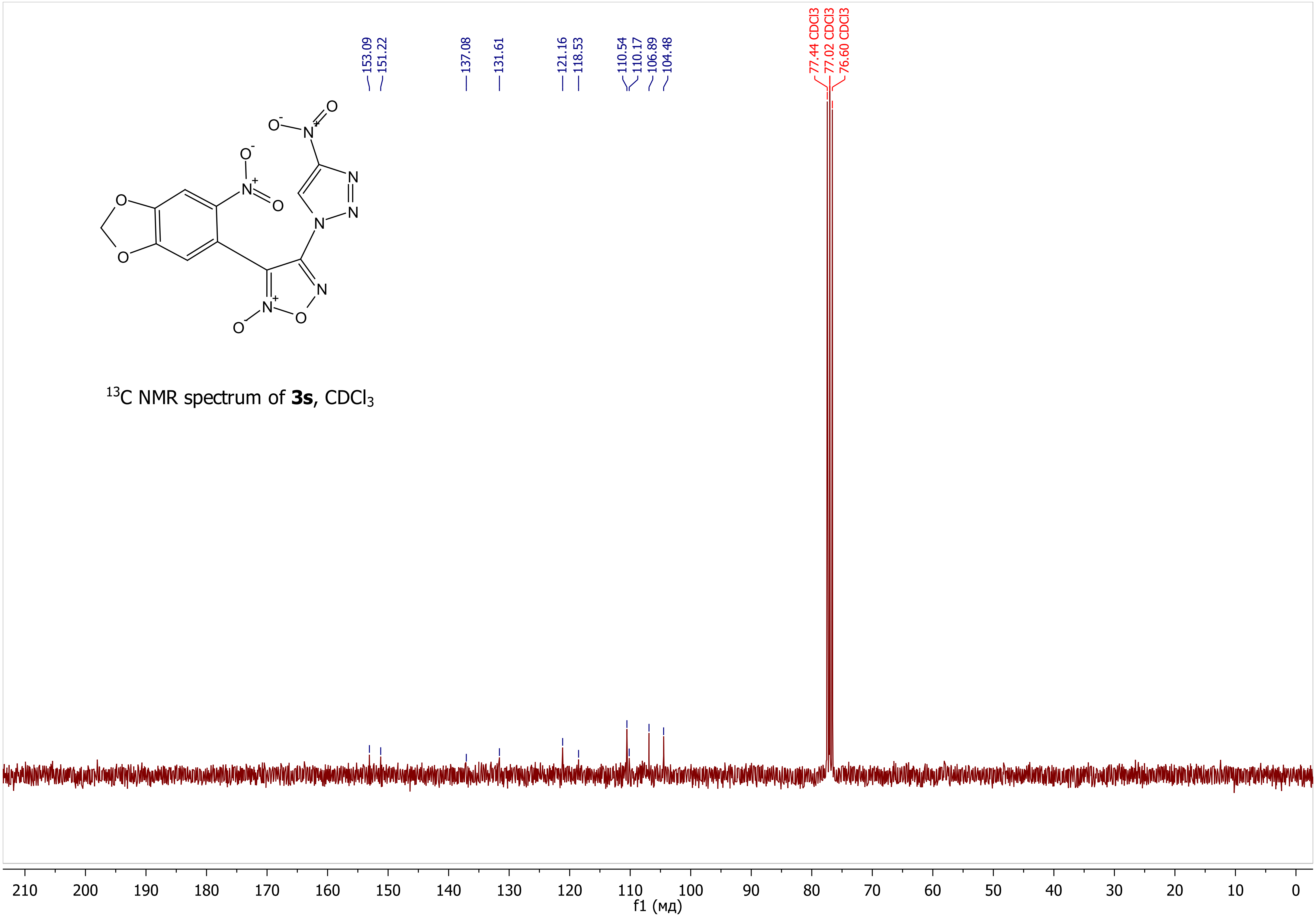

^{13}C NMR spectrum of **3s**, $CDCl_3$

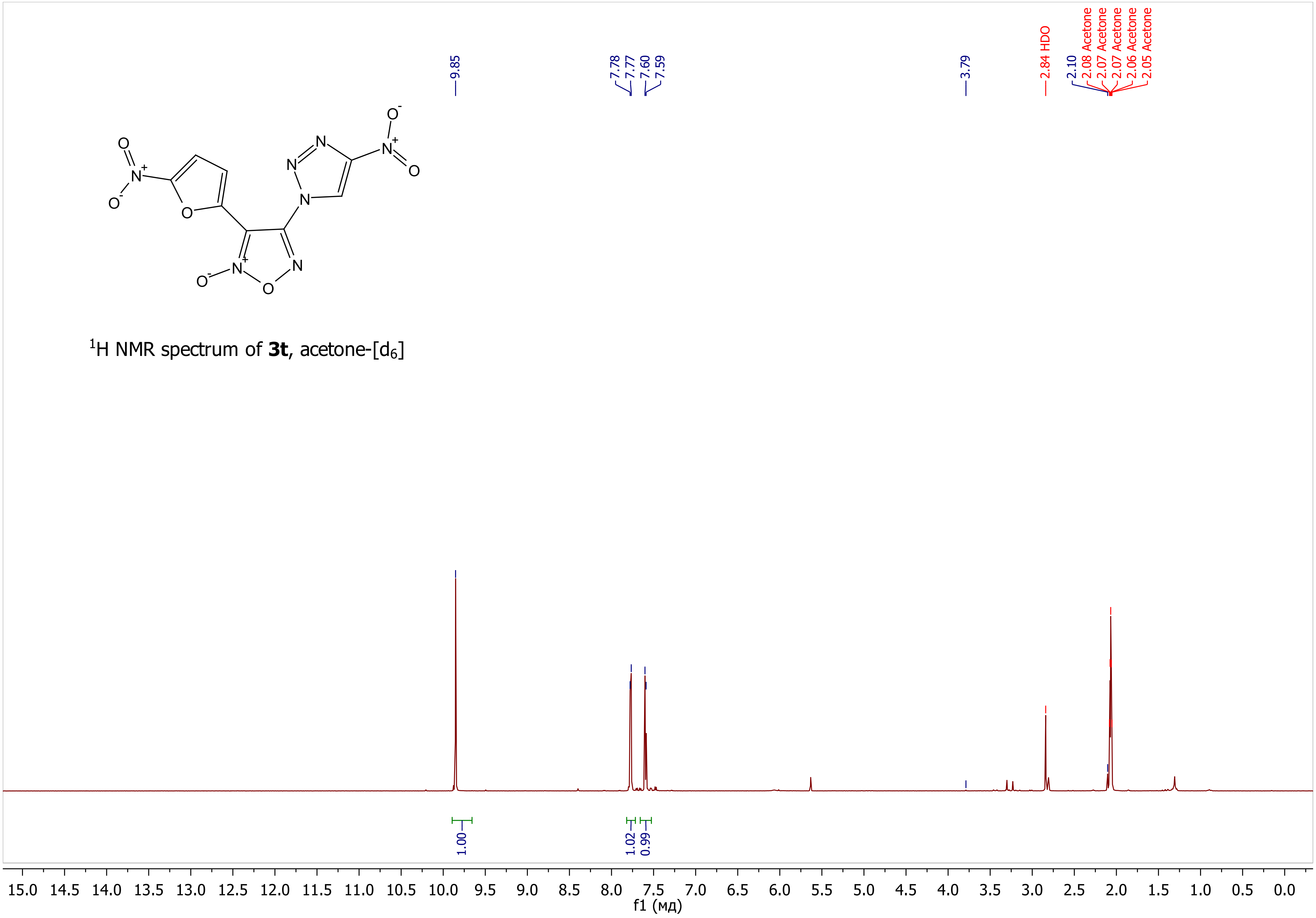

^{1}H NMR spectrum of **3t**, acetone-[d_6]

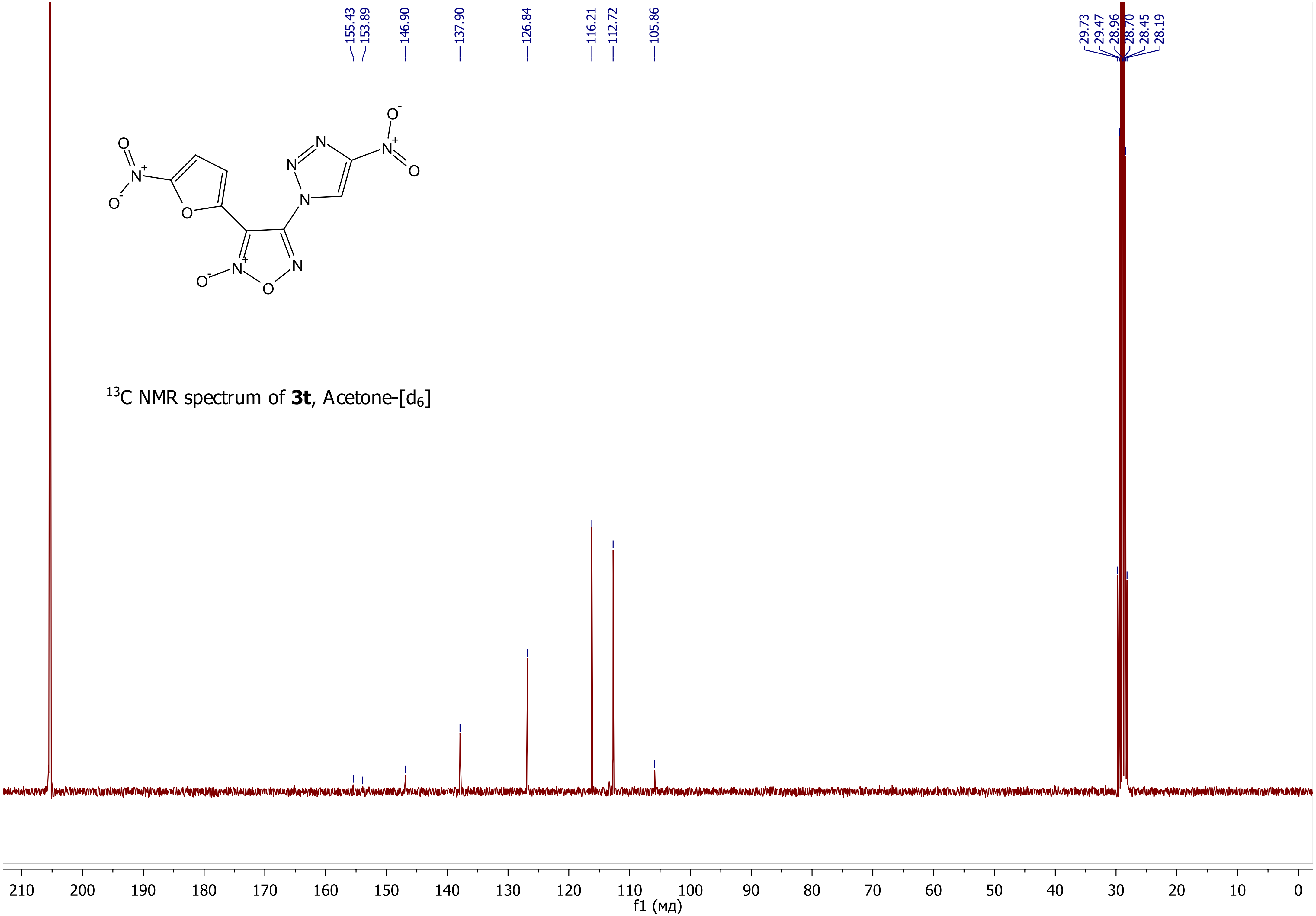

155.43
153.89
146.90
137.90
126.84
116.21
112.72
105.86
29.73
29.47
28.96
28.70
28.45
28.19
13C NMR spectrum of 3t, Acetone-[d6]
210 200 190 180 170 160 150 140 130 120 110 100 90 80 70 60 50 40 30 20 10 0
f1 (мд)

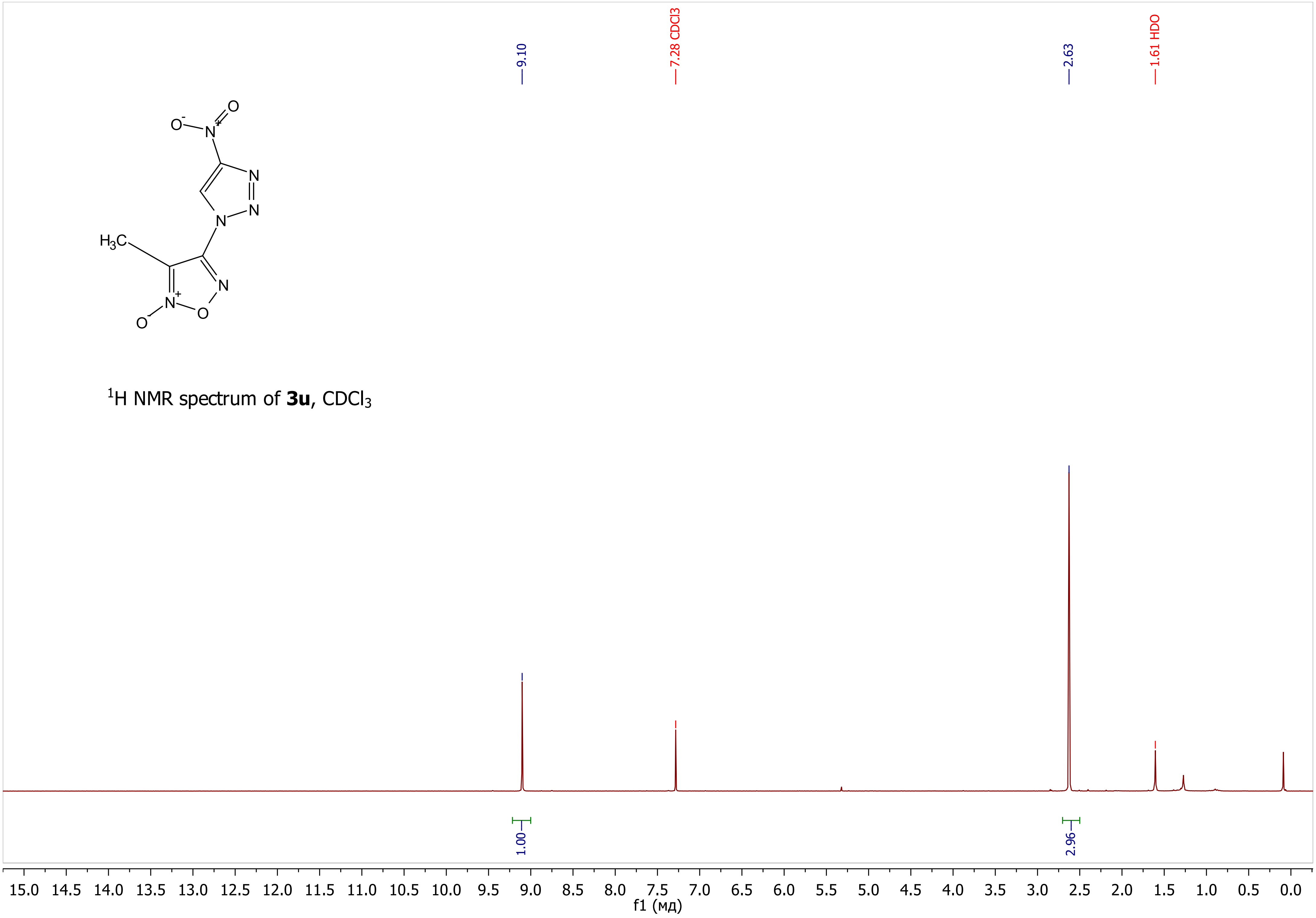

^{1}H NMR spectrum of **3u**, $CDCl_3$

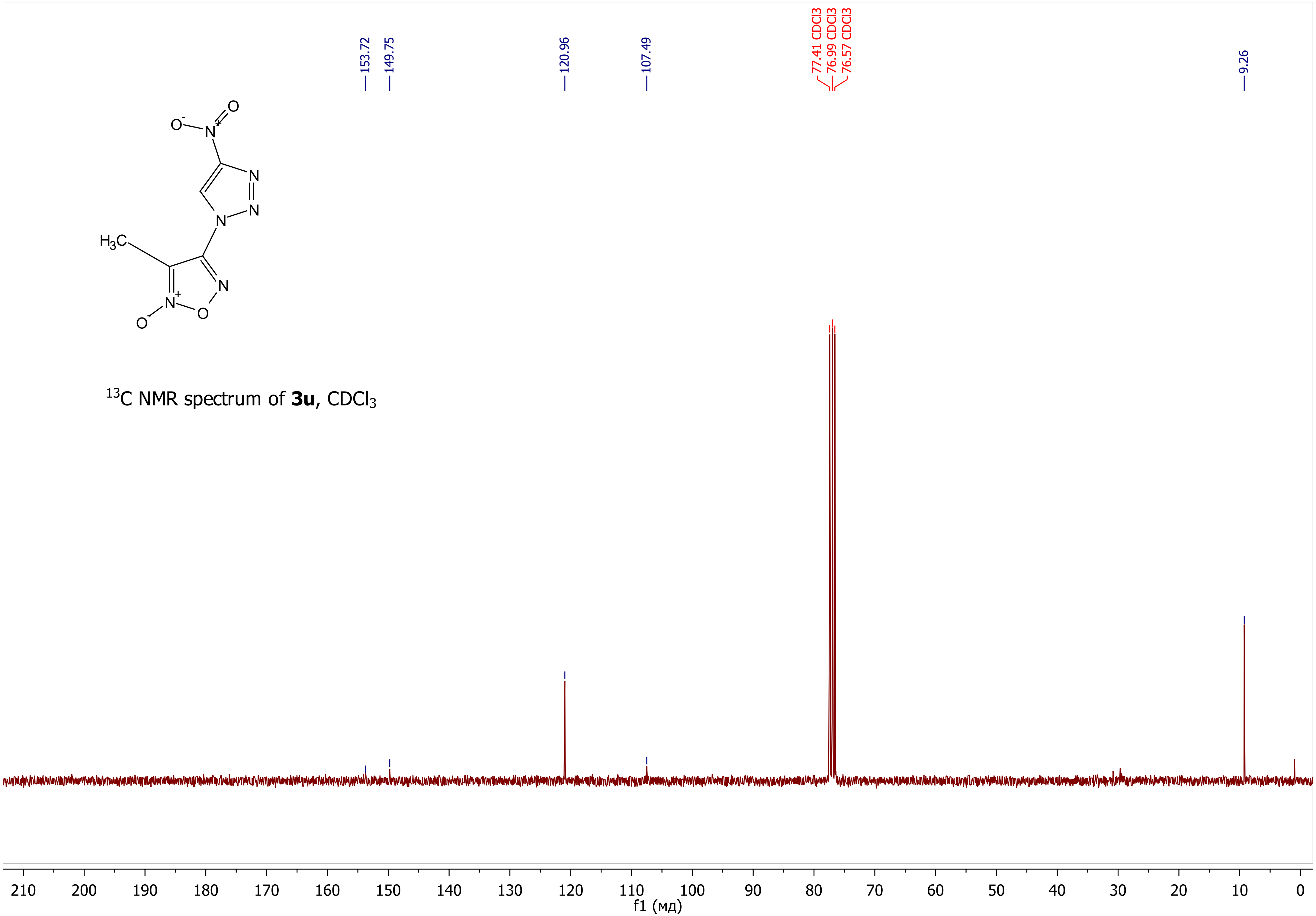

153.72
149.75
120.96
107.49
77.41 CDCl3
76.99 CDCl3
76.57 CDCl3
9.26
^{13}C NMR spectrum of **3u**, $CDCl_3$
210 200 190 180 170 160 150 140 130 120 110 100 90 80 70 60 50 40 30 20 10 0
f1 (мд)

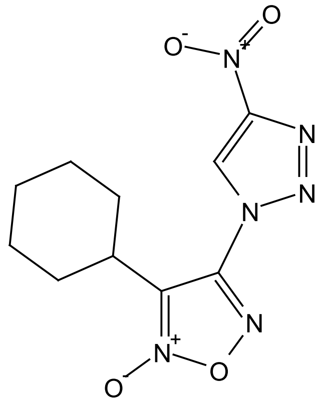

^{1}H NMR spectrum of **3v**, $CDCl_3$

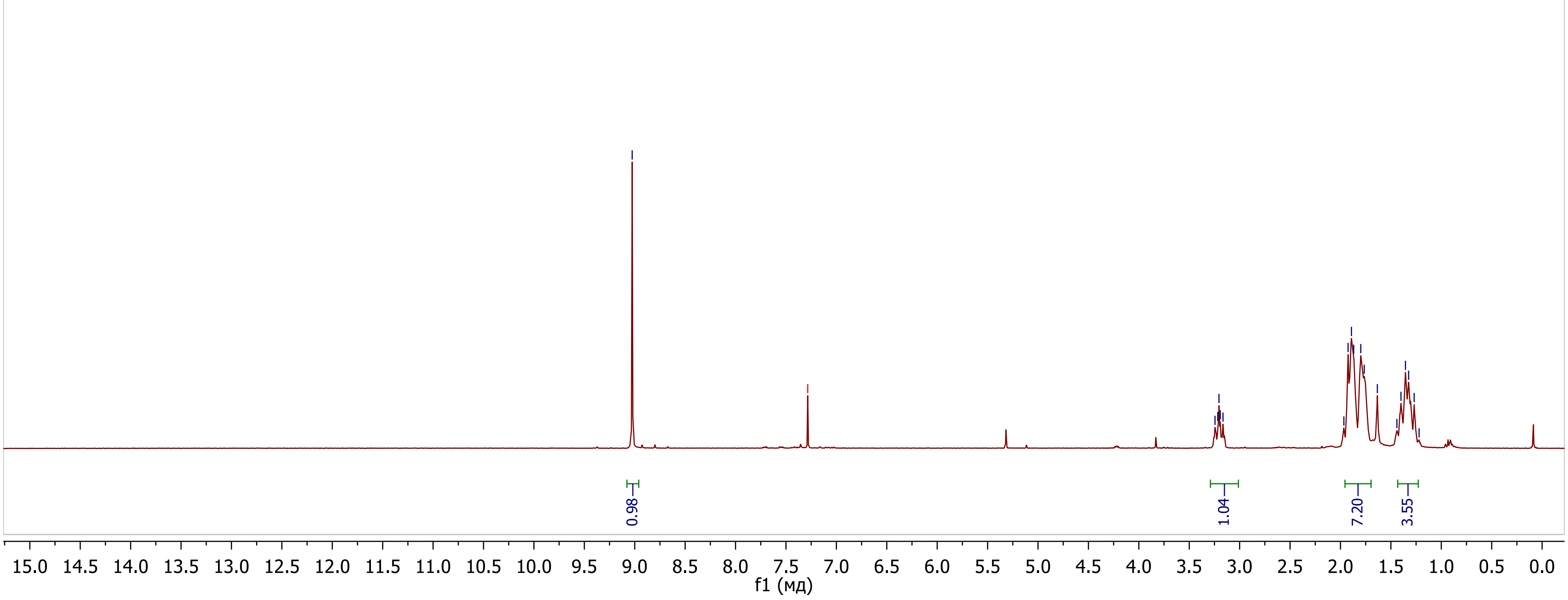

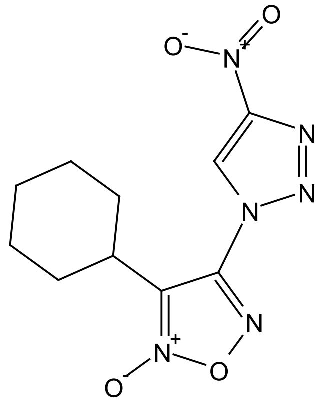

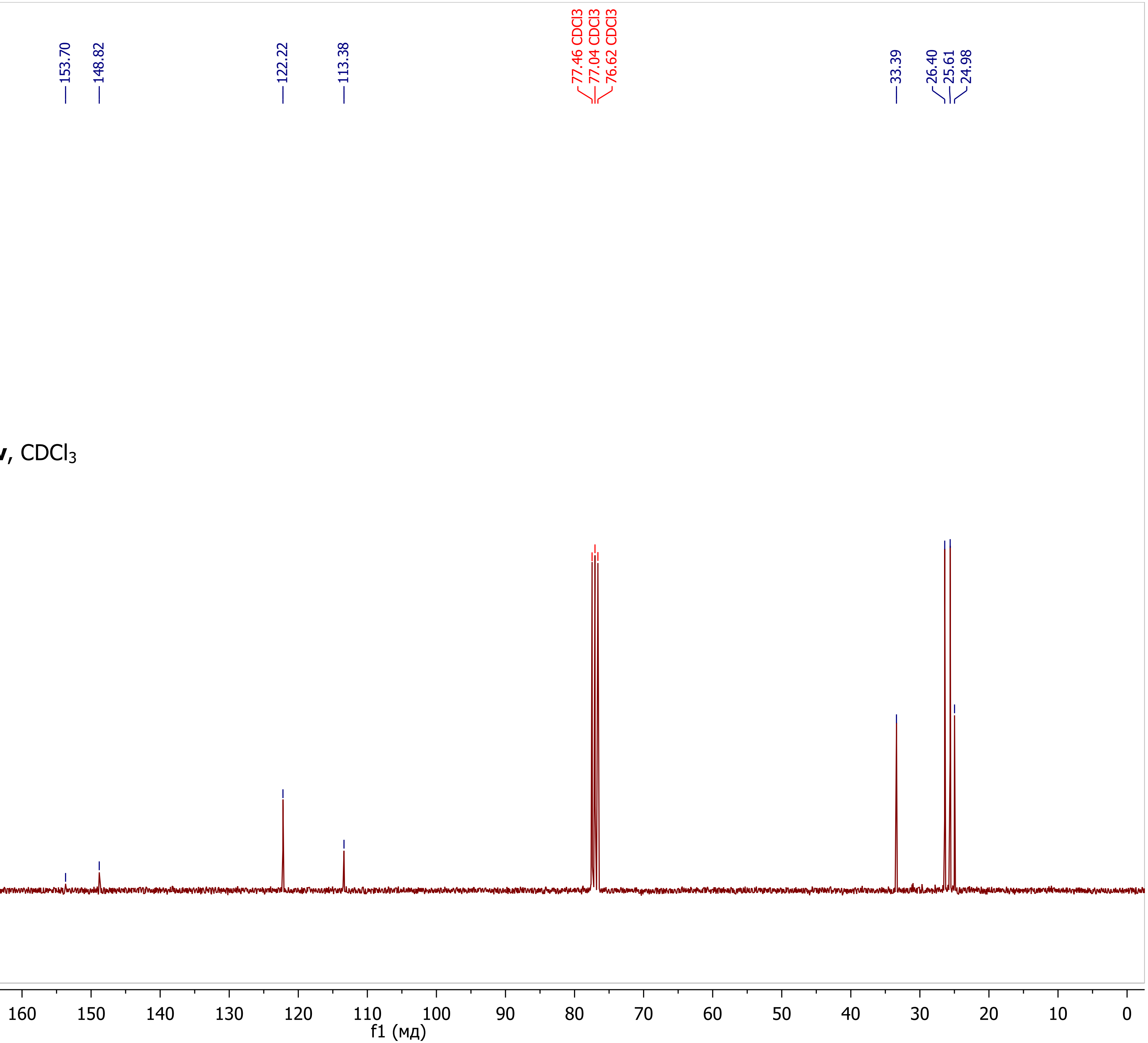

^{13}C NMR spectrum of **3v**, $CDCl_3$
